# Genome-wide association studies of root system architecture traits in a broad collection of *Brassica* genotypes

**DOI:** 10.3389/fpls.2024.1389082

**Published:** 2024-05-28

**Authors:** Chunxiao Yang, Rudolph Fredua-Agyeman, Sheau-Fang Hwang, Linda Y. Gorim, Stephen E. Strelkov

**Affiliations:** Department of Agricultural, Food and Nutritional Science, University of Alberta, Edmonton, AB, Canada

**Keywords:** *Brassica*, GWAS, marker-root trait associations, plant breeding, root system architecture

## Abstract

The root systems of *Brassica* species are complex. Eight root system architecture (RSA) traits, including total root length, total root surface area, root average diameter, number of tips, total primary root length, total lateral root length, total tertiary root length, and basal link length, were phenotyped across 379 accessions representing six *Brassica* species (*B. napus*, *B. juncea*, *B. carinata*, *B. oleracea*, *B. nigra*, and *B. rapa*) using a semi-hydroponic system and image analysis software. The results suggest that, among the assessed species, *B. napus* and *B. oleracea* had the most intricate and largest root systems, while *B. nigra* exhibited the smallest roots. The two species *B. juncea* and *B. carinata* shared comparable root system complexity and had root systems with larger root diameters. In addition, 313 of the *Brassica* accessions were genotyped using a 19K *Brassica* single nucleotide polymorphism (SNP) array. After filtering by TASSEL 5.0, 6,213 SNP markers, comprising 5,103 markers on the A-genome (covering 302,504 kb) and 1,110 markers on the C-genome (covering 452,764 kb), were selected for genome-wide association studies (GWAS). Two general linear models were tested to identify the genomic regions and SNPs associated with the RSA traits. GWAS identified 79 significant SNP markers associated with the eight RSA traits investigated. These markers were distributed across the 18 chromosomes of *B. napus*, except for chromosome C06. Sixty-five markers were located on the A-genome, and 14 on the C-genome. Furthermore, the major marker-trait associations (MTAs)/quantitative trait loci (QTLs) associated with root traits were located on chromosomes A02, A03, and A06. *Brassica* accessions with distinct RSA traits were identified, which could hold functional, adaptive, evolutionary, environmental, pathological, and breeding significance.

## Introduction

Roots are a fundamental component of the plant vascular system, playing a pivotal role in plant growth, development, and overall survival. The primary function of roots is absorbing water and nutrients from the soil, as well as serving as an anchor that firmly secures plants to the ground, thereby ensuring stability and support. The development of a root system is an important quantitative characteristic that determines a plant’s capacity to survive across different environments. Improved understanding of the behavior of roots within natural ecosystems is of significance in enhancing crop yields, developing more resilient plant varieties, and preserving biodiversity (Griffiths et al., 2022).

The genus *Brassica* consists of 37 species including the widely cultivated commercial crops *Brassica napus* L. (AACC, n = 19), *B. rapa* L. (AA, n = 10), *B. nigra* (L.) Koch (BB, n = 8), *B. oleracea* L. (CC, n = 9), *B. juncea* (L.) Czern & Coss (AABB, n = 18), and *B. carinata* A. Braun (BBCC, n = 17) (Branca and Cartea, 2011). Most *B. juncea* and *B. nigra* genotypes have fibrous roots (Center for Agriculture, Food, and the Environment, 2015), while *B. napus* (Arif et al., 2019), *B. rapa* (Center for Agriculture, Food, and the Environment, 2015), *B. oleracea* (Center for Agriculture, Food, and the Environment, 2015), and *B. carinata* (Barro and Martín, 1999) have a large taproot system with a single main root axis and hundreds of lateral roots. Several root system architecture (RSA) traits are heritable characteristics, which suggests some level of genetic control mechanisms that may be exploited in breeding programs (Shi et al., 2013). In addition, root growth and development have been reported to be influenced by plant hormonal cues (Tanimoto, 2005; Santner et al., 2009; Vissenberg et al., 2020). The uneven distribution of nutrients and water in soils as well as soil microbiomes have also been reported to influence root growth and development (Molefe et al., 2023). Lastly, the plasticity of roots make them vulnerable to physico-chemical environmental influences (Lynch, 2007; Ingram and Malamy, 2010; Yang et al., 2010; Shi et al., 2013; Zhang et al., 2016; Ibrahim et al., 2021).

Genetic variability in plants is a fundamental component of biodiversity and serves as the foundation for the creation of novel and enhanced cultivars with desirable traits (Govindaraj et al., 2015; Yu et al., 2021). Therefore, increasing the genetic diversity in *Brassica* genotypes is an important breeding objective. However, only a few studies have reported the potential of root phenomics for crop breeding (Kuijken et al., 2015; Prince et al., 2019; Falk et al., 2020; Liu et al., 2021). This is mostly due to the complexity and large genetic variability of *Brassica* root systems, the difficulties of accessing intact roots, as well as the tedious and laborious nature of phenotyping *Brassica* roots (de Dorlodot et al., 2007; Zhu et al., 2011; Meister et al., 2014; Ibrahim et al., 2021; Yang et al., 2024). The main goal of breeding programs that target root traits is to enhance the ability of roots to explore the soil and acquire water and nutrients, and to develop crops with increased stress tolerance and improved yields. By improving understanding of RSA and the underlying pathways that shape it, researchers can leverage diverse root features to help plants respond to climate change and enhance crop yields (Smith and De Smet, 2012; Ibrahim et al., 2021). However, genetic studies focusing on RSA, especially of *Brassica* species, are limited compared with research on aboveground traits. Most of these studies were conducted in controlled environments, but have value since they enable the observation of RSA and the collection of intact roots (Wang et al., 2019). For example, the detection of overlapping quantitative trait loci (QTLs) for root traits under non-soil-based conditions alongside those influencing yield in the field suggested the relevance and applicability of the non-soil methods (Tuberosa et al., 2002; Wang et al., 2019).

The limited genetic studies on *Brassica* RSA have focused primarily on the important role of root morphology in enhancing the efficiency of phosphorus, boron, and nitrogen uptake in rapeseed (*B. napus*) (Yang et al., 2010; Shi et al., 2013; Zhang et al., 2016; Wang et al., 2017, 2019). Alcock et al. (2018) reported that in *B. napus*, the chromosomal regions associated with foliar phosphorus concentration harbor multiple genes that influence root development. Furthermore, homologs of three ABC transporter proteins implicated in root suberin synthesis exhibited co-localization with peaks associated with leaf nitrate and phosphorus levels. In another RSA study, Ahmad et al. (2023) identified 39 significant single nucleotide polymorphisms (SNPs) on *Brassica* chromosomes A04, A09, A10, C02, C03, C07, and C09 that were associated with RSA and biomass traits in 327 rapeseed accessions under low phosphorus conditions. Fletcher et al. (2016) also reported 20 QTLs on chromosomes A03, A10 and C02, associated with flowering time and root mass in a *B. napus* doubled haploid population, demonstrating their association with adaptation to drought.

Recently, we identified total root length (TRL/cm), total root surface area (TRSA/cm^2^), root average diameter (RAD/cm), number of tips (NTP), total primary root length (TPRL/cm), total lateral root length (TLRL/cm), total tertiary root length (TTRL/cm), and basal link length (BLL/cm) as significant RSA traits in the *B. napus* root system (Yang et al., 2024). The genomic regions controlling the observed phenotypic differences, however, were not determined. Genome-wide association studies (GWAS) is a method rooted in the concept of linkage disequilibrium (LD), which detects associations between genotypic and measured phenotypic data. This is achieved by examining differences in allele frequency of genetic variants within natural populations (Uffelmann et al., 2021). This approach offers notable advantages compared with traditional linkage-based association mapping (Gupta, 2016; Fredua-Agyeman et al., 2020). GWAS enable the exploration of a broader range of allelic diversity, providing enhanced resolution for analyzing various traits of interest. In addition, GWAS also offer the opportunity to examine genotypes across different crop species, eliminating the need for ancestry or pedigree data typically required in QTL mapping (Fredua-Agyeman et al., 2020).

In the present study, we assessed eight RSA traits in 379 accessions representing the six commercially important *Brassica* species, *B. napus*, *B. juncea*, *B. carinata*, *B. oleracea*, *B. nigra*, and *B. rapa*. Additionally, we identified the genomic regions associated with these key RSA traits through GWAS.

## Materials and methods

### Plant materials

Three hundred and forty-one genotypes, primarily landraces, were included in this study. These genotypes consisted of 68 *B. napus*, 64 *B. juncea*, 60 *B. rapa*, 66 *B. nigra*, 55 *B. oleracea* and 28 *B. carinata* accessions obtained from the Leibniz Institute of Plant Genetics and Crop Plant Research (IPK), Gatersleben, Germany (**Supplementary Table 1**). An additional 25 Canadian canola (*B. napus*) cultivars and the 13 hosts of the Canadian Clubroot Differential (CCD; Strelkov et al., 2018) were also included in the experiments. Accessions obtained from IPK were multiplied under greenhouse conditions at the Crop Diversification Centre North, Alberta Agriculture and Irrigation, Edmonton, Canada.

### Growth conditions and RSA traits phenotyping

Plants were grown and phenotyped following Yang et al. (2024). In brief, 7-day-old seedlings that had been pre-germinated in Petri dishes were moved to a semi-hydroponic system. This system comprised rolls of germination paper (Anchor Paper Company, St. Paul, USA) placed in 2 L beakers filled with 1 L of half-strength Hoagland’s No. 2 Basal Salt Mixture solution (Sigma-Aldrich Co., Ontario, Canada). The seedlings were maintained in a growth chamber under a 16 h photoperiod at 20^°^C (day)/18^°^C (night) and removed from the germination paper after 14-days to measure RSA traits (Yang et al., 2024). The roots of each plant were severed, arranged on a scanning tray, and gently spread apart using forceps. Subsequently, root scans were performed using an EPSON Perfection V800 scanner (Epson, Markham, ON). The analysis was done with the aid of WinRHIZO^™^ software (Regent Instruments Inc., Quebec, Canada), employing the linkage analysis for root and background detection. Twenty-nine RSA-related traits were recorded per root scan, only eight of which showed significant variation within and among genotypes and hence were employed for GWAS (Yang et al., 2024). The eight traits included (1) total root length (TRL/cm), (2) total surface area of roots (TRSA/cm^2^), (3) root average diameter (RAD/cm), (4) number of tips (NTP), (5) total primary root length (TPRL/cm), (6) total lateral root length (TLRL/cm), (7) total tertiary root length (TTRL/cm), and (8) basal link length (BLL/cm). The experiment was performed four times, with each experiment consisting of four replicates.

### Statistical analyses

Analysis of the RSA traits data was conducted with R 4.0.2: A Language and Environment for Statistical Computing (R Foundation for Statistical Computing, Vienna, Austria). Because repetition × treatment was not significant (*p*-value ranging from 0.86 to 0.95), the data were pooled across the four repeats of the experiment. Duncan’s multiple range test (Duncan, 1955) and the bar plot of each trait were generated to test (*p*-value ≤ 0.05) for differences in the mean root trait values among the six *Brassica* species. The Anderson-Darling test (Anderson and Darling, 1954) was performed to test the normality of the eight traits. The Spearman rank-based variable correlation test (Conover, 1971) was conducted using the *cor* function to determine the correlation among root traits (*p*-value ≤ 0.05). Principal component analysis (PCA) (Pearson, 1901) was carried out using the *prcomp* function on the eight variables.

### SNP genotyping

SNP genotyping was conducted on 313 of the 379 *Brassica* accessions, excluding the 66 *B. nigra* accessions, using the *Brassica* 19K SNP array from TraitGenetics GmbH (Gatersleben, Germany), according to the manufacturer’s instructions. This array comprised 9,310 SNP markers on the A-genome, 8,072 SNP markers on the C-genome, and 1,154 SNP markers on scaffolds. None of the markers on the array was from the B-genome, and hence the *B. nigra* accessions were not genotyped. After discarding monomorphic, low coverage site markers, markers with MAF ≤ 0.05 and those missing data for > 5% of the accessions, 6,213 SNP markers, comprising 5,103 A-genome and 1,110 C-genome markers, were used for GWAS. The average inter-SNP marker distance was determined for each chromosome.

### Linkage disequilibrium estimation

The non-random occurrence of genetic variants among the RSA traits detected as LD between alleles at different loci was estimated using Pearson’s squared correlation coefficient (*r* ^2^) statistic with TASSEL 5 v5.2.2.5 (Bradbury et al., 2007). The decay and extent of LD was determined by calculating the Chi-square (χ^2^) statistic for each SNP pair following Fredua-Agyeman et al. (2020). In brief, the *r* ^2^ -values of significant (*p*-value ≤ 0.001) SNP marker pairs was plotted against the physical distance (in megabases (Mb)) for each chromosome using the PROC GPLOT procedure in SAS v. 9.4 (SAS Institute, Cary NC, North Carolina, U.S.). The PROC TRANSREG function in SAS was then used to obtain an LD decay curve for each chromosome. Additionally, the intersection between the fitted curve and the *r* ^2^-value threshold line were determined and projected onto the physical distance axis to obtain the average extent of LD for each chromosome (Breseghello and Sorrells, 2006; Bellucci et al., 2015).

### Bayesian population structure analysis

The population structure was determined based on the method of Yu et al. (2021). In brief, a series of *STRUCTURE* analyses were run using the admixture and allele frequency correlated models, with burn-in lengths ranging from 5,000 to 100,000 iterations and Markov Chain Monte Carlo (MCMC) run lengths from 5,000 to 100,000 permutations, using *STRUCTURE* v2.3.4 (Pritchard et al., 2000). Runs for each cluster (*K* = 1–10) were replicated 10 times. The number of clusters was determined using the ΔK statistics of Evanno et al. (2005), and the MedMedK, MedMeaK, MaxMedK and MaxMeaK statistics of Puechmaille (2016) and Li and Liu (2018). The many *STRUCTURE* runs were required to reach the convergence necessary for accurate determination of the population structure in the GWAS (Yu et al., 2021).

### Genome-wide association studies

According to the criteria outlined by Yu et al. (2021), two general linear models (GLM), comprising PCA-only and Q-only, and four mixed linear models (MLM), including Q+D, Q+K, PCA+D and PCA+K implemented in TASSEL 5.0 (Bradbury et al., 2007), were tested in the marker-trait association studies, using the 6,213 SNP marker data and mean ID values of each of the eight RSA traits. For each model, significant markers associated with an RSA trait were determined only if the least amount of deviation from the expected −log_10_ *p*-value was observed in the quantile-quantile (Q-Q) plot. Manhattan plots were generated to represent the major marker-trait associations (MTAs). Significant SNP markers associated with the RSA traits were identified using the Bonferroni correction, i.e., *p*-value cut-off at 0.05/total number of markers (Benjamini and Hochberg, 1995). Stable MTAs detected by the different models and pleiotropic SNPs associated with the different RSA traits were considered credible.

### Identification of candidate genes

To identify candidate genes associated with significant SNP markers, the SNP sequences were used in BLASTN searches of *B. rapa* (AA), *B. oleracea* (CC), *B. napus* (AACC), and *Arabidopsis thaliana* genome assemblies in the EnsemblPlants (https://plants.ensembl.org/Multi/Tools/Blast) and National Centre for Biotechnology Information (https://blast.ncbi.nlm.nih.gov/Blast.cgi) databases. The physical locations of genes meeting an *E*-value ≤ 1e^-20^ and a percentage identity of ≥ 95% were mapped to the reference genomes.

## Results

### Phenotypic variation for RSA traits

The phenotypic variation in the eight RSA traits for the six *Brassica* species is presented in **Table 1**. Based on the measurements, the eight RSA traits were significantly different (*p*-value ≤ 0.05) among the *Brassica* genotypes tested. For example, TTRL ranged from 0.35 cm per plant for accession FG1063 (*B. juncea*) to 123.69 cm per plant for FG 643 (*B. oleracea*), while NTP ranged from 20 per plant for accession FG1063 (*B. juncea*) to 2,753 per plant for L345PC (a commercial canola cultivar) (**Table 1**). The coefficients of variation (%) for TTRL, NTP, TRSA, TLRL, TRL, BLL, TPRL, and RAD were 124.55, 92.26, 70.54, 70.13, 65.52, 62.01, 39.30, and 33.25, respectively (**Table 1**).

**Table 1:** Summary and phenotypic variation of eight root system architecture (RSA) traits in a collection of 379 *Brassica* genotypes representing *B. napus*, *B. oleracea*, *B. rapa*, *B. nigra*, *B. carinata*, and *B. juncea*.

RSA traits (per plant)	Abbreviation/Unit	Min	Max	Mean	SD	CV (%)	*p*-value
Total root length (TRL)	TRL/cm	18.46	414.51	137.51	90.10	65.52	<2e^-16^
Total surface area of roots (TRSA)	TRSA/cm^2^	2.32	60.11	16.86	11.89	70.54	<2e^-16^
Root average diameter (RAD)	RAD/cm	0.20	0.76	0.35	0.12	33.25	<2e^-16^
Number of tips (NTP)	NTP	20.00	2753.00	461.30	425.58	92.26	<2e^-16^
Total primary root length (TPRL)	TPRL/cm	8.77	46.73	24.92	9.79	39.30	<2e^-16^
Total lateral root length (TLRL)	TLRL/cm	9.34	249.93	83.40	58.49	70.13	<2e^-16^
Total tertiary root length (TTRL)	TTRL/cm	0.35	123.69	25.24	31.44	124.55	<2e^-16^
Basal link length (BLL)	BLL/cm	0.66	4.35	1.71	1.06	62.01	4.7e^-12^

Probability values (*p*-values) were generated through an ANOVA test of the 379 Brassica genotypes. *p*-value ≤ 0.05 indicates significant difference among genotypes.

The 379 *Brassica* genotypes in this study were divided into three groups based on their root sizes according to the criteria of Liu et al. (2021). Based on a median TRL value of 137.52 cm per plant ± 2 standard errors (SE) of 7.53, 177 accessions were classified as having large-sized roots (TRL >130.52 cm), 29 accessions were identified as having medium-sized roots (TRL ranging from 115.45 to 130.52 cm), and 173 genotypes were deemed as having small-sized root systems (TRL <115.45 cm) (**Supplementary Table 1**).

### Correlation analysis between selected RSA traits

**Figure 1:**
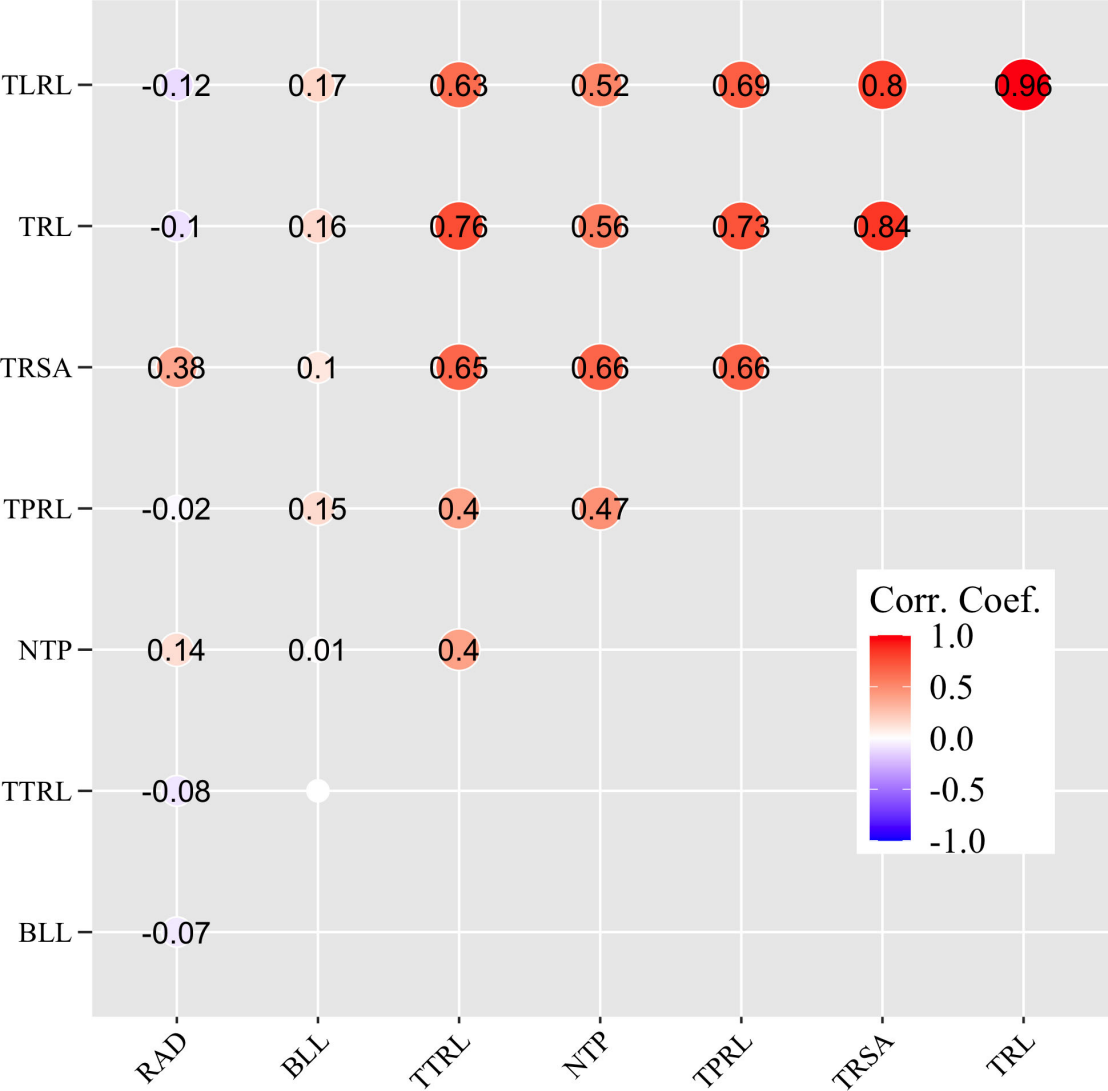
Correlation analysis between eight root system architecture traits as determined by a Spearman rank-based variable correlation test. Traits include total root length (TRL/cm), total surface area of roots (TRSA/cm^2^), root average diameter (RAD/cm), number of tips (NTP), total primary root length (TPRL/cm), total lateral root length (TLRL/cm), total tertiary root length (TTRL/cm), and basal link length (BLL/cm). ‘Corr. Coef.’ denotes the coefficient values of the correlation, with the strength of the correlation indicated in different colors. 0.0, no significant correlation (*p*-value > 0.05).

The Anderson-Darling test showed that all eight parameters were not normally distributed (*p*-value < 2.2e^-16^). A Spearman rank-based variable correlation test indicated that TRL, TRSA, and TLRL were highly and positively correlated with each other, with coefficient values ranging from 0.80 to 0.96 (*p*-value ≤ 0.05) (**Figure 1**). Relatively high positive correlations were also observed among TRL, TRSA, TLRL, TTRL, and NTP, with coefficients ranging from 0.52 to 0.76 (*p*-value ≤ 0.05), while moderate positive correlations existed among TPRL, TTRL, and NTP (coefficients ranging from 0.40 to 0.47, *p*-value ≤ 0.05) (**Figure 1**).

### Comparisons among the six *Brassica* species

**Figure 2:**
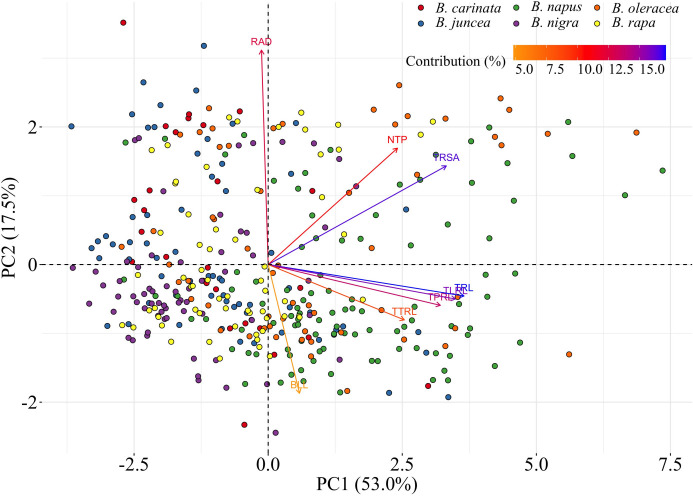
Principal components analysis (PCA) biplot among six *Brassica* species including *B*. *napus*, *B*. *oleracea*, *B*. *rapa*, *B*. *nigra*, *B*. *carinata*, and *B*. *juncea*. Traits include total root length (TRL/cm), total surface area of roots (TRSA/cm^2^), root average diameter (RAD/cm), number of tips (NTP), total primary root length (TPRL/cm), total lateral root length (TLRL/cm), total tertiary root length (TTRL/cm), and basal link length (BLL/cm). The different species are indicated by different colors. With the increase of PC1, most of the values of the root system architecture (RSA) traits increased, except for RAD. With the increase of PC2, RAD, NTP, and TRSA increased, while values of other traits decreased. Most of the *B*. *juncea*, *B*. *nigra*, *B*. *rapa*, and *B*. *carinata* genotypes are located in a cloud on the left side of the biplot, while most *B*. *napus* and *B*. *oleracea* genotypes are located on the right side of the biplot with relatively larger values for the root traits. This suggests that *B. napus* and *B*. *oleracea* have relatively larger root systems.

The PCA indicated that TRL, TRSA, TLRL, and TPRL accounted for 70.5% (PC1 = 53.0% and PC2 = 17.5%) of total genotypic variation for all RSA traits (**Supplementary Figure 1**). A biplot of the PCA indicated that TRL was the most important trait, followed by TRSA, TLRL, and TPRL (**Supplementary Figure 2**). The distribution of the six *Brassica* species based on the contribution coefficient of the eight traits is illustrated in the biplot of the PCA (**Figure 2**). *Brassica oleracea* showed the greatest variation in the eight RSA traits relative to the other species. As a result, the *B. oleracea* accessions were widely dispersed in the biplot of the PCA, although most were located on the right-hand side (**Figure 2**). Similarly, most of the *B. napus* accessions were located on the right-hand side of the biplot, indicating large RSA trait variations comparable with those observed in the *B. oleracea* accessions (**Figure 2**). Most of the *B. juncea*, *B. nigra*, *B. rapa*, and *B. carinata* accessions were located on the left-hand side of the PCA biplot (**Figure 2**). This suggests that *B. juncea*, *B. nigra*, *B. rapa*, and *B. carinata* have comparable RSA traits and complexity. Therefore, *B. napus*, and *B. oleracea* possessed the largest and most complex root systems among the six *Brassica* species.

**Figure 3:**
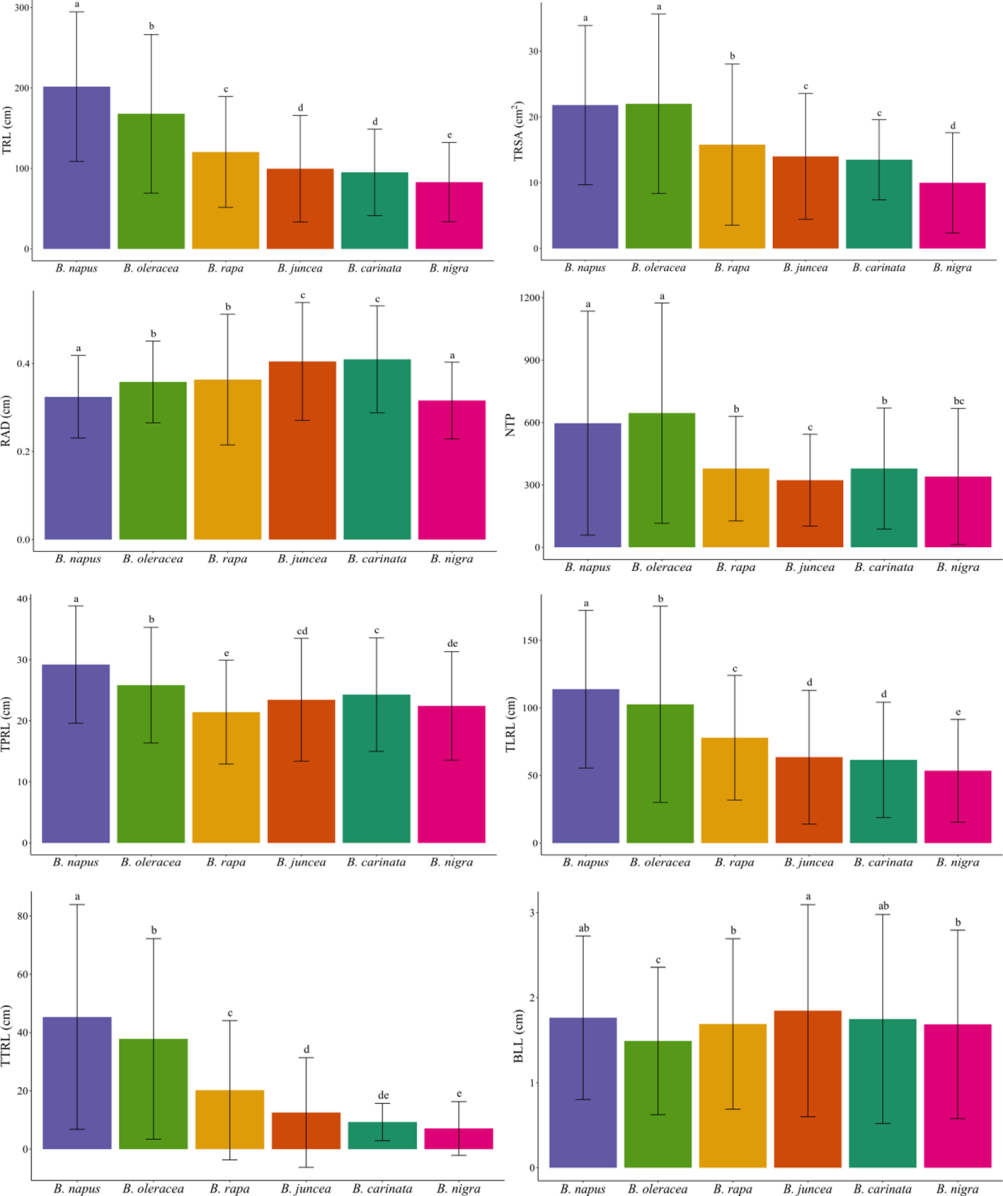
Bar plot of eight root system architecture traits among six *Brassica* species. *B*. *napus*, and *B*. *oleracea* had a relatively greater total root length (TRL/cm), total surface area of roots (TRSA/cm^2^), root average diameter (RAD/cm), number of tips (NTP), total primary root length (TPRL/cm), total lateral root length (TLRL/cm), and total tertiary root length (TTRL/cm), and basal link length (BLL/cm). In general, values for six of the eight traits (except for NTP and BLL) were lowest in *B*. *nigra*. Different letters indicate significant differences.

Duncan’s test of the eight RSA traits led to conclusions similar to the findings in the PCA biplot (**Table 2**). *Brassica napus* and *B. oleracea* exhibited relatively greater values of TRL, TRSA, NTP, TPRL, TLRL, and TTRL (**Table 2**, **Figure 3**), again indicating larger and more complex root systems than the other species. *Brassica juncea* and *B. carinata* did not show significant differences for seven out of eight parameters (except for NTP). In addition, *B. juncea* and *B. carinata* had the largest RAD compared with the other species (**Table 2**, **Figure 3**), indicating that their roots were thicker, possibly due to lower numbers of fine roots (root diameter < 0.2 cm). Among all species, *B. nigra* had the lowest values for six of the eight RSA traits examined (the only exceptions being NTP and BLL), suggesting that this species had the smallest root system. There were no significant differences observed for BLL in *B. juncea*, *B. napus*, *B. rapa*, *B. carinata*, or *B. nigra*, while this trait was lowest in *B. oleracea* (**Table 2**, **Figure 3**).

**Table 2:** Least square means of eight root system architecture traits among six *Brassica* species, *B. napus*, *B. oleracea*, *B. rapa*, *B. nigra*, *B. carinata*, and *B. juncea*, based on Duncan’s test.

Species	TRL	TRSA	RAD	NTP	TPRL	TLRL	TTRL	BLL
*B. juncea*	99.58^a^	14.00^a^	0.41^a^	322.93^a^	23.45^ad^	63.57^a^	9.29^a^	1.85^a^
*B. napus*	201.64^b^	21.80^b^	0.32^b^	596.84^b^	29.21^b^	113.81^b^	45.35^b^	1.76^ab^
*B. rapa*	120.43^c^	15.79^c^	0.36^c^	378.51^c^	21.43^c^	77.92^c^	20.21^c^	1.69^b^
*B. carinata*	95.07^a^	13.49^a^	0.41^a^	378.55^c^	24.30^d^	61.49^a^	9.29^ae^	1.75^ab^
*B. oleracea*	167.93^d^	22.00^b^	0.36^c^	645.14^b^	25.83^e^	102.64^d^	37.82^d^	1.49^c^
*B. nigra*	82.98^e^	9.99^d^	0.32^b^	340.12^ac^	22.45^ac^	53.45^e^	7.09^e^	1.69^b^

Traits examined included total root length (TRL/cm), total surface area of roots (TRSA/cm^2^), root average diameter (RAD/cm), number of tips (NTP), total primary root length (TPRL/cm), total lateral root length (TLRL/cm), total tertiary root length (TTRL/cm), and basal link length (BLL/cm).

Means followed by different letters in the same column are significantly different (p-value ≤ 0.05) from each other.

### Comparisons of RSA traits within the Six *Brassica* species

**Figure 4:**
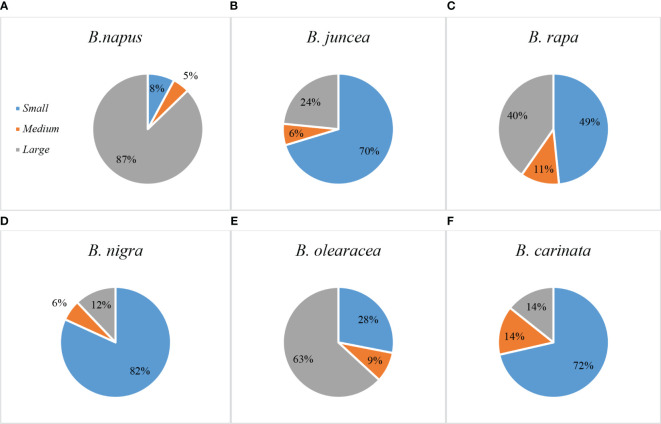
Percentage of genotypes with small, medium, or large sized root systems in six *Brassica* species – **(A)** *B*. *napus*, **(B)** *B*. *juncea*, **(C)** *B*. *rapa*, **(D)** *B*. *nigra*, **(E)** *B*. *oleracea*, and **(F)** *B*. *carinata*. Most *B. napus* and *B*. *oleracea* genotypes had larger-sized root systems, while most *B*. *juncea*, *B*. *rapa*, *B*. *nigra*, and *B*. *carinata* genotypes had smaller-sized root systems. Genotypes with medium-sized root system represented the smallest percentage for all the species. .

Based on TRL, the root sizes of the six *Brassica* species were of the order: *B. napus* (87% large, 5% medium, and 8% small) (**Figure 4A**) > *B. oleracea* (63% large, 9% medium, and 28% small) (**Figure 4E**) > *B. rapa* (40% large, 11% medium, and 49% small) (**Figure 4C**) > *B. juncea* (24% large, 6% medium, and 70% small) (**Figure 4B**) > *B. carinata* (14% large, 14% medium, and 72% small) (**Figure 4F**) > *B. nigra* (12% large, 6% medium, and 82% small) (**Figure 4D**). These results were consistent with results from PCA described above.

### SNP genome coverage and marker density

The plots of correlation coefficient (*r^2^*) and physical distance (in Mb) for SNP markers on chromosomes A and C are presented in **Supplementary Figure 6**. The mean number of filtered SNP markers was 510.3 ± 152.5 (range of 359 to 808) on the A-genome and 123.3 ± 34.0 (range of 88 to 190) on the C-genome. The filtered set of 5,103 and 1,110 markers covered 302,504.3 kilobases (kb) and 452,763.7 kb of the A- and C-genomes, respectively (**Table 3**). The mean inter-SNP marker distance or density for the A-genome was 62.9 ± 20.1 kb (range of 38.1 to 106.8), while for the C-genome it was 426.3 ± 106.9 kb (range 324.4 to 663.5) (**Table 3**). Thus, the marker density on the A-genome was about 7× higher than on the C-genome.

**Table 3:** Single nucleotide polymorphism (SNP) marker density and extent of intra-chromosomal linkage disequilibrium (LD) on A-, C- and AC- genome used in genome-wide association studies for the determination of root architecture traits.

Linkage group or Chromosome	Total # of SNP markers	# Filtered SNP markers	Length covered (kb)	Average inter-SNP marker distance (kb)	Pairwise comparisons of all linked SNP markers	Number (%) of SNP pairs in significant LD ^ϕ^	Average *r^2^*-value/chromosome	Estimated LD decay (kb) ^ψ^
A01	800	379	29044.5	76.6	17675	8203 (46.4%)	0.1456	599
A02	728	403	29846.5	74.1	18806	10438 (55.5%)	0.1712	920
A03	1458	808	37644.0	46.6	38868	19811 (51.0%)	0.1597	440
A04	909	498	22049.4	44.3	23394	12495 (53.4%)	0.1688	580
A05	916	498	29217.3	58.7	23625	12540 (53.1%)	0.1801	725
A06	1024	595	31714.7	53.3	28475	16609 (58.3%)	0.2002	600
A07	1298	722	27503.6	38.1	34825	17494 (50.2%)	0.1738	450
A08	633	359	21731.4	60.5	16675	9615 (57.7%)	0.1801	620
A09	757	404	43128.3	106.8	18925	10092 (53.3%)	0.1986	1400
A10	787	437	30624.6	70.1	20575	11452 (55.7%)	0.1876	580
C01	797	108	43764.1	405.2	4125	2754 (66.8%)	0.2284	9100
C02	820	113	54608.9	483.3	4375	2752 (62.9%)	0.2047	4000
C03	1598	190	61643.2	324.4	7740	4945 (63.9%)	0.2155	3100
C04	1224	168	55831.3	332.3	7125	4421 (62.0%)	0.1901	3160
C05	591	96	45327.5	472.2	3345	2188 (65.4%)	0.1996	4600
C06	874	103	44201.4	429.1	3601	2086 (57.9%)	0.1923	3185
C07	904	116	38338.5	330.5	4400	2785 (63.3%)	0.1981	2500
C08	755	128	50664.9	395.8	4668	3248 (69.6%)	0.2426	4600
C09	518	88	58383.8	663.5	3123	2295 (73.5%)	0.2461	8100
A-genome	9310	5103	302504.3	62.9 ± 20.1	251738	134687 (53.5%)	0.1762	691
C-genome	8072	1110	452763.7	426.3 ± 106.9	45441	27474 (60.5%)	0.2126	4705
AC-genome	17382	6213	755267.9	235.0 ± 200.1	224259	169182 (75.4%)	0.1830	2593

^*^ One thousand one hundred and fifty-four SNP markers located on scaffolds, or which could not be located were excluded from the analysis. ^ϕ^ The number and percentage of SNP pairs in significant LD were determined from Chi-squared tests at p-value ≤ 0.001.

^ψ^ The extent of LD decay was estimated from the projection of the intersection between the fitted curve of the data points and the 95^th^ percentile of unlinked r^2^-value threshold line (background LD) onto the physical distance axis.

### Estimation of linkage disequilibrium

The average values of *r* ^2^ and the schematic representation of decay for all chromosomes are presented in **Table 3**. Significant variation in the LD among chromosomes and between the A- and C-genomes was observed. The mean *r* ^2^-value was 0.1762 ± 0.0168 (range of 0.1456 to 0.2002) for the A-genome, and 0.2126 ± 0.0213 (range 0.1901 to 0.2461) for the C-genome (**Table 3**). The average *r* ^2^-value for the entire genome was 0.1830. Similarly, the estimated mean LD decay for the A-genome was 691.4 ± 283.6 kb (range of 440 to 1,400), while for the C-genome it was 4,705.0 ± 2,331.8 kb (range of 2,500 to 9,100); the mean LD decay was 2,592.6 ± 2,587.6 kb for the entire genome (**Table 3**). Thus, the LD decay for the A-genome was in the hundreds of kilobases, while it persisted for several thousands of kilobases for the C-genome.

### Population structure

**Figure 5:**
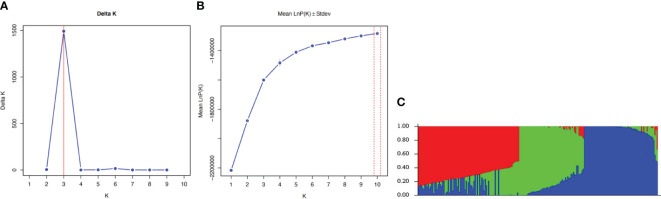
Bayesian cluster analysis of 313 *Brassica* accessions representing five species, including *B*. *napus*, *B*. *oleracea*, *B*. *rapa*, *B*. *carinata*, and *B*. *juncea*, estimated with STRUCTURE based on 6,213 SNP markers using 50,000 burn-in iterations and Markov Chain Monte Carlo (MCMC) lengths. The value of K determined following Evanno et al. (2005), and the population structure determined with the Puechmaille (2016) and Li and Liu (2018) alternative, indicated three clusters for all runs **(A, B)**. Detailed Bayesian clustering of the 313 accessions is shown in **(C)**, with each colour represents one ancestry component. The simplified view suggests three ancestral populations. The clusters were obtained based on the standard 70% threshold of similarity with STRUCTURE software (e.g., Yu et al., 2021). Genotypes not meeting this threshold were classified as admixtures or not belonging to any of the groups (Yu et al., 2021).

Population structure analyses were conducted to comprehend the population stratification for the GWAS. ΔK statistic values from STRUCTURE analyses runs with fewer than 10,000 burn-in iterations and 10,000 MCMC lengths suggested that the *Brassica* accessions could be grouped into three or six clusters, while runs with 20,000, 50,000, and 100,000 burn-in iterations and MCMC lengths consistently indicated three clusters. The population structure determined with the Puechmaille (2016) and Li and Liu (2018) alternative statistics (MedMedK, MedMeaK, MaxMedK and MaxMeaK) indicated three clusters for all *STRUCTURE* runs (**Figure 5**). Based on a threshold of 70%, all commercial cultivars and the *B. napus* accessions clustered in group 1, all *B. juncea* accessions clustered in group 2, and the *B. rapa* accessions (except ECD 05) clustered in group 3. The *B. oleracea* and *B. carinata* accessions were classified as admixtures, dispersed in clusters 1 and 2.

### Significant marker-RSA trait associations

**Figure 6:**
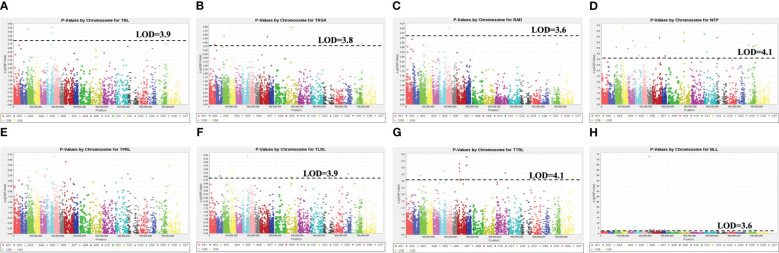
Manhattan plots of the PCA + K MLM models for identifying root system architecture traits loci in 313 *Brassica* accessions representing five species, including *B*. *napus*, *B*. *oleracea*, *B*. *rapa*, *B*. *carinata*, and *B*. *juncea*. Traits include **(A)** total root length (TRL/cm), **(B)** total surface area of roots (TRSA/cm^2^), **(C)** root average diameter (RAD/cm), **(D)** number of tips (NTP), **(E)** total primary root length (TPRL/cm), **(F)** total lateral root length (TLRL/cm), **(G)** total tertiary root length (TTRL/cm), and **(H)** basal link length (BLL/cm). The dashed horizontal lines indicate the Bonferroni-adjusted significance threshold (“logarithm-of-odds” (LOD) score). The dots above the significance threshold indicate single nucleotide polymorphisms (SNPs) associated with each trait.

The Quantile-Quantile plots for the eight RSA traits across the six models are presented in **Supplementary Figure 3**, while **Supplementary Figure 4** displays the individual plots for each trait. The PCA + K model showed the smallest deviation between the observed and expected values in the Q-Q plots. Therefore, the Manhattan plots for this model were used to identify the significant MTAs (**Figure 6**). Manhattan plots for all other models are presented in **Supplementary Figure 5**. Based on GWAS, 79 significant SNP markers were detected for the eight RSA traits. These comprised 6, 9, 26, 16, 12, and 6 SNP markers that were significantly associated with TRL, TRSA, RAD, TPRL, TTRL, and BLL, respectively (**Table 4**). Two markers, Bn_scaff_16414_1_p539478 and Bn_scaff_16514_1_p41089, were found to be co-associated with TPRL and TRL, while two other markers, Bn_A03_p5039586 and Bn_A06_p17049401, were co-associated with TTRL and TRL. No SNP markers were associated with NTP or TLRL. The number of significant markers and their chromosomal locations are presented in **Table 4**. Sixty-five of the MTAs were located on the A-genome, while 14 were located on the C-genome. The number of significant markers on chromosomes A01 to A10 was 6, 8, 9, 8, 2, 8, 5, 5, 12, and 2, respectively, while the number on chromosomes C01 to C09 (excluding chromosome C06) was 1, 3, 1, 1, 2, 1, 3, and 2, respectively (**Table 4**).

**Table 4:** Chromosomal location of significant single nucleotide polymorphism (SNP) markers associated with root system architecture traits in five *Brassica* species, including *B. napus*, *B. oleracea*, *B. rapa*, *B. carinata*, and *B. juncea*.

^θ^Model Used	Trait(s)	^α^SNP Marker	Marker Position		^β^Linkage Group	Description
			Start	End		
**PCA/Q+K/PCA+D/PCA+K**	TRSA	Bn_scaff_17036_1_p157245	243316	243334	A01	AFG1-like ATPase family protein
**PCA/Q+K/PCA+D/PCA+K**	TRSA	Bn_A03_p4123164	28467	28487	A03	ABC1 family protein
**PCA/Q+K/PCA+D/PCA+K**	TRSA	Bn_A03_p9765420	55863	55931	A03	Eukaryotic translation initiation factor 2 subunit 1
**PCA/Q+K/PCA+D/PCA+K**	TRSA	Bn_A09_p30678275	28355	28373	A09	AGC kinase 1.7
**PCA/Q+K/PCA+D/PCA+K**	TRSA	Bn_scaff_17566_1_p21523	541373	541673	C02	Calcium-dependent lipid-binding (CaLB domain) family protein
**PCA/Q+K/PCA+D/PCA+K**	TRSA	Bn_scaff_16445_1_p82664	105905	106136	C08	D-ribose-binding periplasmic protein
**PCA/PCA+D/PCA+K**	TRSA	Bn_A02_p5571981	1914669	1914789	A02	Histone deacetylase HDT2-like (LOC103851751)
**PCA/Q/Q+K/PCA+K**	RAD	Bn_A09_p1011107	2194	2259	C08	GDSL-like lipase/acylhydrolase superfamily protein
**PCA/Q/Q+K/PCA+D/PCA+K**	RAD	Bn_Scaffold000164_p174512	82582	82882	A01	Tetratricopeptide repeat (TPR)-like superfamily protein
**PCA**	RAD	Bn_A02_p5574727	1914669	1914789	A02	DNA repair protein Rad4 family
**PCA/Q/Q+D/Q+K/PCA+K**	RAD	Bn_A03_p19973423	1051672	1051972	A03	Zinc-dependent activator protein-1
**PCA/Q/Q+D**	RAD	Bn_A06_p21098677	976034	976334	A06	40S ribosomal protein S27
**PCA/Q+D**	RAD	Bn_A06_p26219274	4063	4263	A06	Root hair specific 17
**PCA/Q+K/PCA+K**	RAD	Bn_A07_p11698185	545848	546047	A07	Ribosomal protein L17 family protein
**PCA/PCA+D**	TPRL	Bn_A03_p7058001	11959	11976	A03	GDSL-like lipase/acylhydrolase superfamily protein
**PCA/PCA+D/PCA+K**	TPRL	Bn_A04_p7442353	327569	327586	A04	Transferases, transferring acyl groups
**PCA/PCA+D/PCA+K**	TPRL	Bn_A04_p7442886	1537209	1537329	A04	Transmembrane protein
**PCA/PCA+D/PCA+K**	TPRL	Bn_A04_p7443395	1317835	1317852	A04	Hydroxymethylglutaryl-CoA synthase/HMG-CoA synthase
**PCA/PCA+D/PCA+K**	TPRL	Bn_A05_p19554281	1256900	1257100	A05	tRNAse Z4
**PCA/Q+K/PCA+D/PCA+K**	TPRL	Bn_A06_p2619089	972332	972452	A06	Exocyst subunit exo70 family protein
**PCA/PCA+D/PCA+K**	TPRL	Bn_A07_p3921656	174533	174552	A07	Nucleotide-sugar transporter family protein
**PCA/Q+K/PCA+D/PCA+K**	TPRL	Bn_A09_p7560188	192457	192577	A09	Exocyst complex component sec3A
**PCA/PCA+D/PCA+K**	TPRL/TRL	Bn_scaff_16414_1_p539478	559675	559847	C05	Protein sulfur deficiency-induced 2-like
**PCA/PCA+K**	TPRL/TRL	Bn_scaff_16514_1_p41089	1732209	1732509	C07	Serine/threonine-protein kinase STY13-like
**PCA/Q+K/PCA+D/PCA+K**	TPRL	Bn_scaff_17487_1_p1782181	305799	305999	C09	Hydroxyproline-rich glycoprotein family protein
**Q/Q+D/Q+K/PCA+K**	RAD	Bn_A04_p5183306	493479	493599	A04	Uncharacterized
**Q**	RAD	Bn_A08_p20968239	565906	566106	A08	Phospholipase A1-IIalpha
**Q+D/Q+K/PCA+K**	RAD	Bn_A01_p6482543	947870	947909	A01	Homeodomain-like superfamily protein
**Q+D**	RAD	Bn_A02_p5516551	260256	260376	A02	Cytochrome P450, family 735, subfamily A, polypeptide 2
**Q+D**	RAD	Bn_A02_p15693192	28725	28807	A02	Transducin/WD40 repeat-like superfamily protein
**Q+D**	RAD	Bn_A06_p5280225	62713	62741	A04	Phosphoglycolate phosphatase
**Q+D**	RAD	Bn_scaff_23293_1_p25406	376195	376495	A09	NAC domain containing protein 35
**Q+D**	RAD	Bn_A01_p7942548	394003	394162	C01	DNA repair protein Rad4 family
**Q+K**	TRSA	Bn_A08_p12599446	55394	55594	A08	Methylcrotonoyl-CoA carboxylase beta chain, mitochondrial
**Q+K**	TRSA	Bn_Scaffold000172_p99636	93776	93813	A05	ARM repeat superfamily protein
**Q+K**	TPRL	Bn_A02_p10126530	346076	346196	A02	Plant self-incompatibility protein S1 family
**Q+K**	TPRL	Bn_A09_p24564546	4286	4302	A09	*Brassica napus* genome assembly, chromosome: A09
**Q+K/PCA+K**	TTRL/TRL	Bn_A03_p5039586	3568080	3568097	A03	Temperature-induced lipocalin-1
**Q+K**	TTRL	Bn_A03_p6744274	429097	429121	A03	Putative defensin-like protein 225
**Q+K**	TTRL	Bn_A03_p19974784	1053033	1053333	A03	Histidine kinase 2
**Q+K/PCA+K**	TTRL/TRL	Bn_A06_p17049401	43512	43640	A06	Receptor-like kinase TMK2
**Q+K**	TTRL	Bn_scaff_18100_1_p593993	299324	299431	A09	Malate dehydrogenase 1, cytoplasmic
**Q+K**	TTRL	Bn_A09_p13729175	142766	142782	A09	Disease resistance protein TAO1
**Q+K**	TTRL	Bn_A09_p16833397	67051	67275	A09	Protein enhanced disease resistance 2-like
**Q+K**	TTRL	Bn_A09_p19872952	41038	41054	A09	Alcohol dehydrogenase-like 3
**Q+K**	TTRL	Bn_A09_p19865476	743365	743382	A09	DEA(D/H)-box RNA helicase family protein
**Q+K**	TTRL	Bn_A09_p33660289	7754	7954	A09	CDP-diacylglycerol–serine O-phosphatidyltransferase 1
**Q+K**	TTRL	Bn_A10_p4624712	1352963	1353083	A10	Equilibrative nucleoside transporter 7
**Q+K**	TTRL	Bn_A10_p4622209	34449	34569	A10	S-adenosyl-L-methionine-dependent methyltransferases superfamily protein
**Q+K**	TTRL	Bn_scaff_17440_1_p268977	101613	101813	C03	Phosphatidylinositol 4-phosphate 5-kinase MSS4-like protein
**Q+K**	TTRL	Bn_scaff_22481_1_p200007	624510	624630	C09	Haloacid dehalogenase-like hydrolase (HAD) superfamily protein
**PCA+D**	RAD	Bn_A01_p5335218	66516	66534	A01	Abscisic acid 8’-hydroxylase 1
**PCA+D**	RAD	Bn_A01_p10945930	11035	11052	A01	LRR and NB-ARC domains-containing disease resistance protein
**PCA+D**	RAD	Bn_A02_p10781906	44741	44765	A02	Lariat debranching enzyme
**PCA+D**	RAD	Bn_A02_p19704677	2839848	2839865	A02	U-box domain-containing protein 37
**PCA+D**	RAD	Bn_A04_p16313477	354946	355064	A04	Ribosomal S17 family protein
**PCA+D**	RAD	Bn_A04_p18562244	188942	189242	A04	4-hydroxy-tetrahydrodipicolinate synthase 2, chloroplastic
**PCA+D**	RAD	Bn_A06_p24156940	360125	360145	A06	Disease resistance protein (TIR-NBS-LRR class) family
**PCA+D**	RAD	Bn_A10_p12072657	43451	43632	A07	Ubiquitin family protein
**PCA+D**	RAD	Bn_A07_p6501207	9193	9381	A07	GDSL-like lipase/acylhydrolase superfamily protein
**PCA+D**	RAD	Bn_A02_p709952	1015827	1015844	A07	Peroxidase superfamily protein
**PCA+D**	RAD	Bn_A09_p14282683	37874	38166	A09	Uncharacterized
**PCA+D**	TPRL	Bn_A03_p7110332	1001573	1001590	A03	Transducin family protein/WD-40 repeat family protein
**PCA+D**	TPRL	Bn_A08_p20546110	342771	342891	A08	Myosin-binding protein 1
**PCA+D**	TPRL	Bn_scaff_17807_1_p98331	103262	103462	C02	Leaf rust 10 disease-resistance locus receptor-like protein kinase-like 2.7
**PCA+D**	TPRL	Bn_scaff_16759_1_p264813	1114468	1114528	C04	Chloride channel D
**PCA+D**	TPRL	Bn_C13729753_p243	858575	858775	C05	Integrase-type DNA-binding superfamily protein
**PCA+K**	TRL	Bn_A06_p17452087	979492	979792	A06	Polynucleotidyl transferase
**PCA+K**	TRL	Bn_A06_p17176086	709607	709869	A06	TCV-interacting protein
**PCA+K**	TRL	Bn_A01_p22999151	637603	637625	A01	SsrA-binding protein
**PCA+K**	TRL	Bn_scaff_17522_1_p1724143	2404	2524	A02	Tetratricopeptide repeat (TPR)-like superfamily protein
**PCA+K**	TRL	Bn_scaff_21861_1_p33827	717915	718115	C02	Cytochrome P450, family 72, subfamily A, polypeptide 11
**PCA+K**	TRL	Bn_scaff_16445_1_p894350	245816	246116	C08	Cytochrome P450, family 87, subfamily A, polypeptide 2
**PCA+K**	BLL	Bn_A03_p7178917	1429677	1429797	A03	Cysteine-rich RLK (receptor-like protein kinase) 27
**PCA+K**	BLL	Bn_scaff_26139_1_p313572	539325	539438	A04	Inosine triphosphate pyrophosphatase family protein
**PCA+K**	BLL	Bn_A06_p3839293	53144	53444	A06	Phosphatidylinositol 4-phosphate 5-kinase 7
**PCA+K**	BLL	Bn_scaff_17821_1_p119310	541935	542135	A08	V-type proton ATPase subunit c’’2
**PCA+K**	BLL	Bn_A08_p16632230	489602	489630	A08	*Brassica napus* genome assembly
**PCA+K**	BLL	Bn_A09_p9101925	533420	533437	A09	SNARE associated Golgi protein family

^θ^Mixed Linear Model (MLM) designations: PCA, principal component analysis; Q, population structure; K, Kinship. ^α^SNP markers denoted with the same superscript letter mapped to multiple chromosomes on the reference genomes. The type of PCR-based markers showing trait association has been specified. ^β^Linkage groups A1-A10 = *B. rapa, B. napus, B. juncea*, and C1-C9 = *B. oleracea*, *B. napus*, *B. carinata*. Putative functions are based on matching entries in the EnsemblPlants and NCBI GenBank databases.

### Functions of proteins encoded by significant sequences

The identified sequences encoded proteins associated with functions involved in various cellular and biochemical processes, including ATP binding, lipid binding, ribosome binding, DNA binding, mRNA binding, RNA binding, metal ion binding, ATPase activity, ATP hydrolysis activity, kinase activity, lipase activity, transferase activity, transcription and translation factor activity, substrate selectivity, catalytic activity, and carbohydrate metabolism (**Table 4**). More importantly, other proteins were associated with cell wall synthesis, cell growth, organ morphogenesis, transmembrane transporter activity, sugar-phosphatase activity, and vesicle fusion. These processes are related to fundamental biological and physiological mechanisms involved in root growth and development. Some other sequences encoded stress tolerance and disease resistance proteins, such as NAC domain containing protein 35, ARM repeat superfamily protein, DEA(D/H)-box RNA helicase family protein, and LRR and NB-ARC domains. Proteins of unknown molecular function were also detected (**Table 4**).

## Discussion

Root system architectural traits are critical to the plant’s ability to absorb water and nutrients from the soil (Zobel et al., 2007; Lynch, 2019; Wen et al., 2019; Liu et al., 2021; Sun et al., 2021). The response of crops to abiotic stresses is influenced by their RSA. Since roots grow underground, they serve as the first line of defense in detecting stress signals and adapting their genetic program for post-embryonic growth to cope effectively with these challenges (Lynch, 1995). Morphological variations often correspond to physiological or functional variations (de Dorlodot et al., 2007). In this study, a highly positive correlation was observed for seven of the eight RSA traits (except RAD) evaluated in the 379 *Brassica* genotypes. Ibrahim et al. (2021) also reported a positive correlation between RSA traits in a study of 388 *B. napus* accessions. The mean BLL values were similar across all six species examined, indicating that the potential growth of the roots of these species is quite similar, probably due to the phenotyping platform.

Root architectural traits can serve as a focal point for enhancement and optimization, tailored to meet the specific requirements dictated by soil conditions (Arifuzzaman and Rahman, 2017). For example, Thomas et al. (2016) examined the root structure of various growth types of *B. napus* and determined that root morphology has the potential to enhance crop yield, provided that appropriate genetic markers associated with agronomic traits can be identified (Arifuzzaman and Rahman, 2017). The findings from this study revealed that root traits dynamics were significantly influenced by the genotype and species, highlighting the significant role of genetic factors in RSA. The presence of shared QTL between root characteristics and productivity measures such as yield, water usage, or nutrient acquisition suggests that the former contributes to determining the latter in numerous instances (Steele et al., 2006; Ibrahim et al., 2021). One example is the utilization of a QTL termed *DRO1*, which governs both root growth angle and root depth in rice, for improving the root traits of an Indian upland rice variety through marker-assisted selection to enhance water efficiency (Steele et al., 2006; Ibrahim et al., 2021). In the case of *Brassica* crops, additional studies regarding the relationship between root-related traits and productivity or nutrient and water use efficiency are needed.

From a functional perspective, a root tip is the primary site for nutrient and water uptake in a plant. In addition, root tips produce exudates that attract significantly higher levels of microorganisms to the rhizosphere surrounding the root tips compared with the bulk soil located a few millimeters away (Curlango-Rivera and Hawes, 2011). Furthermore, individual root tips that develop in soil provide strong anchorage, which facilitates deeper penetration of roots into hard soil niches or areas (Bengough et al., 2016). In this study, significantly greater NTP per plant in *B. napus* and *B. oleracea* accessions suggests they possess the potential for stronger soil anchorage, as well as better nutrient and water uptake capabilities. Longer primary roots (Wasson et al., 2012), a larger root diameter (Uga et al., 2013), and abundant and steeper lateral roots (Lynch, 2013) were reported to lead to a deeper and more resilient root system with increased radial hydraulic conductivity at depth and decreased metabolic costs for drought adaptation (Khan et al., 2016). The aforementioned root traits were also reported to increase the efficiency of exudation of organic anions (Lynch, 2015) and enhance interactions with microbes (Walch-Liu et al., 2006), resulting in a high tolerance to deficiencies in nutrients such as nitrogen and phosphate (Khan et al., 2016). In the current study, the *B. napus* and *B. oleracea* accessions that possessed relatively larger root systems with larger root surface area, and longer and more vigorous roots, would be expected to provide good anchorage and penetration into the soil. This suggests high developmental plasticity in case of drought or nutrient deficiency (Choi and Cho, 2019). Akhatar and Banga (2015) reported a positive association between seed yield and root length in *B. juncea* under irrigated conditions. However, negative correlations between root length, root surface area, and root mass with yield under drought stress have been observed in potatoes (*Solanum tuberosum*) (Zarzyńska et al., 2017). Hammond et al. (2009) and Pongrac et al. (2020) reported that genotypes of *B. oleracea* with high phosphorus absorption efficiency were characterized by more and longer lateral roots, and had significantly increased yields independent of external phosphorus concentration. Thus, the *B. oleracea* genotypes with large TLRL identified in this study will have the potential for high phosphorus absorption efficiency, and likely have the potential for higher yields. However, the efficiency of nutrient use is complex and influenced by physiological traits specific to each species and other factors (Pongrac et al., 2020).

The distribution of roots, especially those with strong and thick root systems capable of deeper penetration in the soil, is pivotal in influencing a plant’s capacity to acquire essential resources like water (Fenta et al., 2014). Compared with other species, *B. juncea* and *B. carinata*, with the largest RAD, might be better at surviving in dry and compacted soil given their relatively thick and stronger roots. Similar patterns have been identified in other crop species. For example, rice (*Oryza sativa* L.) varieties with deeper and thicker root systems exhibited increased yields and quality under drought conditions (Suji et al., 2012). Similar trends were also observed in four common bean (*Phaseolus vulgaris* L.) cultivars, wherein varieties with deeper and stronger roots exhibited enhanced yield, growth, reduced canopy temperature, and decreased soil moisture extraction; in poor soil, however, root variations were not evident (Sponchiado et al., 1989; Lopes et al., 2011).

Salinity is an abiotic stress with detrimental effects on agricultural productivity and sustainability, making it a significant global concern (Shahid et al., 2018). Approximately 830 million ha are projected to be affected by salinity (Szabolcs, 1989), which is still increasing every year (Qadir et al., 2014; Minhas et al., 2020). Agricultural productivity under saline conditions is low, posing a perpetual risk of crop failure. This underscores the significance of enhancing crops to thrive in such environments (Minhas et al., 2020). Reducing main root elongation limits the transport of sodium ions from roots to shoots in saline soils (Rus et al., 2006; Munns and Tester, 2008; Katori et al., 2010). In addition, the storage of sodium ions in root vacuoles and steles can increase the efficiency of water extraction and ion exclusion for salinity tolerance (Gupta and Huang, 2014; Khan et al., 2016). The current study suggests that *B. nigra* and *B. rapa*, which had relatively shorter primary roots compared with the other four species, may exhibit greater tolerance to salinity.

Soilborne pathogens also represent a major threat to crop production. Clubroot, caused by *Plasmodiophora brassicae*, is a particularly important disease of *Brassica* species (Strelkov and Hwang, 2014). In general, Fredua-Agyeman et al. (2019) found that the order of clubroot resistance, from greatest to smallest, was *B. nigra* > *B. oleracea* > *B. rapa* > *B. napus*. As such, while *B. nigra* had the smallest root system in this study, this species also appears to have the strongest resistance to clubroot. A similar association was noted between root size and Verticillium stripe (*Verticillium longisporum*) severity (Cui et al., 2023). Inoculation of 3-week-old canola plants resulted in more severe disease compared with inoculation of 1- or 2-week-old plants, which had smaller root systems. Furthermore, *B. nigra*, *B. carinata*, and *B. juncea*, characterized by relatively smaller and less intricate root systems in the present study, also exhibited greater resistance to Sclerotinia stem rot (*Sclerotinia sclerotiorum*) (Navabi et al., 2010; Neik et al., 2017) and blackleg (*Leptosphaeria maculans* and *L. biglobosa*) (Roy, 1984; Sjödin and Glimelius, 1988; Rimmer and van den Berg, 1992; Li et al., 2015; You et al., 2016; Neik et al., 2017). Infection by soilborne pathogens can destroy the roots, reduce root density, and diminish the functional effectiveness of the surviving infected roots (Román-Avilés et al., 2004). It is likely that host genotypes with smaller root systems offer fewer opportunities for pathogen invasion, given the smaller surface area and reduced opportunity for contact. As such, the greater resistance may be due to disease escape during the early growth stages of the plant. In the GWAS, 6,213 SNP markers were used to measure RSA traits, including 5,103 A-genome and 1,110 C-genome markers. Comparative genomic studies on *Brassica* genomes have reported that 1 centimorgan (cM) on a genetic map corresponds to ~500 kb (Suwabe et al., 2006; Ecke et al., 2010; Delourme et al., 2013). Therefore, the 302.5 Mb marker coverage estimated in this study for the A-genome and 452.8 Mb for the C-genome corresponded to ~605 cM and ~905 cM, respectively. As such, the 6,213 SNP markers covered a total of ~1,510 cM, which represents ~60% of the estimated 2,500 cM *B. napus* genome. The determined genome coverage was comparable with a value of ~645 Mb obtained in studies using a *Brassica* 60K array (Qian et al., 2014; Qu et al., 2017) and specific-locus amplified fragment sequencing technology (Zhou et al., 2017). In comparison, the filtered set of 6,213 markers on the *Brassica* 19K SNP array provided about 3× more coverage than the *Brassica* 13.2K SNP array from the same company used in a previous study (Fredua-Agyeman et al., 2020).

The mean marker density using the *Brassica* 19K SNP array was 62.9 ± 20.1 (8.43 SNP markers/cM) on the A-genome, 426.3 ± 106.9 (1.22 SNP markers/cM) on the C-genome, and 235.0 ± 200.1 (4.1 SNP markers/cM) on the entire genome. In comparison, the mean marker density using the *Brassica* 13.2K SNP array was 63.4 ± 21.9 (8.46 SNP markers/cM) for the A-genome, 15.0 ± 8.4 (44.3 SNP markers/cM) for the C-genome, and 40.5 ± 29.8 (11.8 SNP markers/cM) for the entire *B. napus* genome (Fredua-Agyeman et al., 2020). Thus, the marker density remained consistent for the A-genome, but it was 2–3× lower on the C-genome when genotyping was conducted with the *Brassica* 19K vs. 13.2K SNP array. This discrepancy was anticipated, as the 1,110 filtered set of SNP markers on the C-genome was distributed over 302.5 Mb or 905.5 cM on the 19K array, in contrast to 2,367 markers on the C-genome spread over 26.7 Mb or 53.4 cM on the 13.2K array.

Linkage disequilibrium, which is the non-random association between alleles at different loci, determines the power and precision of association mapping studies using molecular markers and unobserved QTL (Goddard and Hayes, 2009; Qu et al., 2020). Determination of the extent of LD is essential for making inferences regarding the genetic forces shaping a population (Qanbari, 2019). The extent of LD reported by Fredua-Agyeman et al. (2020) using the *Brassica* 13.2K SNP array varied from 1,100 to 2,300 kb for the A-genome and from 200 to 1,500 kb for the C-genome. In the present study using the *Brassica* 19K SNP array, LD varied from 440 to 1,400 kb for the A-genome and from 2,500 to 9,100 kb for the C-genome. The difference in LD values obtained with the two *Brassica* arrays could reflect the different marker densities. The low marker density on the C-genome might be responsible for the extended ranges of the LD decay. However, the LD values for the A- and C-genomes were consistent with those reported in other studies (Wu et al., 2016; Qu et al., 2017; Zhou et al., 2017). Based on the minimum LD decay (440 kb or 0.88 cM), a minimum of 3,200 markers were needed to perform the GWAS. Therefore, the 6,213 SNP markers in this study represented approximately twice the number needed to perform the analysis.

The MTAs analyses identified three genomic regions on chromosomes A02, A03, and A06 that were associated with RSA traits. In the case of the A02 chromosome, the SNP marker Bn_A02_p5571981 overlapped with a histone deacetylase HDT2-like protein. This protein negatively regulates GIBBERELLIN 2-OXIDASE2 (GA2ox2) expression, which determines cell number in the Arabidopsis root meristem and elongation zone (Li et al., 2017). The increased expression of GA2ox2 in HDT1/2 was reported to cause a decrease in gibberellin (GAs) levels, leading to an earlier transition from cell division to the expansion phase of transit-amplifying cells (Li et al., 2017). On chromosome A03, a histidine kinase 2 (AHK2) encoded by genes that overlap SNP marker Bn_A03_p19974784 can positively regulate the level of cytokinin, which negatively regulates root development in Arabidopsis (Nishimura et al., 2004; Riefler et al., 2006). In Arabidopsis, histidine kinase homologs function as receptors for cytokinin and play an overlapping role in regulating the growth of shoots and roots (Nishimura et al., 2004). Root hair specific 17, encoded by genes associated with SNP marker Bn_A06_p26219274 on chromosome A06, is an expressed protein controlling root hair cell expression for regulating the root growth of Arabidopsis (Won et al., 2009).

This study characterized the RSA traits of the six most economically important *Brassica* species. It represents the most comprehensive study of its kind, where the root traits of such a large number of *Brassica* accessions have been examined. This broader scope enabled a deeper exploration of the genetic basis underlying root development, and its implications for crop improvement strategies. However, this study was conducted in a hydroponic system, which does not fully reflect root growth in soil environments. Therefore, further studies will focus on cultivating a subset of identified accessions with small, medium, and large root sizes in soil to validate the findings. Additionally, accurate mapping of genomic regions associated with RSA traits in *Brassica* species is necessary. To achieve this, bi-parental mapping populations (F_2_ and doubled haploid) will be developed through crosses between selected large and small root-sized accessions to precisely map these genomic regions. Despite these limitations, this study, which encompassed a wide range of *Brassica* species and root traits, has laid the groundwork for future investigations aimed at breeding programs tailored to enhance root traits and stress tolerance in *Brassica* crops.

## Conclusion

Overall, this study demonstrated that significant genetic variation exists in the RSA of different *Brassica* species under controlled environmental conditions. The results also indicated correlations between specific RSA traits, with TRL, TRSA, TPRL, and TLRL exhibiting the strongest associations. These identified RSA traits can serve as valuable indicators for further investigations into stress tolerance under field conditions or abiotic stress scenarios. GWAS identified significant MTAs associated with proteins involved in cell wall synthesis, cell growth, and organ morphogenesis, as well as various proteins associated with cellular, biological, and physiological processes involved in root growth and development. The candidate genes related to root growth were located on chromosomes A02, A03, and A06 of the *B. rapa* genome. We identified *B. napus*, *B. juncea*, *B. rapa*, *B. nigra*, *B. oleracea*, and *B. carinata* accessions with variable RSA traits. These accessions hold promise for breeding *Brassica* crops suitable for different environments.

## Data availability statement

The datasets presented in this study can be found in online repositories. The names of the repository/repositories and accession number(s) can be found in the article/**Supplementary Material**.

## Author contributions

CY: Conceptualization, Formal analysis, Investigation, Methodology, Software, Validation, Visualization, Writing – original draft. RF: Conceptualization, Formal analysis, Methodology, Resources, Software, Supervision, Writing – review & editing. SH: Conceptualization, Data curation, Funding acquisition, Project administration, Resources, Supervision, Writing – review & editing. LG: Methodology, Resources, Software, Writing – review & editing. SS: Conceptualization, Data curation, Funding acquisition, Project administration, Resources, Supervision, Writing – review & editing.
