# Supplementary material for: Genome-wide association studies of root system architecture traits in a broad collection of Brassica genotypes
Source: Front Plant Sci. 2024 May 28;15:1389082. doi: 10.3389/fpls.2024.1389082 (PMC11165082; doi:10.3389/fpls.2024.1389082)
Supplement: Supplementary file 1 [file DataSheet_1.docx]

Supplementary Material

# **Supplementary Figures**


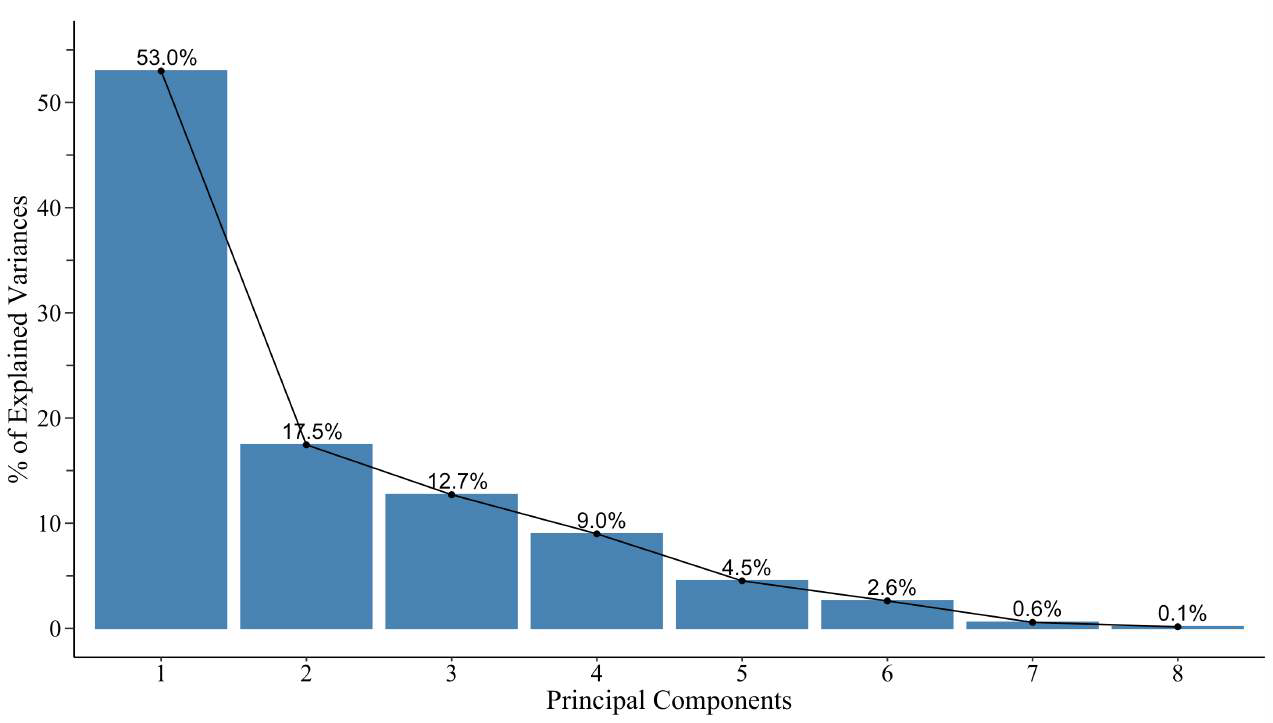


**Supplementary Figure 1.** Scree plot explaining cumulative variance of the principal components. PC 1 and PC 2 explained 53% and 17.5% of the cumulative variance in the dataset, respectively, which accounted for 70.5% of total genotypic variation among all root system architecture (RSA) traits. Traits include total root length (TRL/cm), total surface area of roots (TRSA/cm^2^), root average diameter (RAD/cm), number of tips (NTP), total primary root length (TPRL/cm), total lateral root length (TLRL/cm), total tertiary root length (TTRL/cm), and basal link length (BLL/cm).


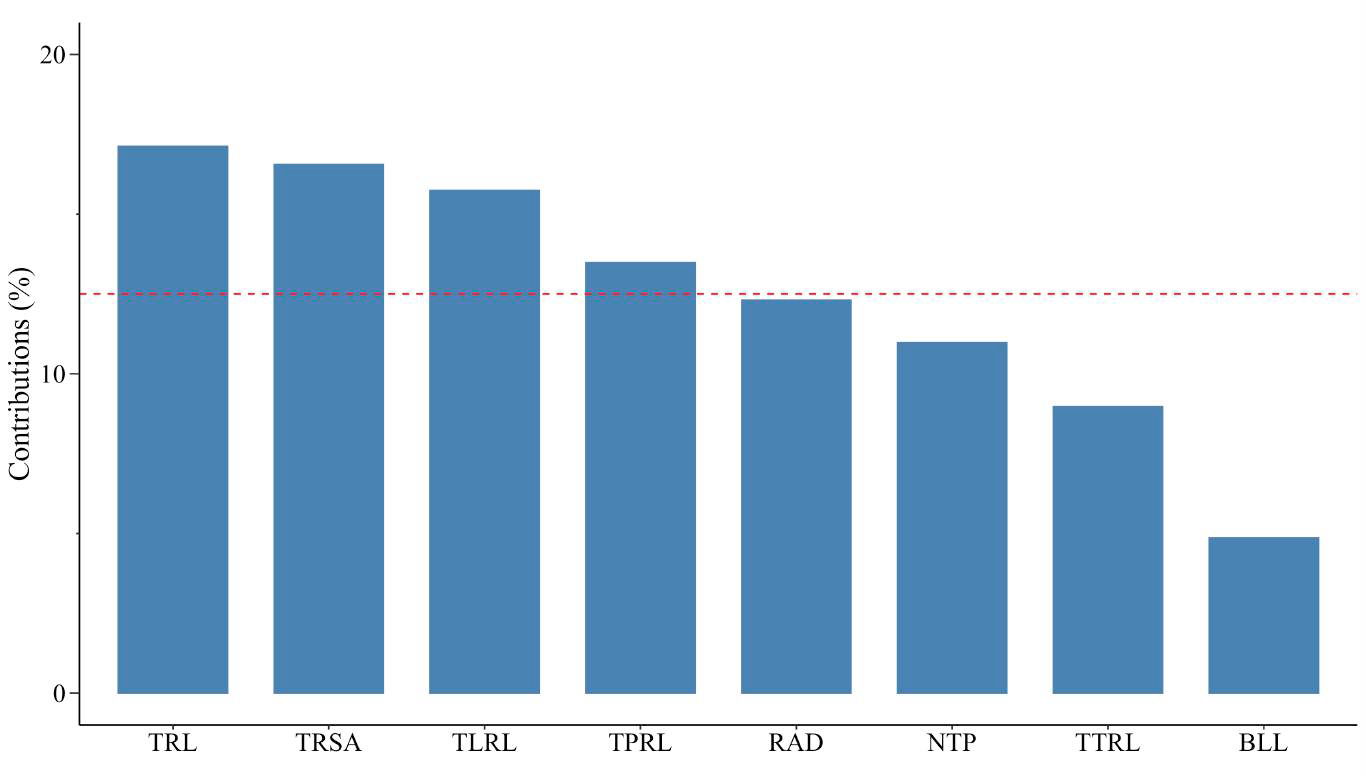


**Supplementary Figure 2.** Scree plot for all variables of principal components 1 and 2. The red line indicates the average contribution. Total surface area of roots (TRSA/cm^2^), total root length (TRL/cm), total lateral root length (TLRL/cm), and total primary root length (TPRL/cm) made the greatest contributions, followed by root average diameter (RAD/cm), number of tips (NTP), total tertiary root length (TTRL/cm), and basal link length (BLL/cm).


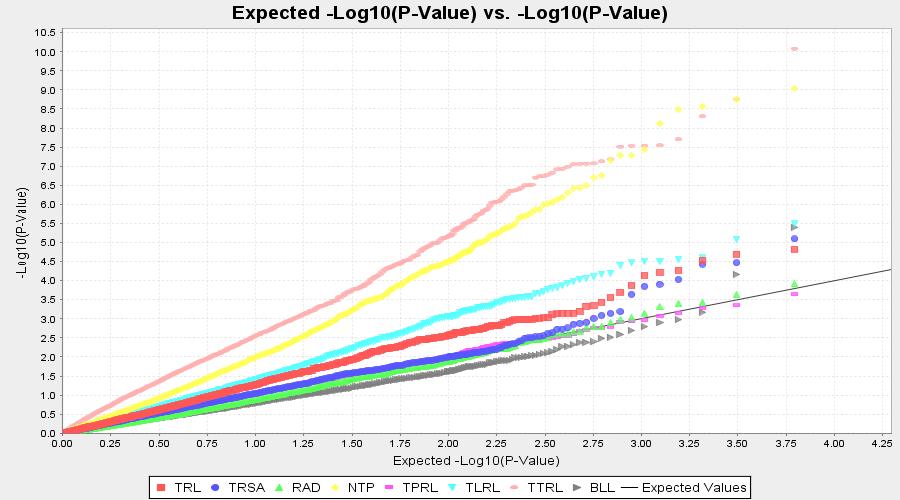

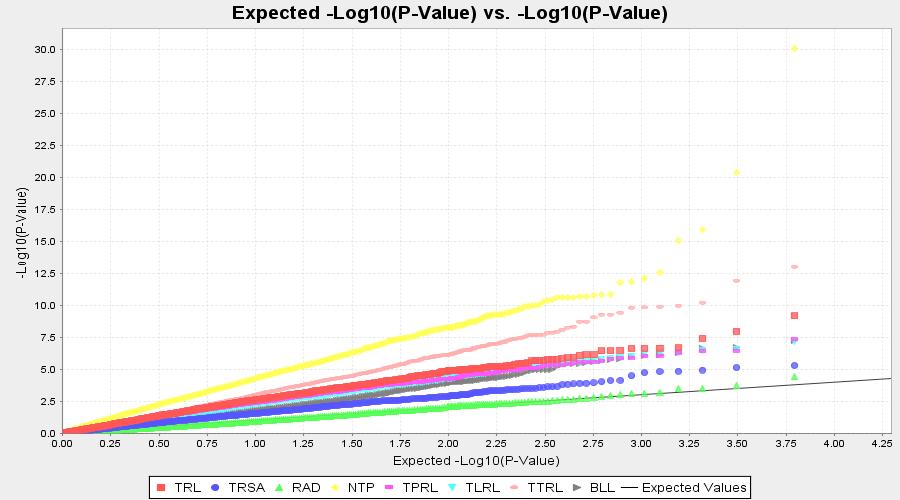

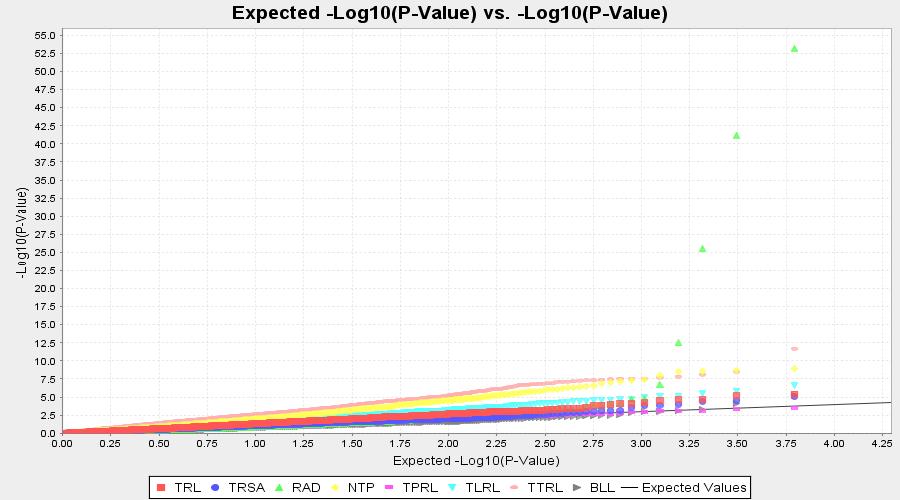


1. **(c) (e)**

**
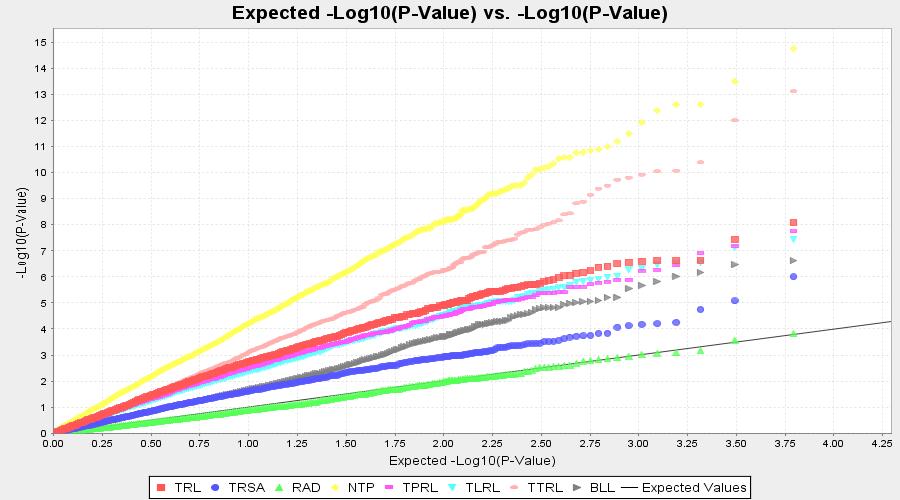

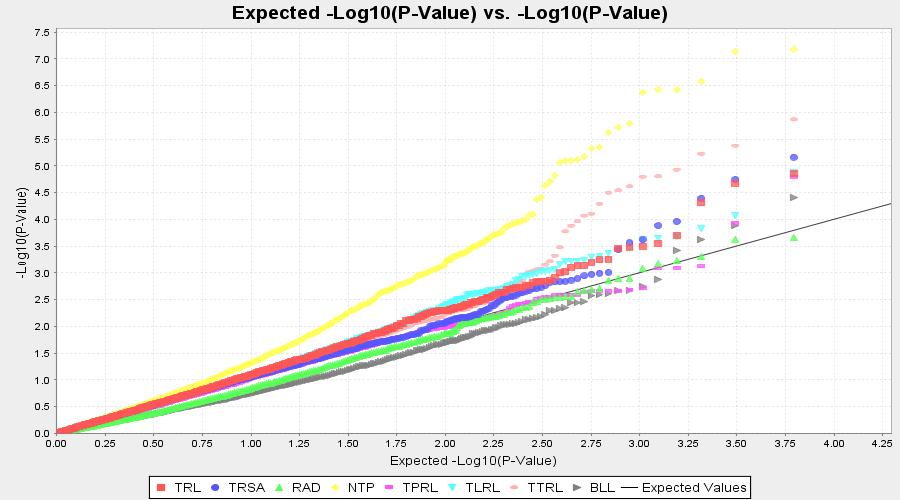

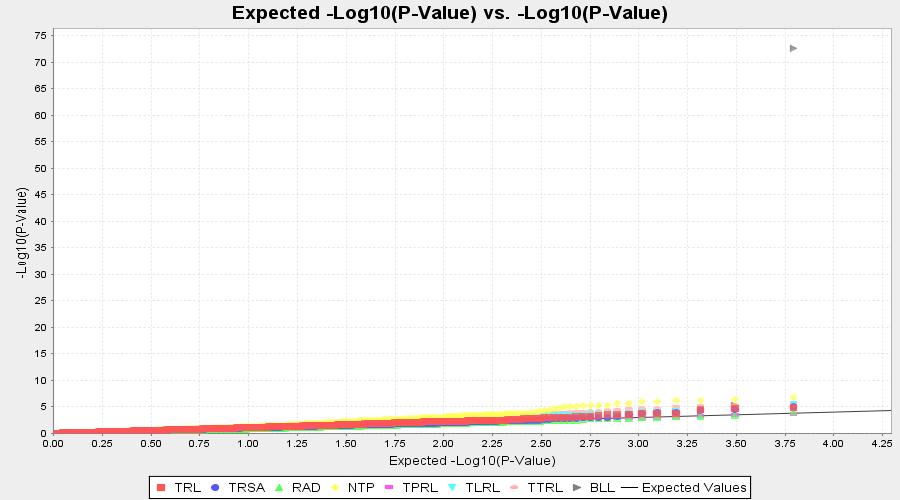
**

**(b) (d) (f)**

**Supplementary Figure 3.** Quantile-Quantile comparison of six GWAS models for identifying loci associated with root architecture (RSA) traits in 313 *Brassica* accessions representing five species, including *B. napus*, *B. oleracea*, *B. rapa*, *B. carinata*, and *B. juncea.* The two general linear models (GLM) tested comprised the principal coordinate analysis (**a**) (PCA)-only and the population structure (**b**) (Q)-only. The four mixed linear models (MLM) tested comprised the (**c**) Q + D, (**d**) Q + K, (**e**) PCA + D, (**f**) PCA + K models, where D and K are the Distance and Kinship Matrices, respectively.  The black line is the expected −log_10_ *p*-value distribution, while the colored lines are the observed −log_10_ *p*-value distribution for each of the eight RSA traits.


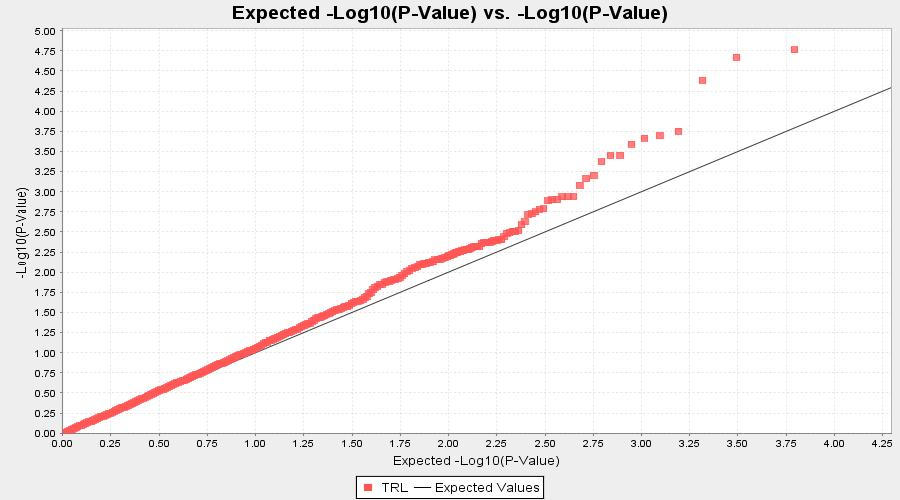

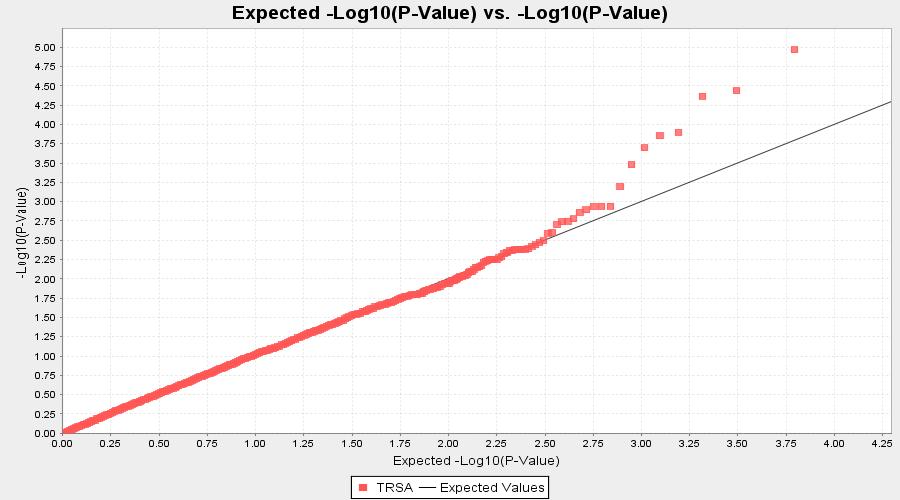


**(a)**  **(b)**

**
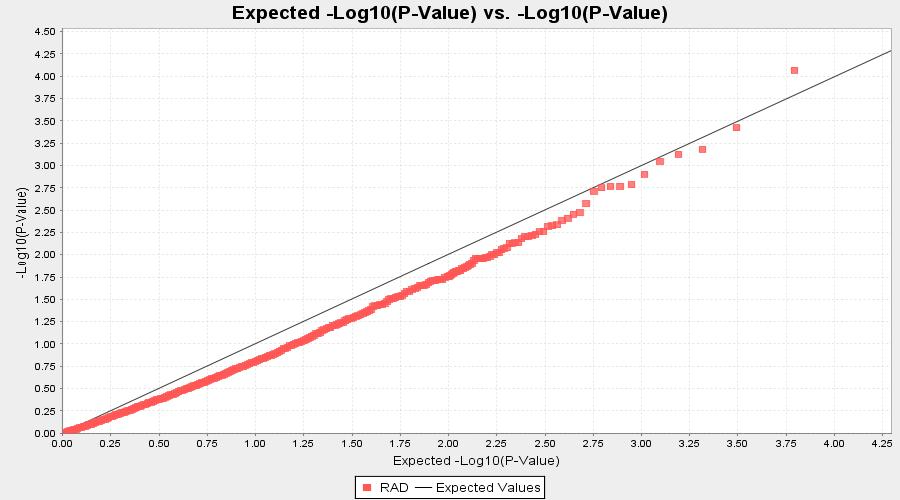

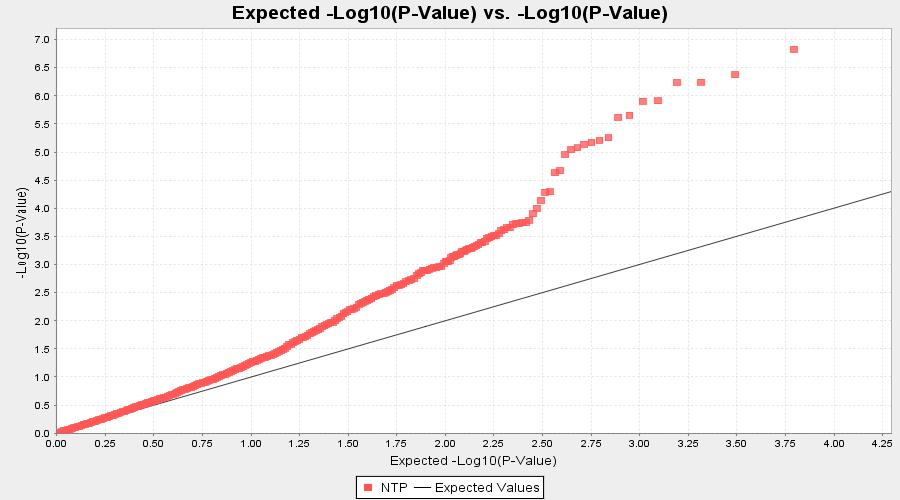
**

**(c)**  **(d)**

**Supplementary Figure 4a.** Quantile-Quantile comparison of GWAS model PCA + K for identifying loci associated with eight root architectural traits in 313 *Brassica* accessions representing five species *B. napus*, *B. oleracea*, *B. rapa*, *B. carinata*, and *B. juncea* except *B. nigra*. Traits include (**a**) total root length (TRL/cm), (**b**) total surface area of roots (TRSA/cm^2^), (**c**) root average diameter (RAD/cm), (**d**) number of tips (NTP), (**e**) total primary root length (TPRL/cm), (**f**) total lateral root length (TLRL/cm), (**g**) total tertiary root length (TTRL/cm), and (**h**) basal link length (BLL/cm). The black line is the expected −log_10_ *p*-value distribution while colored lines are the observed −log_10_ *p*-value distribution for each of the eight root architectural traits.



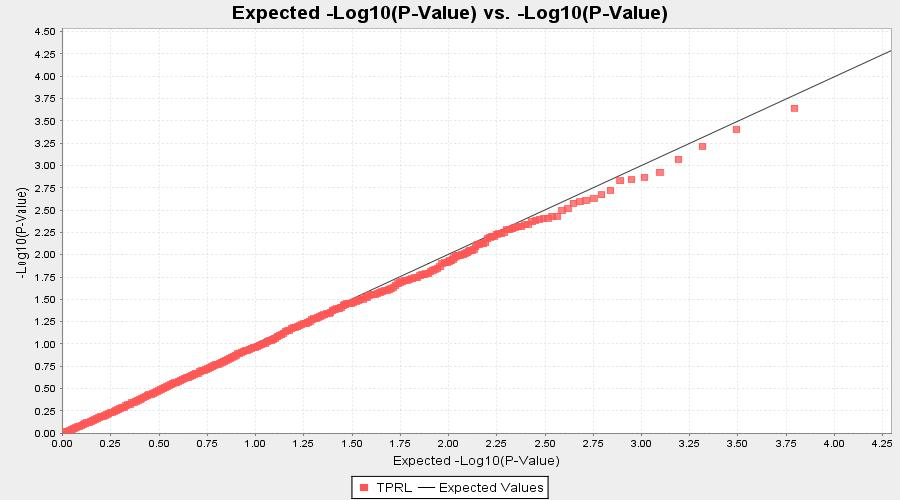

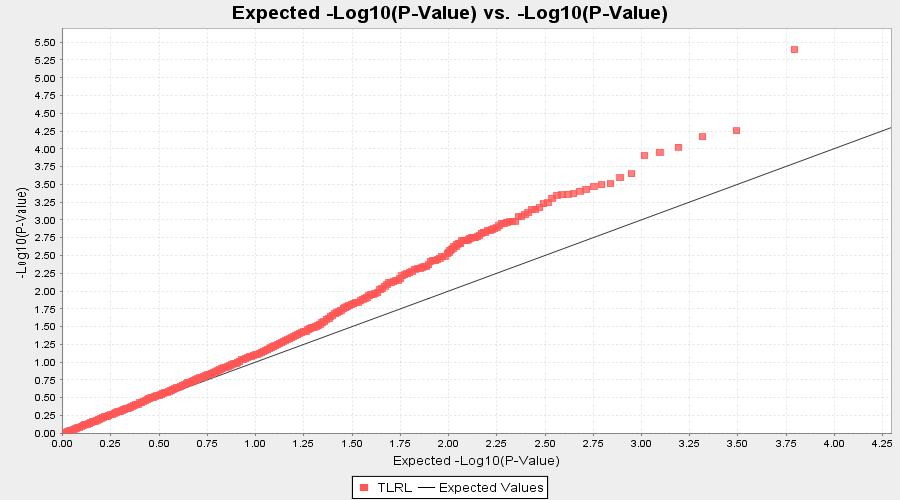


**(e)**  **(f)**

**
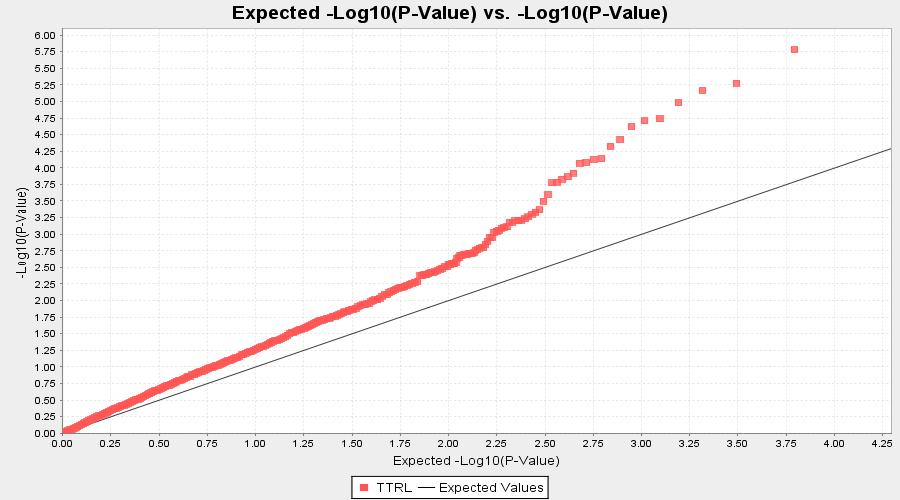

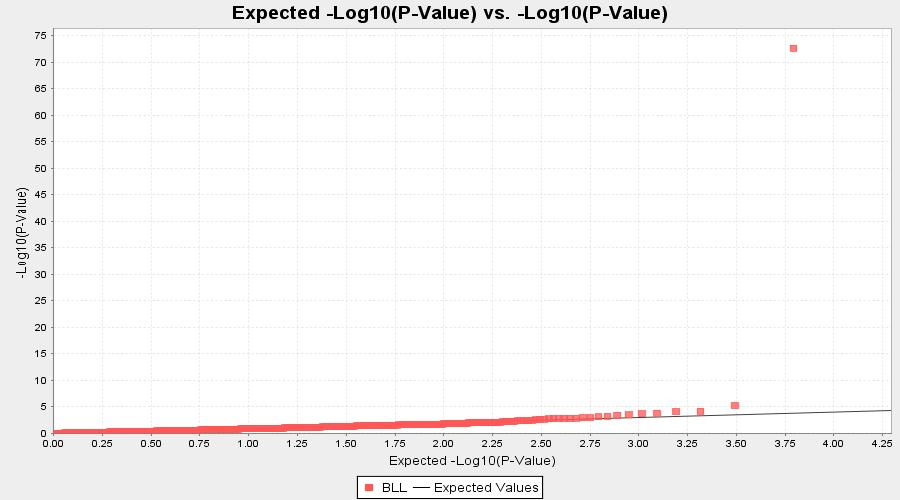
**

**(g)**  **(h)**

**Supplementary Figure 4a (continued).** Quantile-Quantile comparison of GWAS model PCA + K for identifying loci associated with eight root architectural traits in 313 *Brassica* accessions representing five species *B. napus*, *B. oleracea*, *B. rapa*, *B. carinata*, and *B. juncea* except *B. nigra*. Traits include (**a**) total root length (TRL/cm), (**b**) total surface area of roots (TRSA/cm^2^), (**c**) root average diameter (RAD/cm), (**d**) number of tips (NTP), (**e**) total primary root length (TPRL/cm), (**f**) total lateral root length (TLRL/cm), (**g**) total tertiary root length (TTRL/cm), and (**h**) basal link length (BLL/cm). The black line is the expected −log_10_ *p*-value distribution while colored lines are the observed −log_10_ *p*-value distribution for each of the eight root architectural traits.


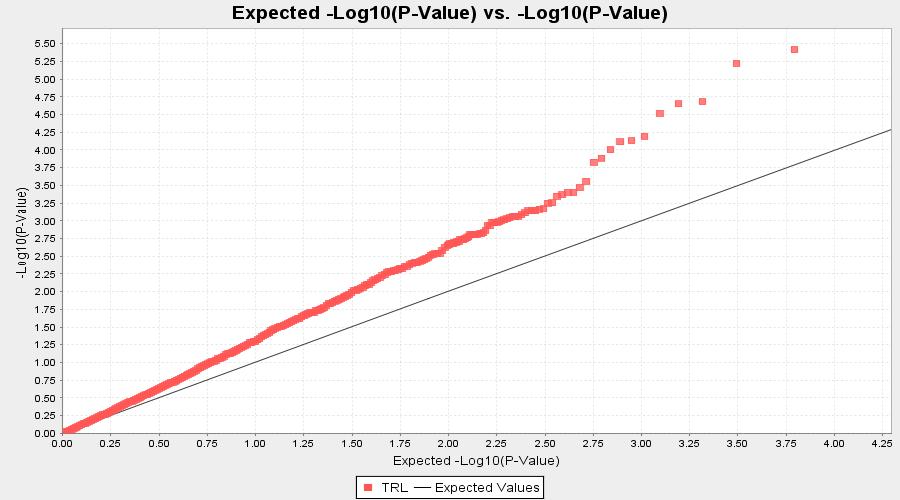

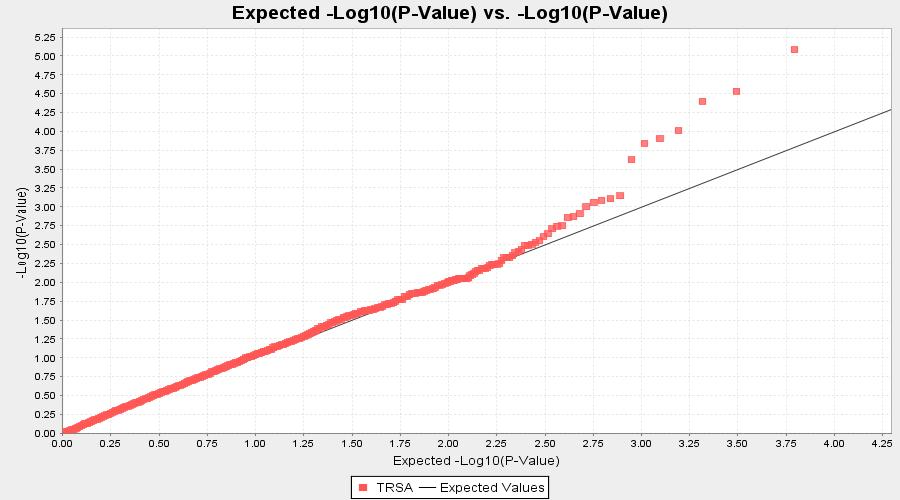


**(a)**  **(b)**

**
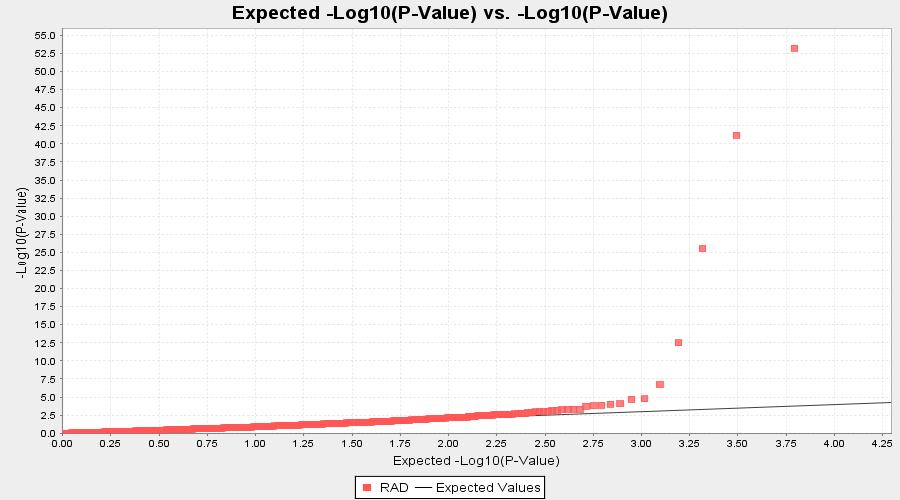

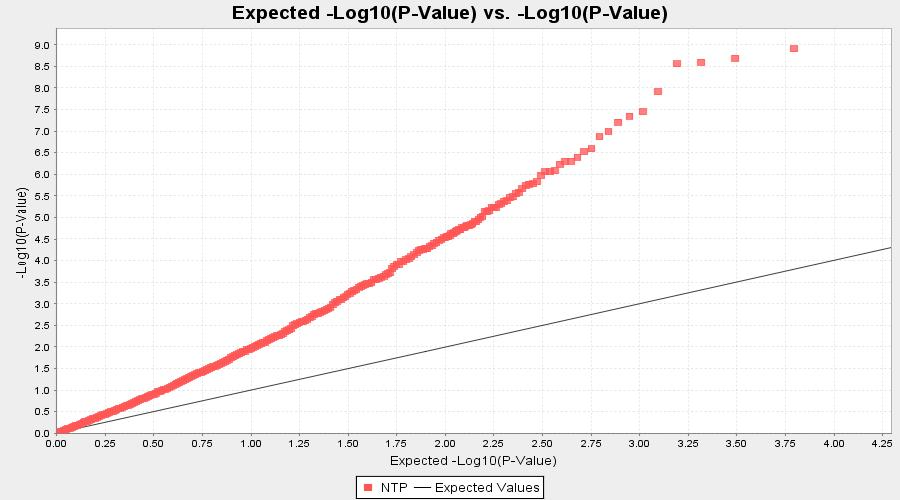
**

**(c)**  **(d)**

**Supplementary Figure 4b.** Quantile-Quantile comparison of GWAS model PCA + D for identifying loci associated with eight root architectural traits in 313 *Brassica* accessions representing five species *B. napus*, *B. oleracea*, *B. rapa*, *B. carinata*, and *B. juncea*. Traits include (**a**) total root length (TRL/cm), (**b**) total surface area of roots (TRSA/cm^2^), (**c**) root average diameter (RAD/cm), (**d**) number of tips (NTP), (**e**) total primary root length (TPRL/cm), (**f**) total lateral root length (TLRL/cm), (**g**) total tertiary root length (TTRL/cm), and (**h**) basal link length (BLL/cm). The black line is the expected −log_10_ *p*-value distribution while colored lines are the observed −log_10_ *p*-value distribution for each of the eight root architectural traits.


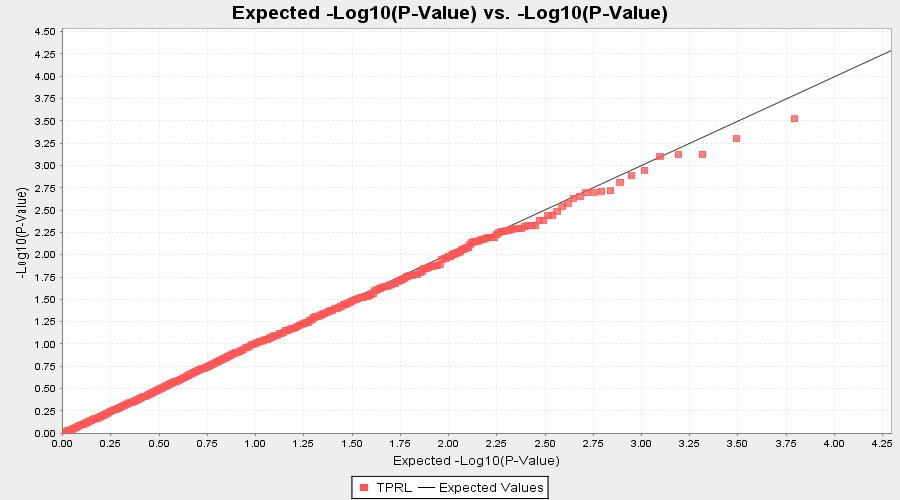

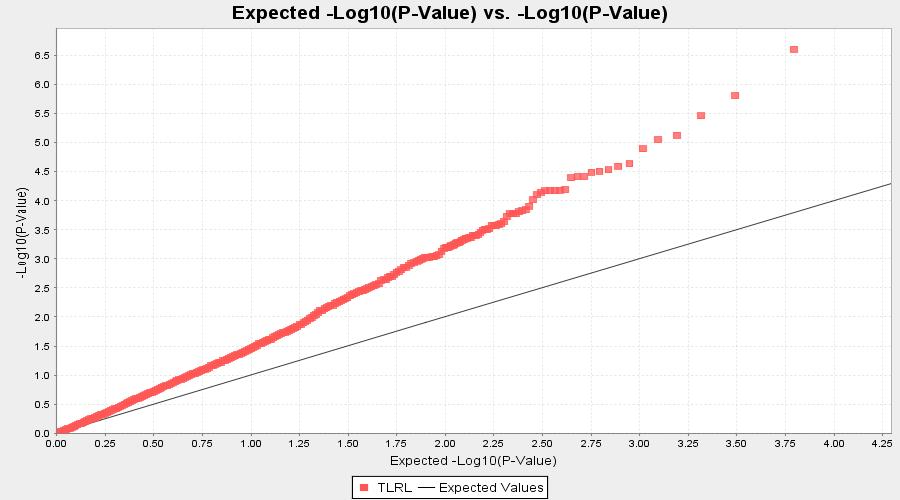


**(e)**  **(f)**

**
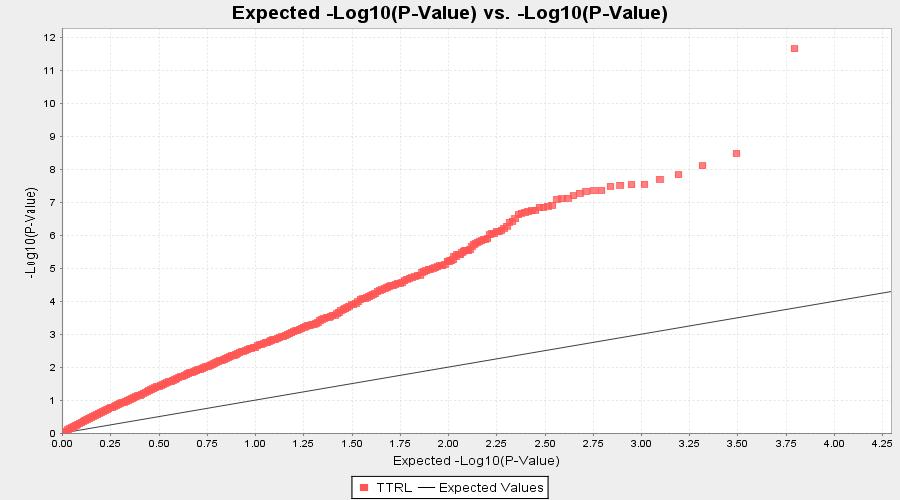

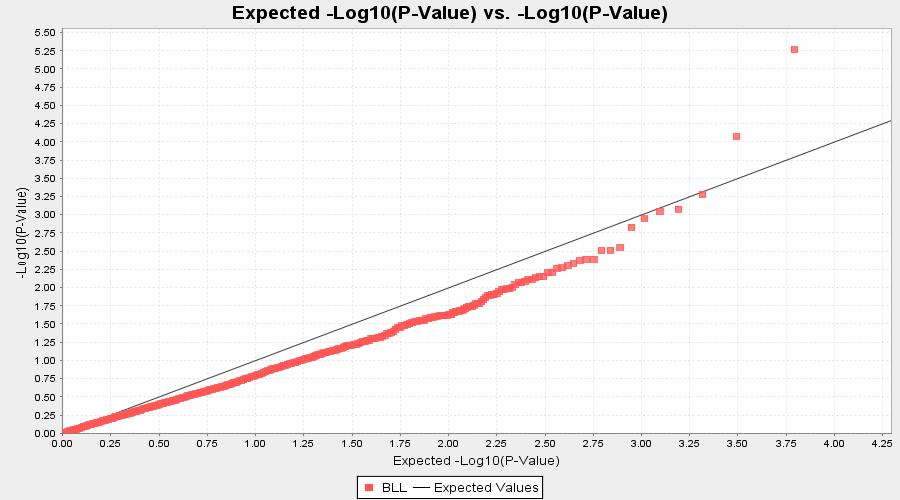
**

**(g)**  **(h)**

**Supplementary Figure 4b (continued).** Quantile-Quantile comparison of GWAS model PCA + D for identifying loci associated with eight root architectural traits in 313 *Brassica* accessions representing five species *B. napus*, *B. oleracea*, *B. rapa*, *B. carinata*, and *B. juncea*. Traits include (**a**) total root length (TRL/cm), (**b**) total surface area of roots (TRSA/cm^2^), (**c**) root average diameter (RAD/cm), (**d**) number of tips (NTP), (**e**) total primary root length (TPRL/cm), (**f**) total lateral root length (TLRL/cm), (**g**) total tertiary root length (TTRL/cm), and (**h**) basal link length (BLL/cm). The black line is the expected −log_10_ *p*-value distribution while colored lines are the observed −log_10_ *p*-value distribution for each of the eight root architectural traits.


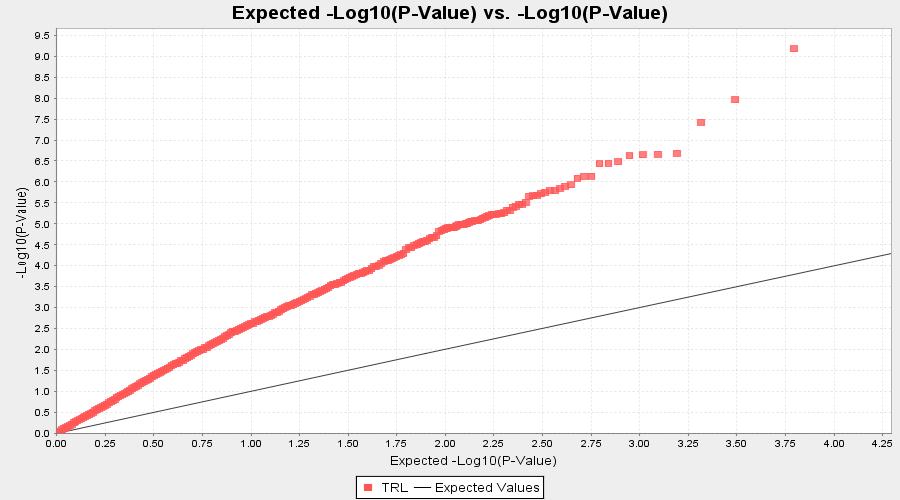

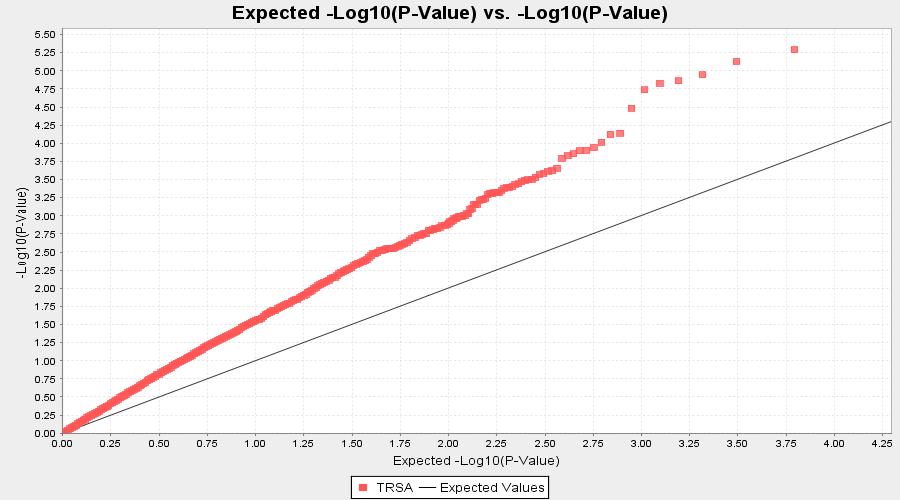


**(a)**  **(b)**

**
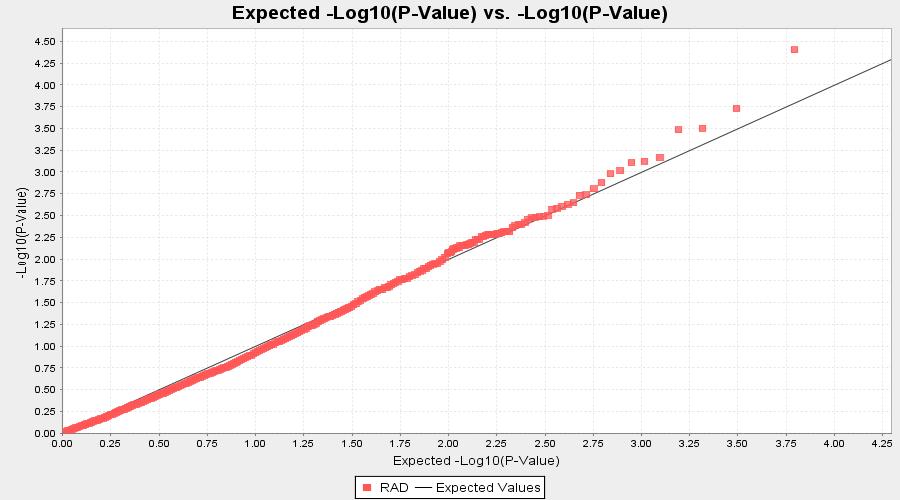

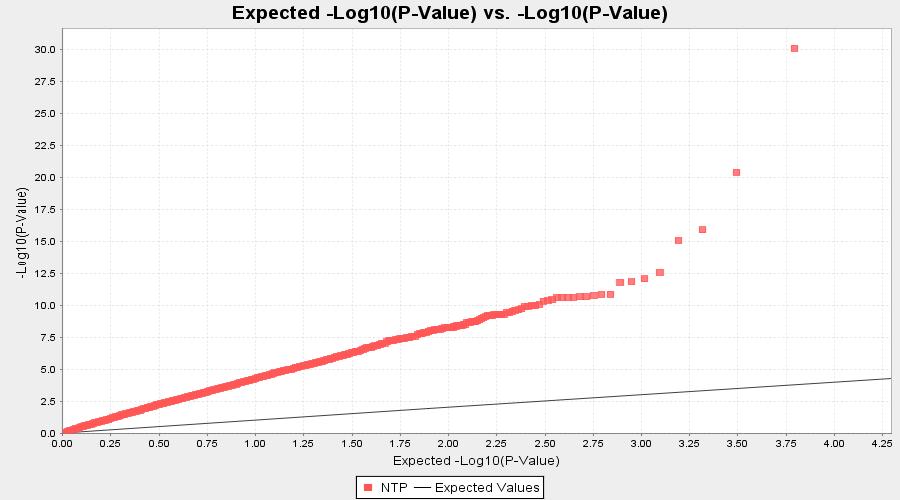
**

**(c)**  **(d)**

**Supplementary Figure 4c.** Quantile-Quantile comparison of GWAS model Q + D for identifying loci associated with eight root architectural traits in 313 *Brassica* accessions representing five species *B. napus*, *B. oleracea*, *B. rapa*, *B. carinata*, and *B. juncea*. Traits include (**a**) total root length (TRL/cm), (**b**) total surface area of roots (TRSA/cm^2^), (**c**) root average diameter (RAD/cm), (**d**) number of tips (NTP), (**e**) total primary root length (TPRL/cm), (**f**) total lateral root length (TLRL/cm), (**g**) total tertiary root length (TTRL/cm), and (**h**) basal link length (BLL/cm). The black line is the expected −log_10_ *p*-value distribution while colored lines are the observed −log_10_ *p*-value distribution for each of the eight root architectural traits.


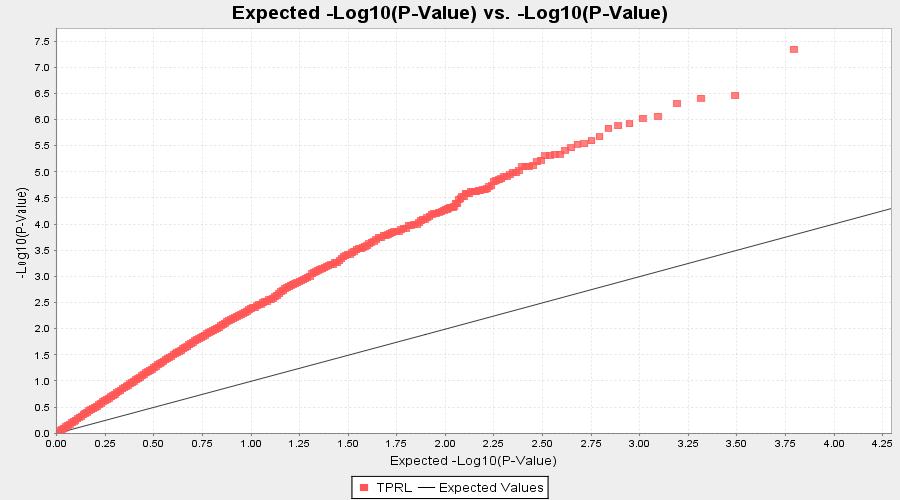

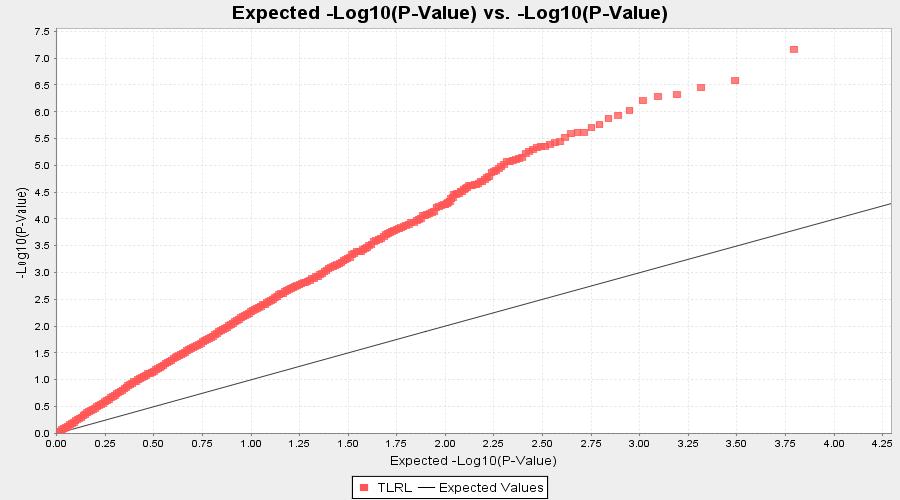


**(e)**  **(f)**

**
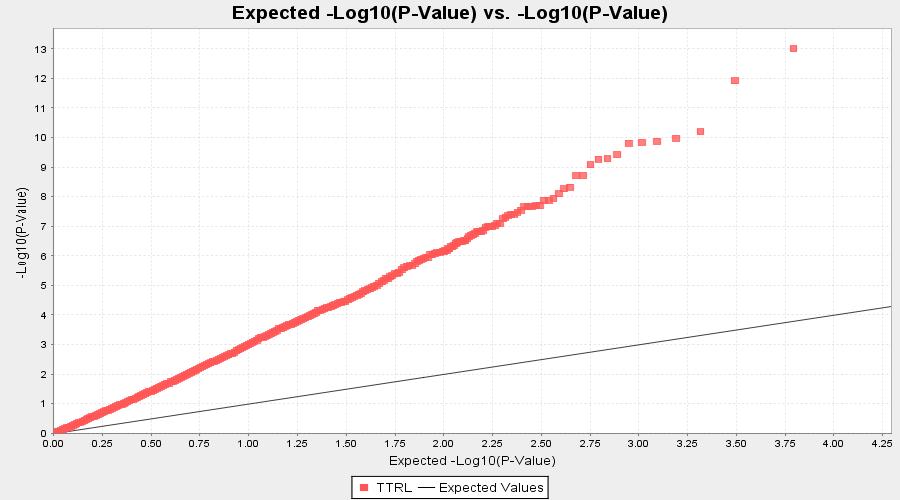

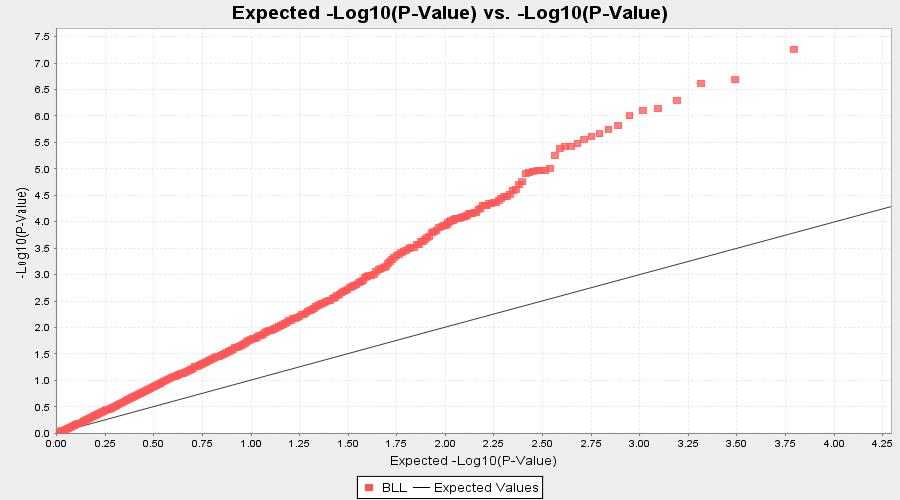
**

**(g)**  **(h)**

**Supplementary Figure 4c (continued).** Quantile-Quantile comparison of GWAS model Q + D for identifying loci associated with eight root architectural traits in 313 *Brassica* accessions representing five species *B. napus*, *B. oleracea*, *B. rapa*, *B. carinata*, and *B. juncea*. Traits include (**a**) total root length (TRL/cm), (**b**) total surface area of roots (TRSA/cm^2^), (**c**) root average diameter (RAD/cm), (**d**) number of tips (NTP), (**e**) total primary root length (TPRL/cm), (**f**) total lateral root length (TLRL/cm), (**g**) total tertiary root length (TTRL/cm), and (**h**) basal link length (BLL/cm). The black line is the expected −log_10_ *p*-value distribution while colored lines are the observed −log_10_ *p*-value distribution for each of the eight root architectural traits.


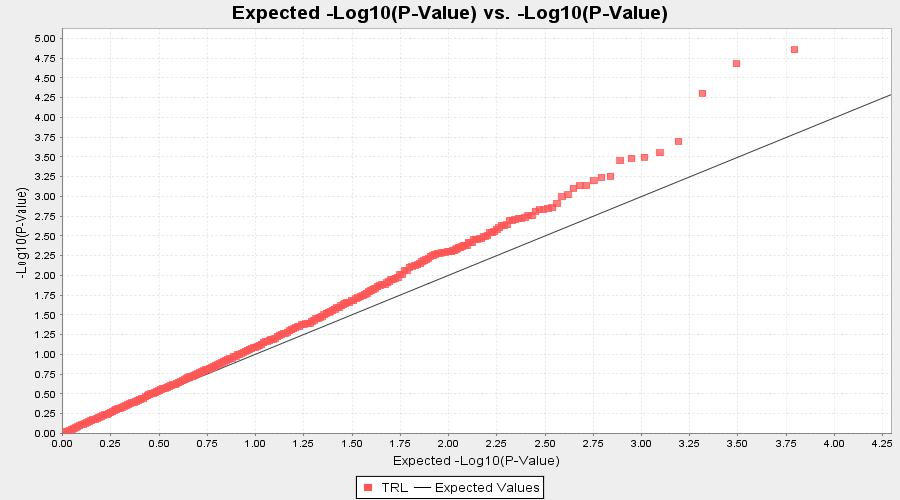

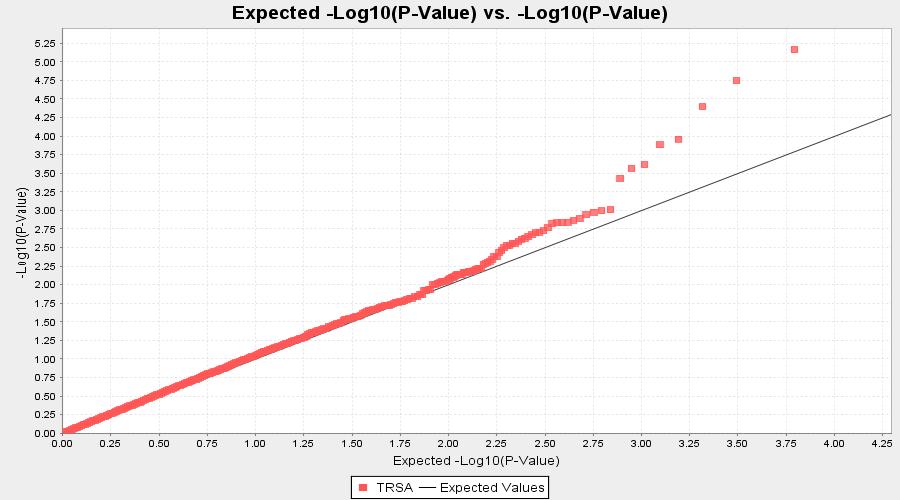


**(a)**  **(b)**

**
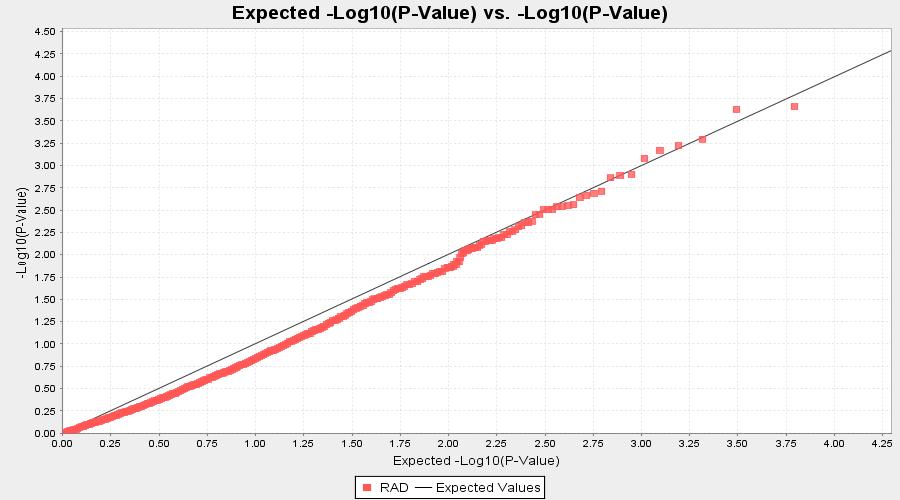

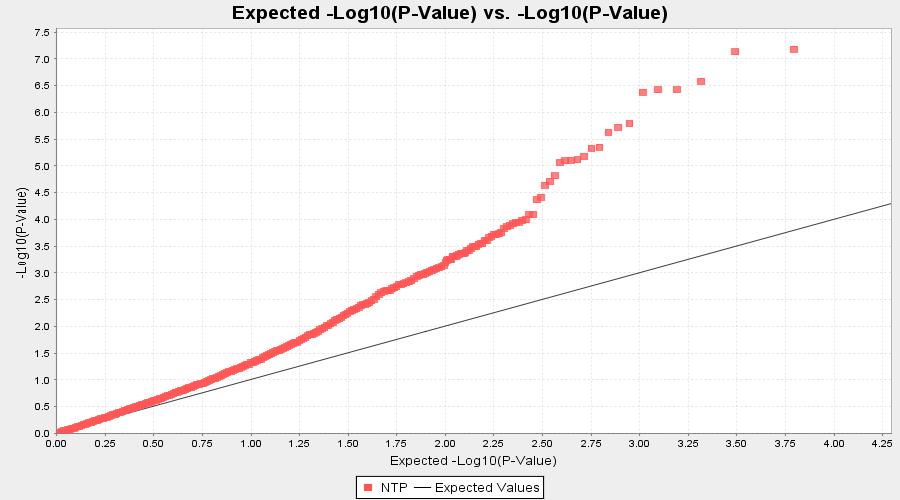
**

**(c)**  **(d)**

**Supplementary Figure 4d.** Quantile-Quantile comparison of GWAS model Q + K for identifying loci associated with eight root architectural traits in 313 *Brassica* accessions representing five species *B. napus*, *B. oleracea*, *B. rapa*, *B. carinata*, and *B. juncea*. Traits include (**a**) total root length (TRL/cm), (**b**) total surface area of roots (TRSA/cm^2^), (**c**) root average diameter (RAD/cm), (**d**) number of tips (NTP), (**e**) total primary root length (TPRL/cm), (**f**) total lateral root length (TLRL/cm), (**g**) total tertiary root length (TTRL/cm), and (**h**) basal link length (BLL/cm). The black line is the expected −log_10_ *p*-value distribution while colored lines are the observed −log_10_ *p*-value distribution for each of the eight root architectural traits.


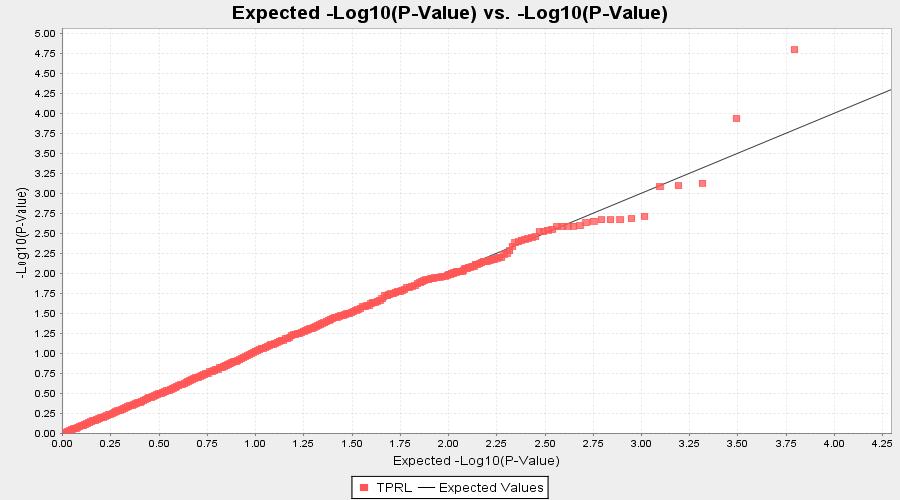

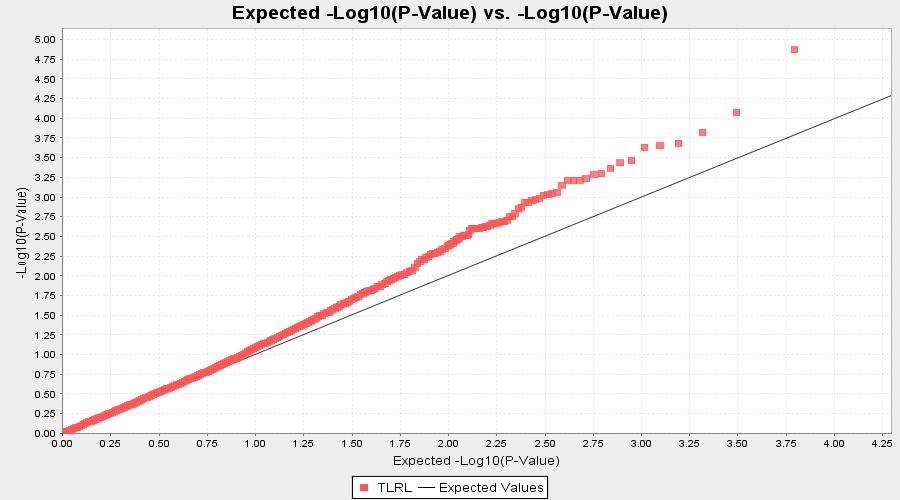


**(e)**  **(f)**

**
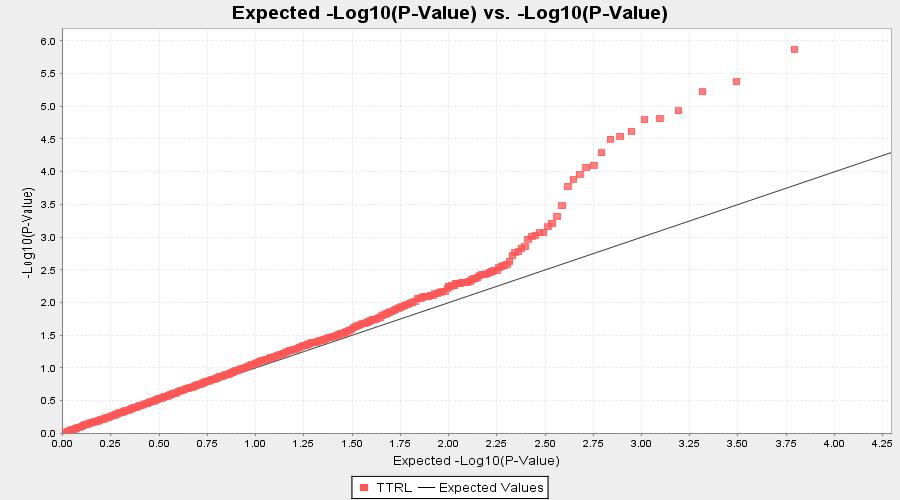

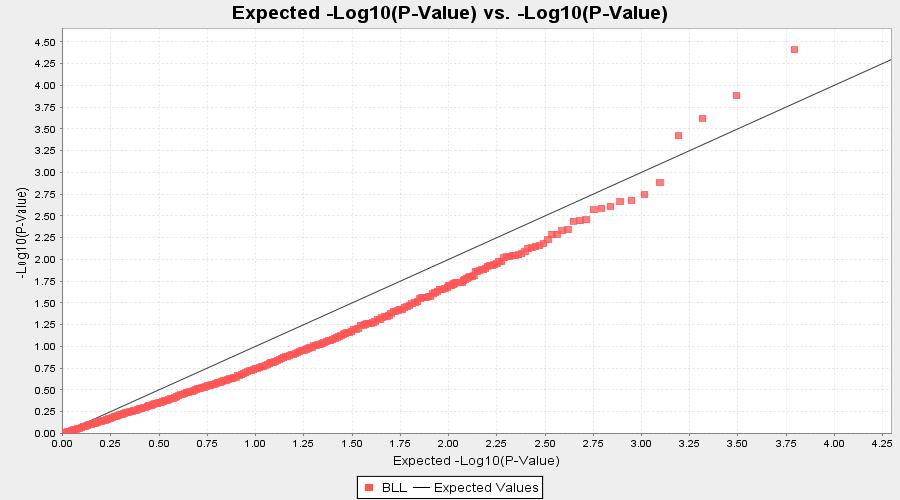
**

**(g)**  **(h)**

**Supplementary Figure 4d (continued).** Quantile-Quantile comparison of GWAS model Q + K for identifying loci associated with eight root architectural traits in 313 *Brassica* accessions representing five species *B. napus*, *B. oleracea*, *B. rapa*, *B. carinata*, and *B. juncea*. Traits include (**a**) total root length (TRL/cm), (**b**) total surface area of roots (TRSA/cm^2^), (**c**) root average diameter (RAD/cm), (**d**) number of tips (NTP), (**e**) total primary root length (TPRL/cm), (**f**) total lateral root length (TLRL/cm), (**g**) total tertiary root length (TTRL/cm), and (**h**) basal link length (BLL/cm). The black line is the expected −log_10_ *p*-value distribution while colored lines are the observed −log_10_ *p*-value distribution for each of the eight root architectural traits.


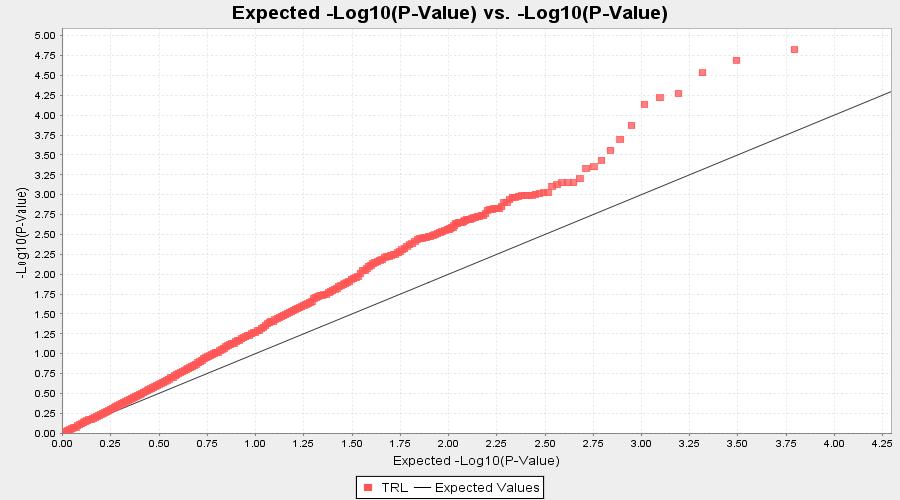

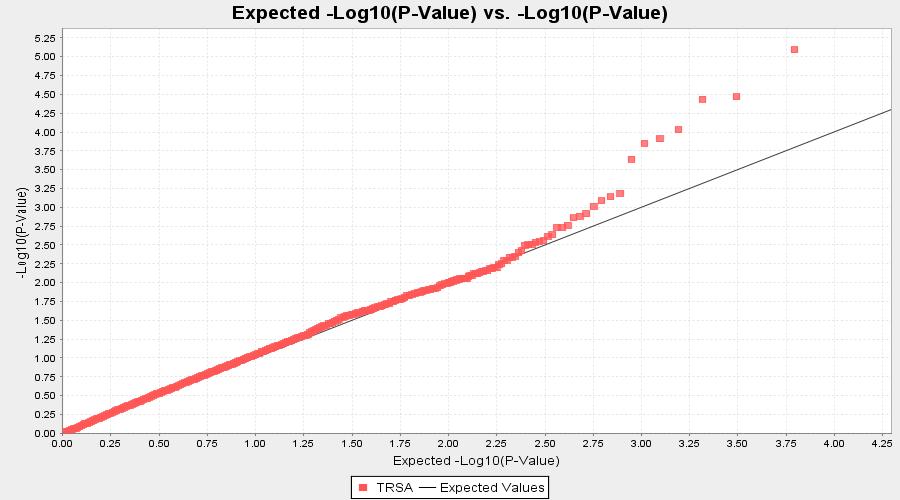


**(a)**  **(b)**

**
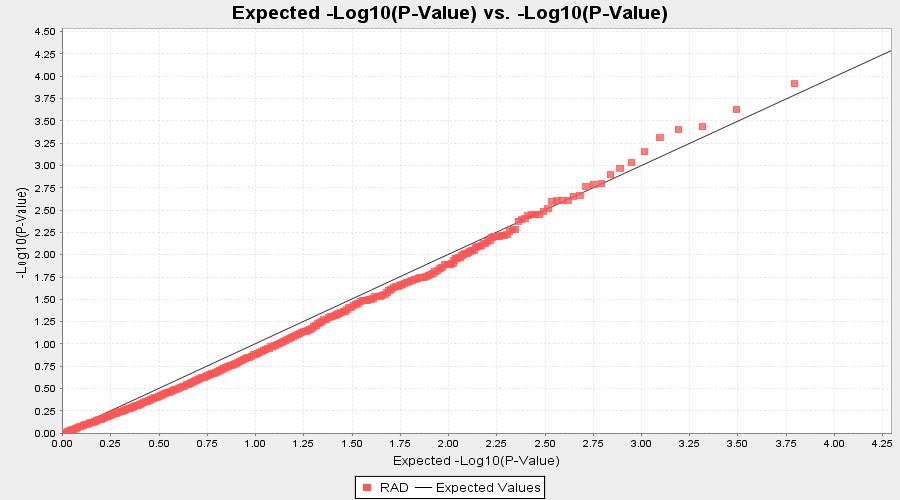

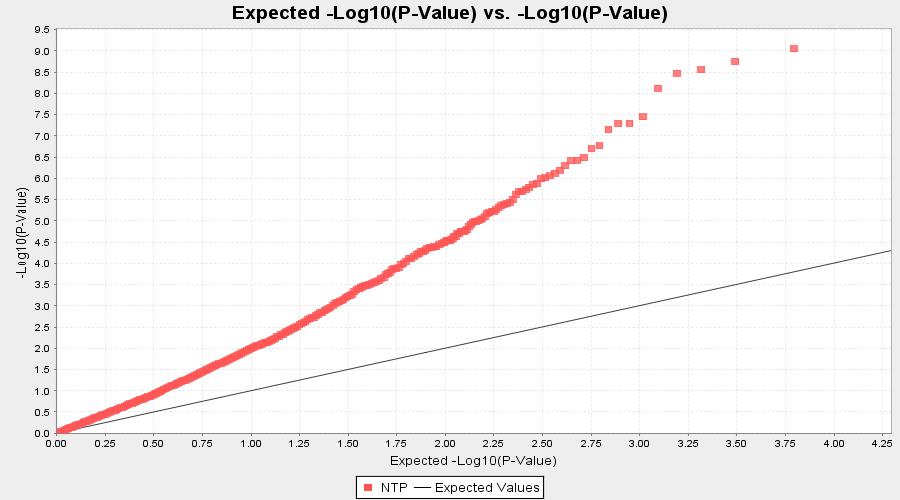
**

**(c)**  **(d)**

**Supplementary Figure 4e.** Quantile-Quantile comparison of GWAS model PCA-only for identifying loci associated with eight root architectural traits in 313 *Brassica* accessions representing five species *B. napus*, *B. oleracea*, *B. rapa*, *B. carinata*, and *B. juncea*. Traits include (**a**) total root length (TRL/cm), (**b**) total surface area of roots (TRSA/cm^2^), (**c**) root average diameter (RAD/cm), (**d**) number of tips (NTP), (**e**) total primary root length (TPRL/cm), (**f**) total lateral root length (TLRL/cm), (**g**) total tertiary root length (TTRL/cm), and (**h**) basal link length (BLL/cm). The black line is the expected −log_10_ *p*-value distribution while colored lines are the observed −log_10_ *p*-value distribution for each of the eight root architectural traits.


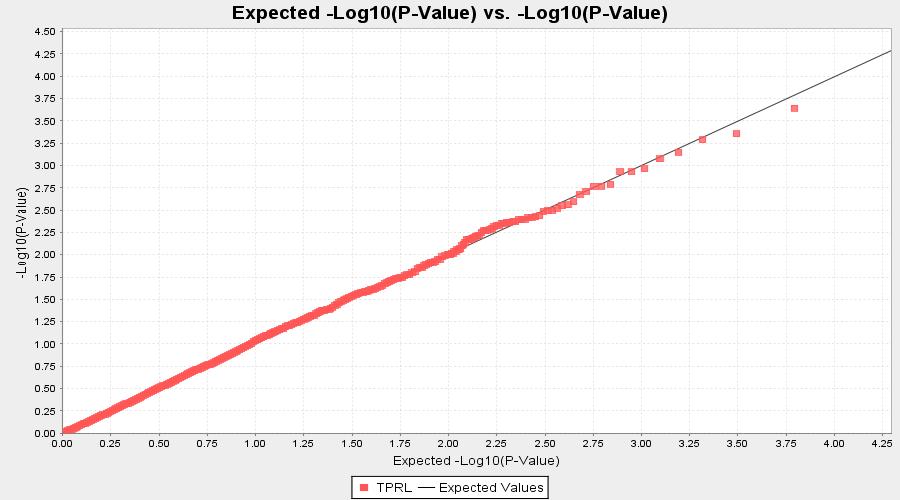

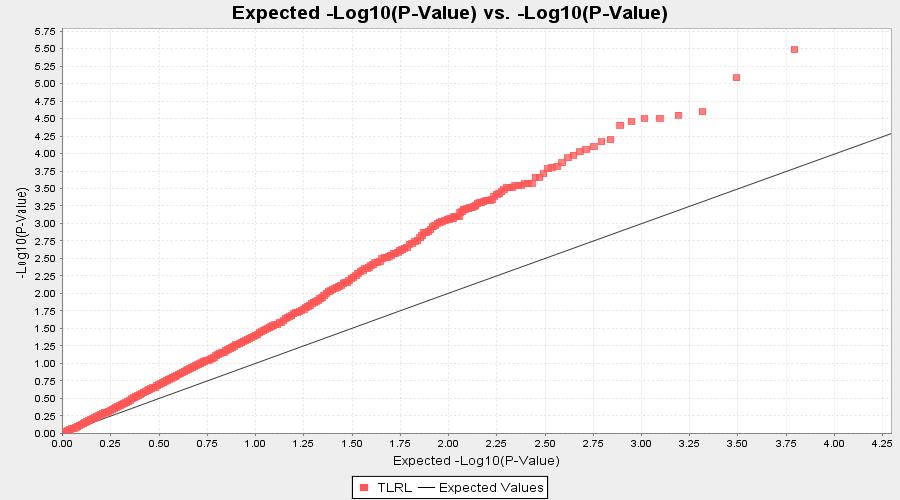


**(e)**  **(f)**

**
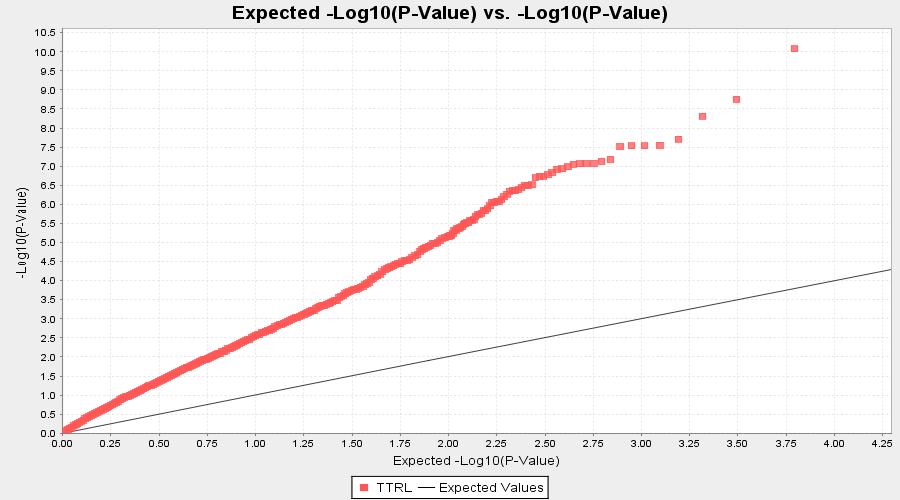

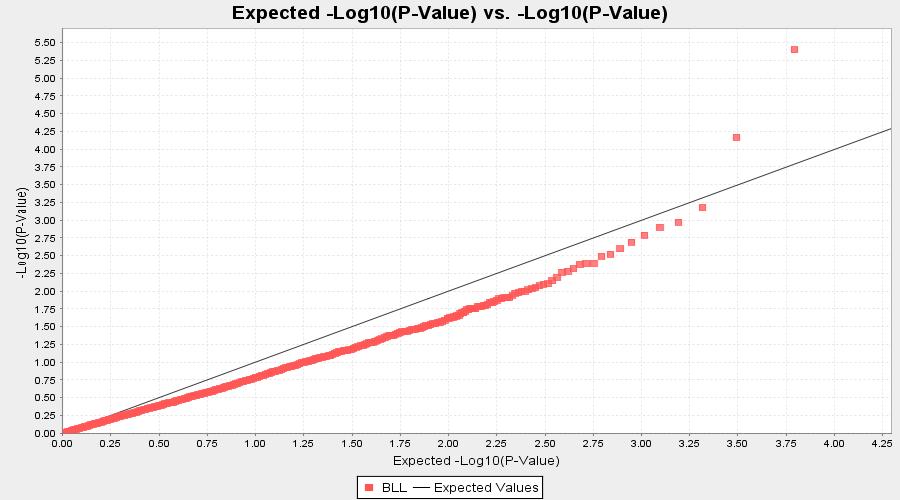
**

**(g)**  **(h)**

**Supplementary Figure 4e (continued).** Quantile-Quantile comparison of GWAS model PCA-only for identifying loci associated with eight root architectural traits in 313 *Brassica* accessions representing five species *B. napus*, *B. oleracea*, *B. rapa*, *B. carinata*, and *B. juncea*. Traits include (**a**) total root length (TRL/cm), (**b**) total surface area of roots (TRSA/cm^2^), (**c**) root average diameter (RAD/cm), (**d**) number of tips (NTP), (**e**) total primary root length (TPRL/cm), (**f**) total lateral root length (TLRL/cm), (**g**) total tertiary root length (TTRL/cm), and (**h**) basal link length (BLL/cm). The black line is the expected −log_10_ *p*-value distribution while colored lines are the observed −log_10_ *p*-value distribution for each of the eight root architectural traits.


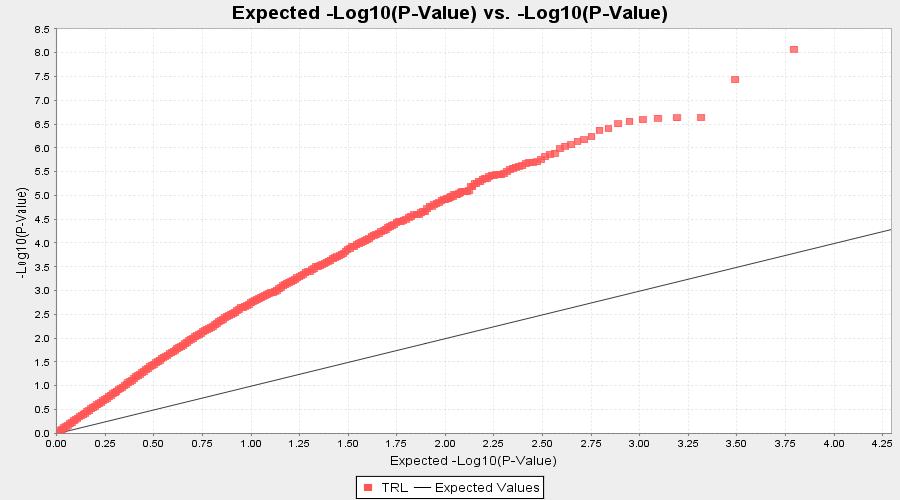

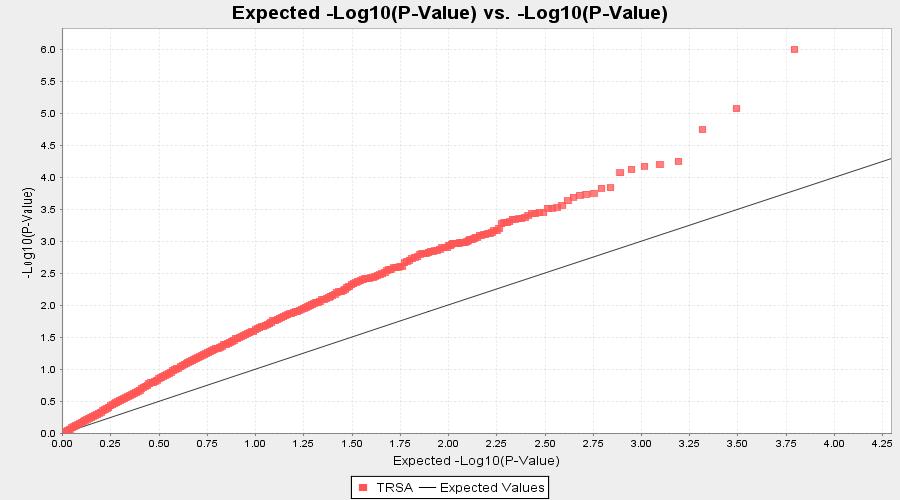


**(a)**  **(b)**

**
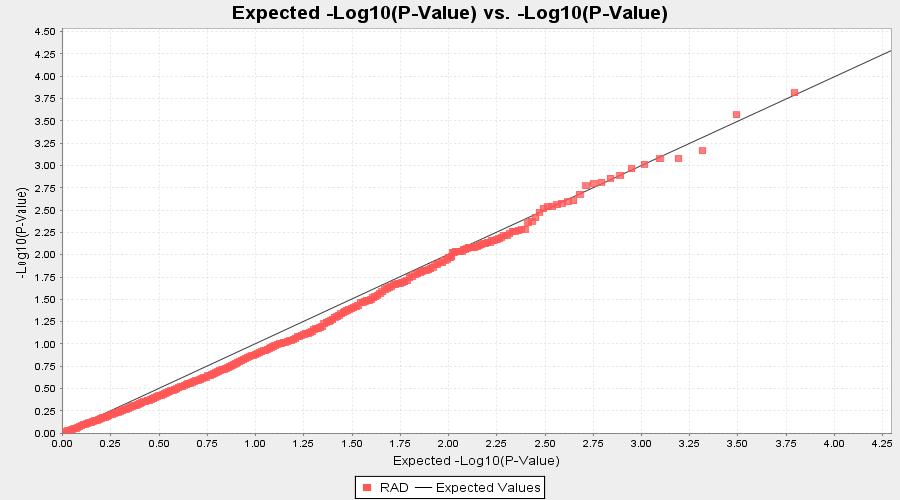
** **
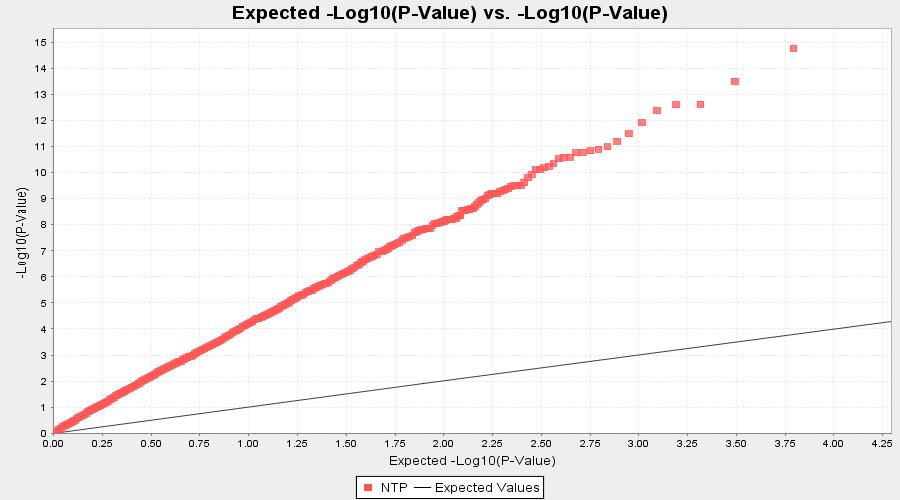
**

**(c)**  **(d)**

**Supplementary Figure 4f.** Quantile-Quantile comparison of GWAS model Q-only for identifying loci associated with eight root architectural traits in 313 *Brassica* accessions representing five species *B. napus*, *B. oleracea*, *B. rapa*, *B. carinata*, and *B. juncea*. Traits include (**a**) total root length (TRL/cm), (**b**) total surface area of roots (TRSA/cm^2^), (**c**) root average diameter (RAD/cm), (**d**) number of tips (NTP), (**e**) total primary root length (TPRL/cm), (**f**) total lateral root length (TLRL/cm), (**g**) total tertiary root length (TTRL/cm), and (**h**) basal link length (BLL/cm). The black line is the expected −log_10_ *p*-value distribution while colored lines are the observed −log_10_ *p*-value distribution for each of the eight root architectural traits.


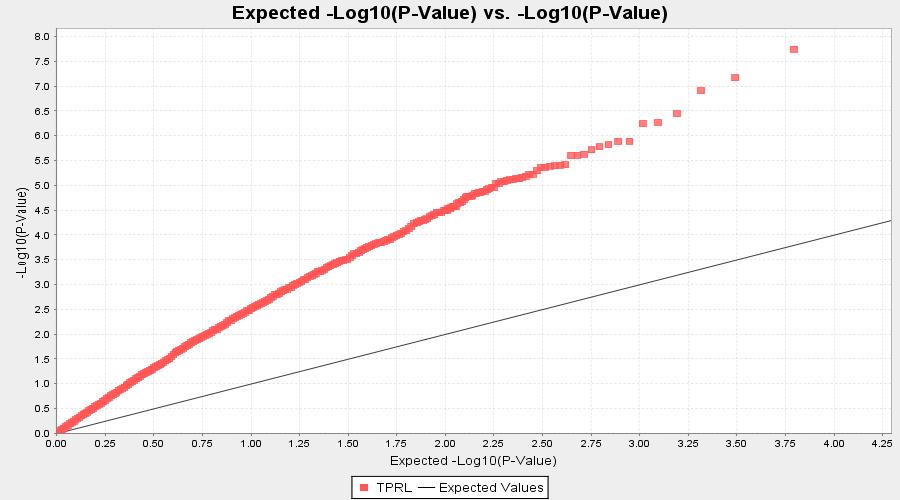

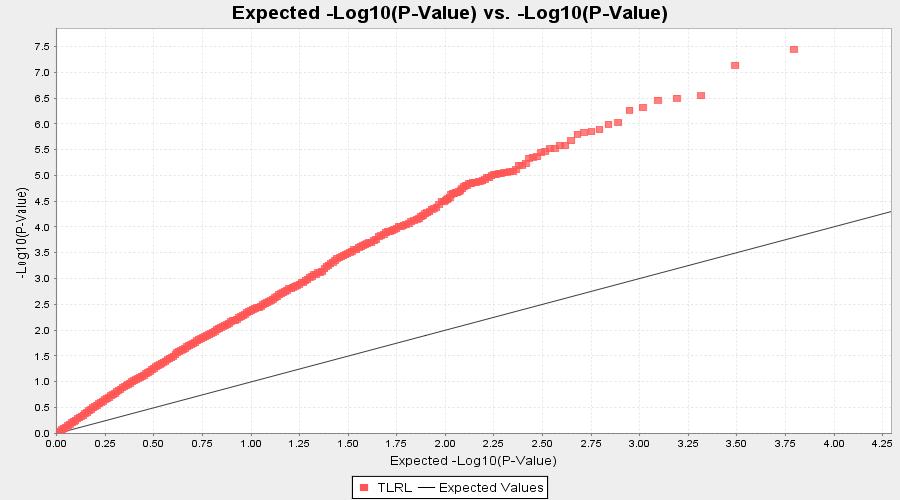


**(e)**  **(f)**

**
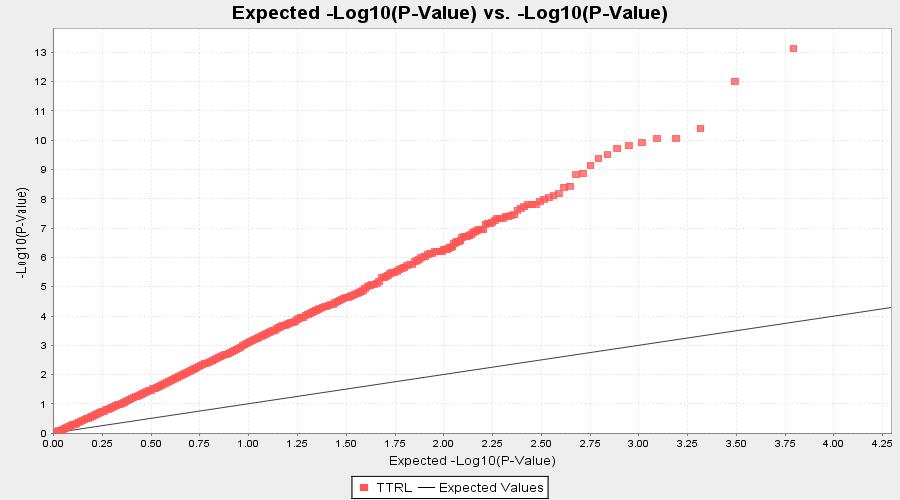
** **
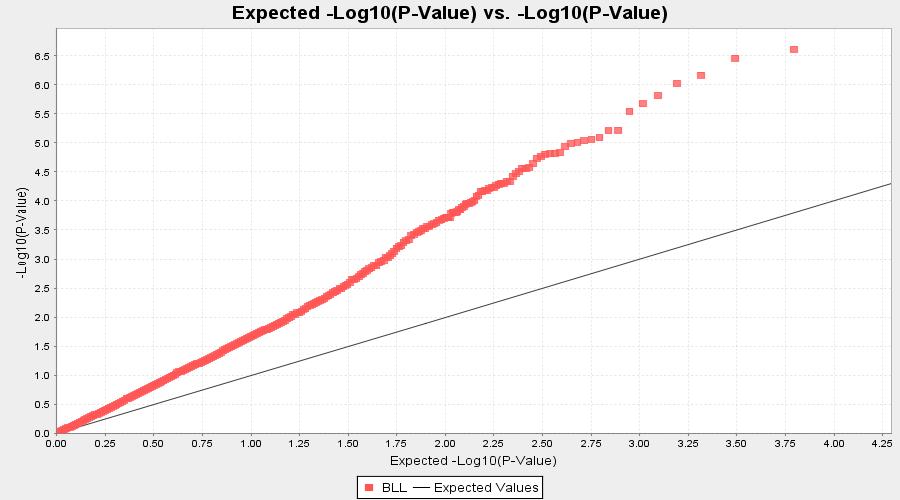
**

**(g)**  **(h)**

**Supplementary Figure 4f (continued).** Quantile-Quantile comparison of GWAS model Q-only for identifying loci associated with eight root architectural traits in 313 *Brassica* accessions representing five species *B. napus*, *B. oleracea*, *B. rapa*, *B. carinata*, and *B. juncea*. Traits include (**a**) total root length (TRL/cm), (**b**) total surface area of roots (TRSA/cm^2^), (**c**) root average diameter (RAD/cm), (**d**) number of tips (NTP), (**e**) total primary root length (TPRL/cm), (**f**) total lateral root length (TLRL/cm), (**g**) total tertiary root length (TTRL/cm), and (**h**) basal link length (BLL/cm). The black line is the expected −log_10_ *p*-value distribution while colored lines are the observed −log_10_ *p*-value distribution for each of the eight root architectural traits.


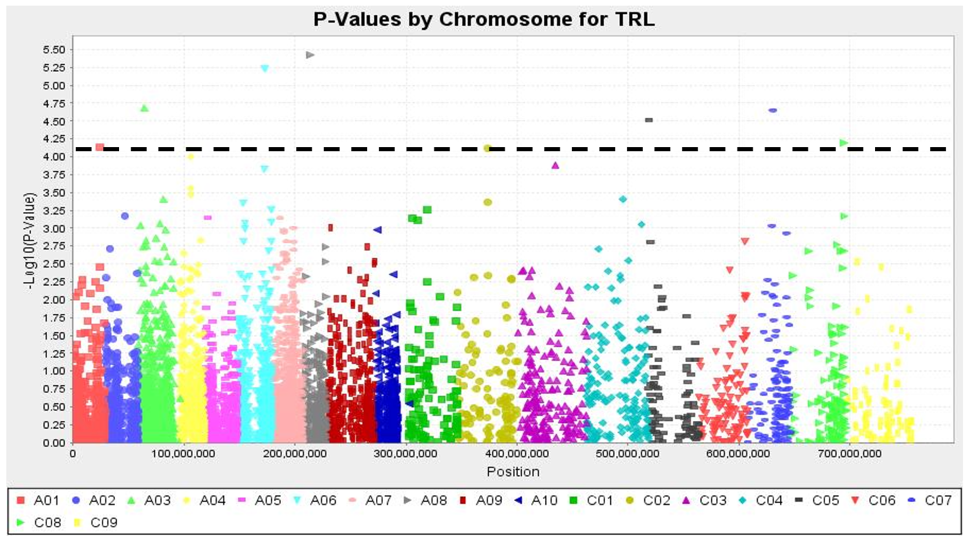

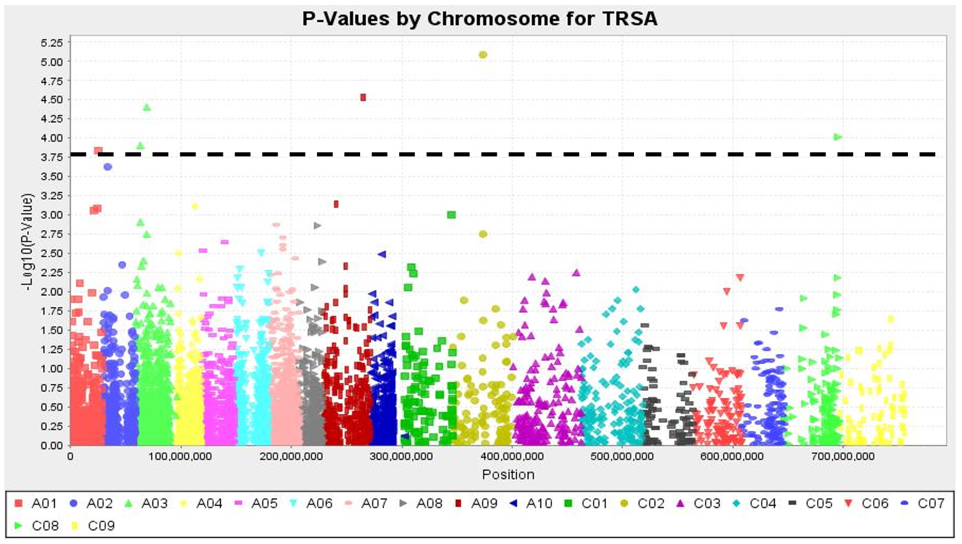


**(a)**  **(b)**

**
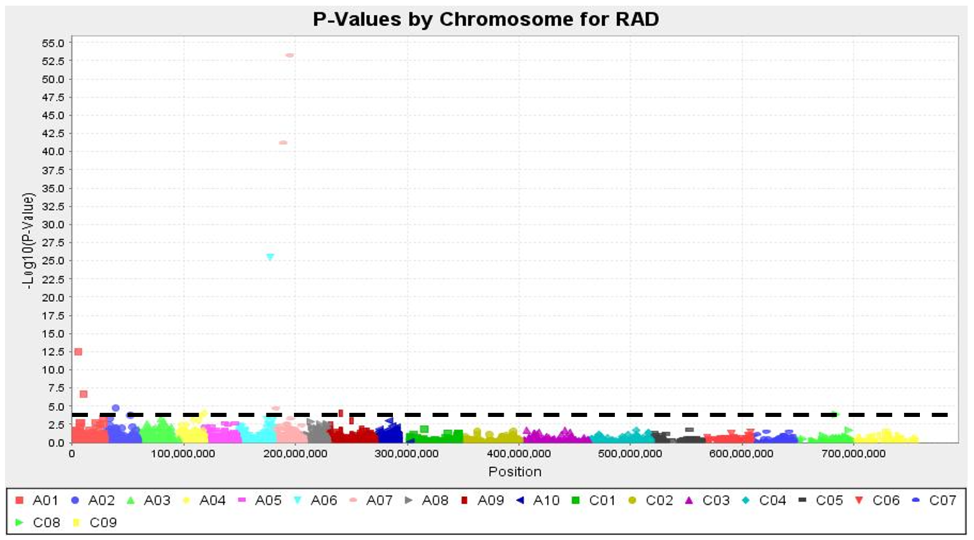

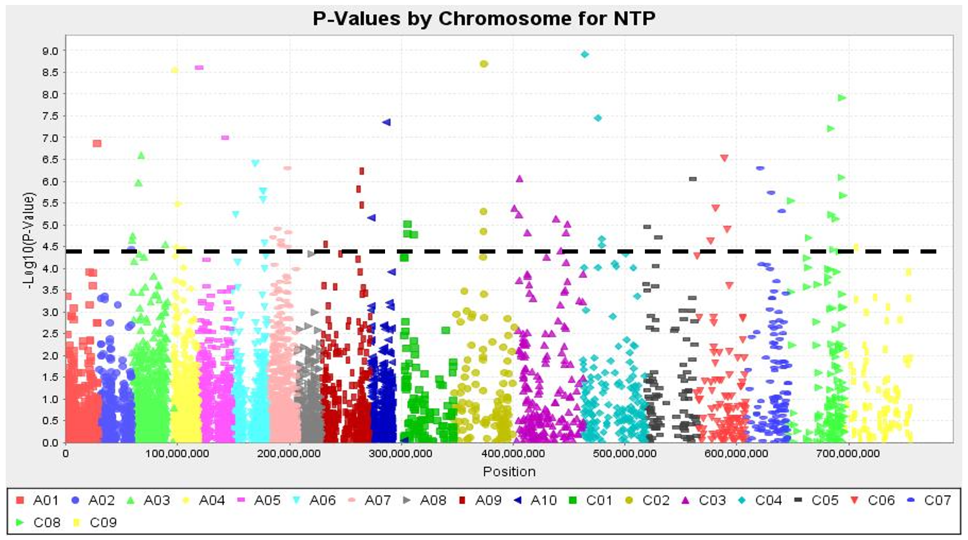
**

**(c)**  **(d)**

**Supplementary Figure 5a.** Manhattan plots of the PCA + D MLM models for identifying root architecture traits loci in 313 *Brassica* accessions representing five species *B. napus*, *B. oleracea*, *B. rapa*, *B. carinata*, and *B. juncea*. The dashed horizontal lines indicate the Bonferroni-adjusted significance threshold known as “logarithm-of-odds” (LOD score). The dots above the significance threshold indicate SNPs associated with resistance to each trait.


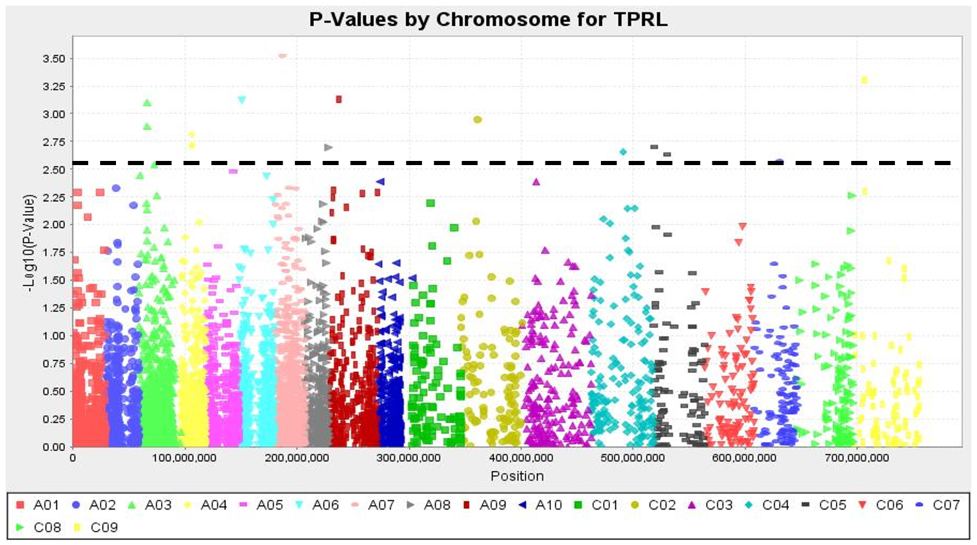

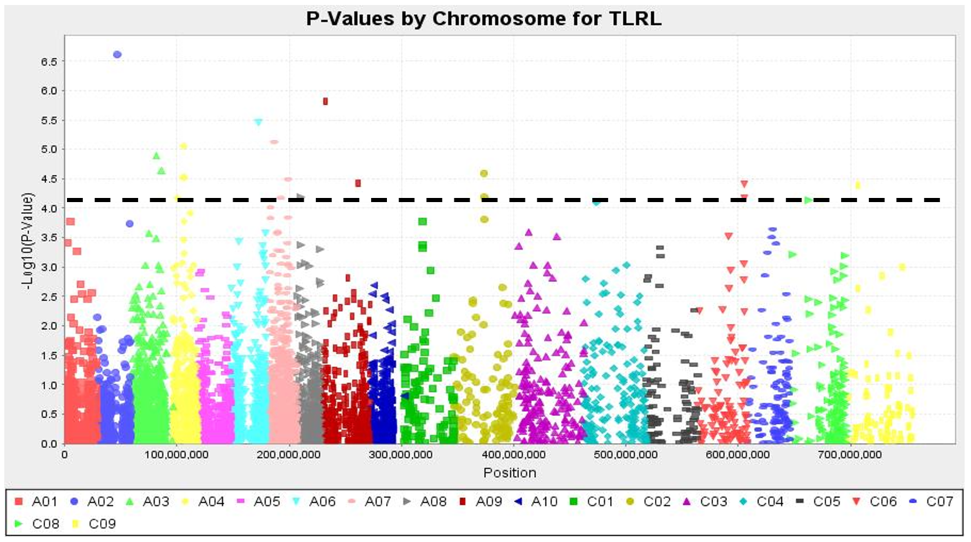


**(e)**  **(f)**

**
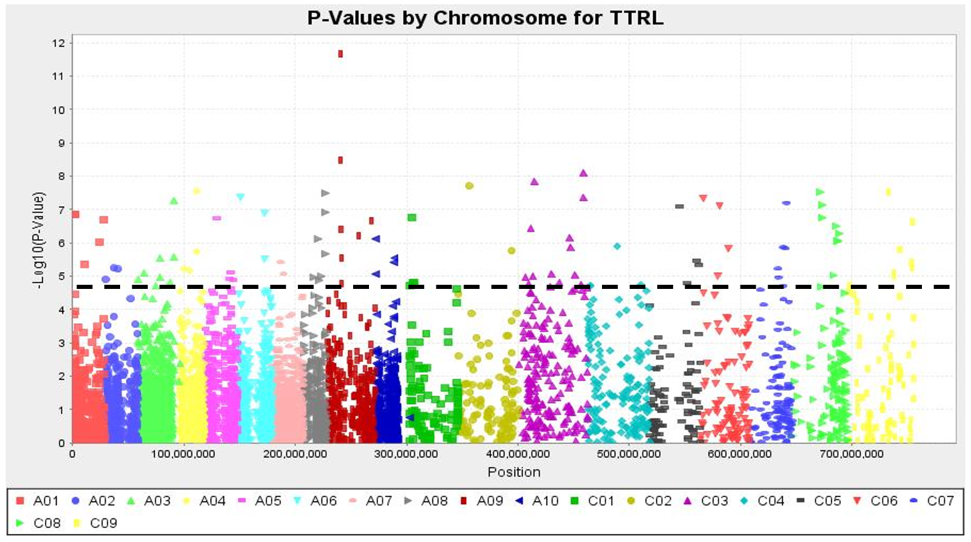

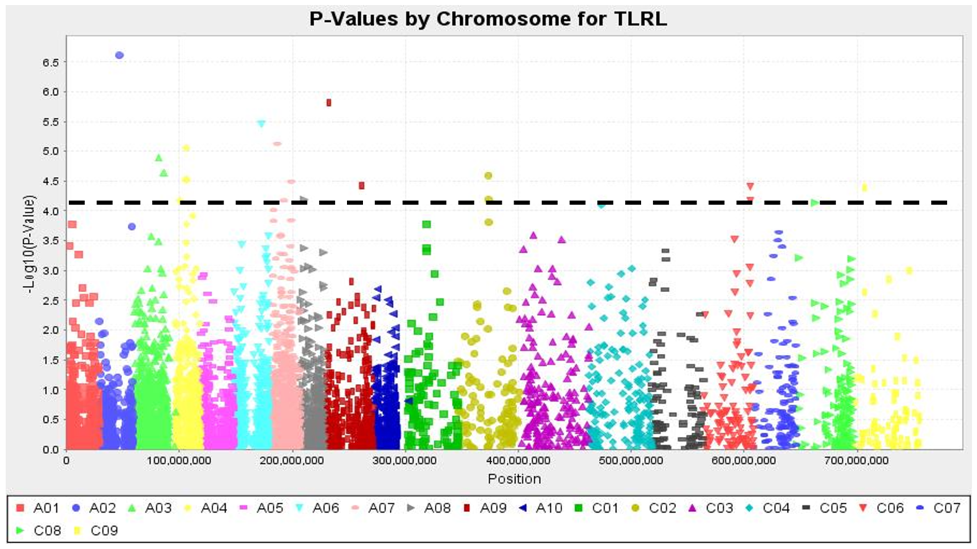
**

**(g)**  **(h)**

**Supplementary Figure 5a (continued).** Manhattan plots of the PCA + D MLM models for identifying root architecture traits loci in 313 *Brassica* accessions representing five species *B. napus*, *B. oleracea*, *B. rapa*, *B. carinata*, and *B. juncea*. The dashed horizontal lines indicate the Bonferroni-adjusted significance threshold known as “logarithm-of-odds” (LOD score). The dots above the significance threshold indicate SNPs associated with resistance to each trait.


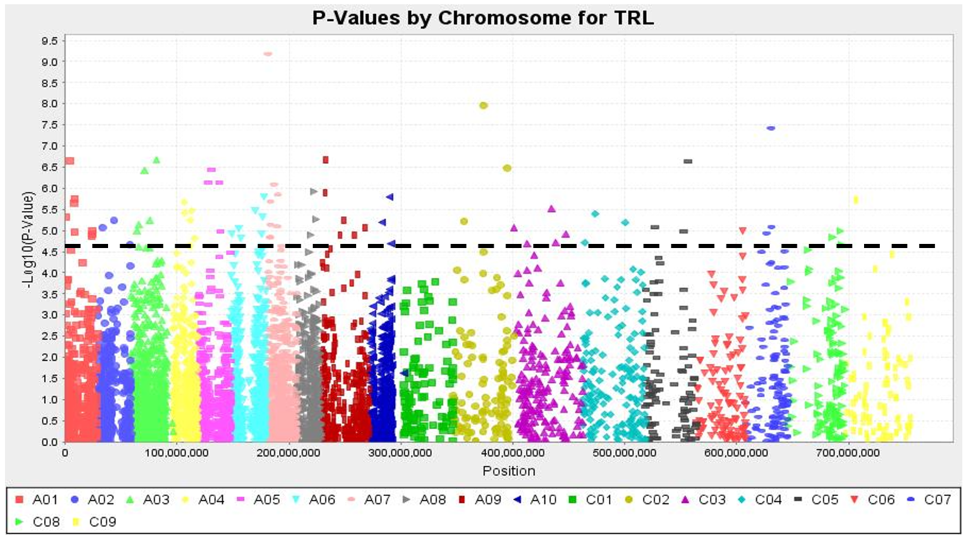

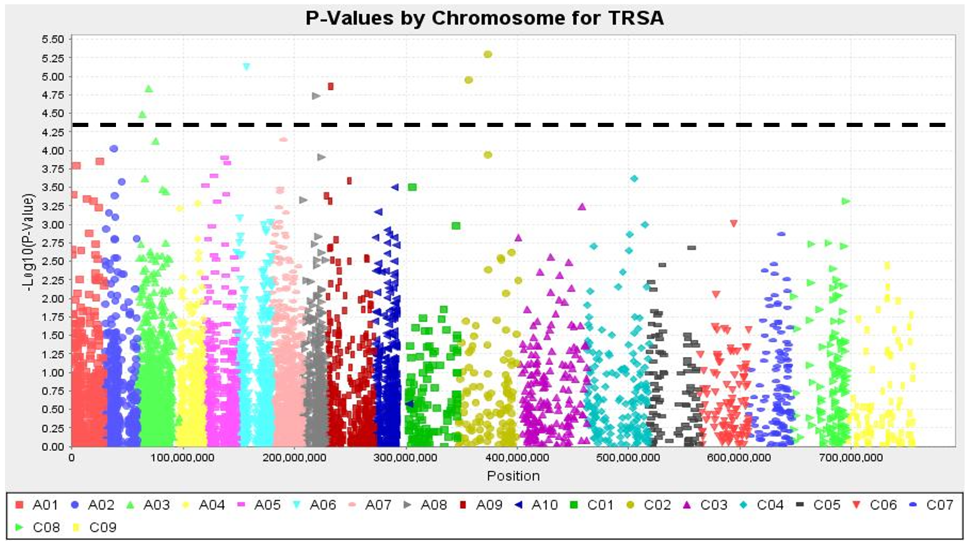


**(a)**  **(b)**

**
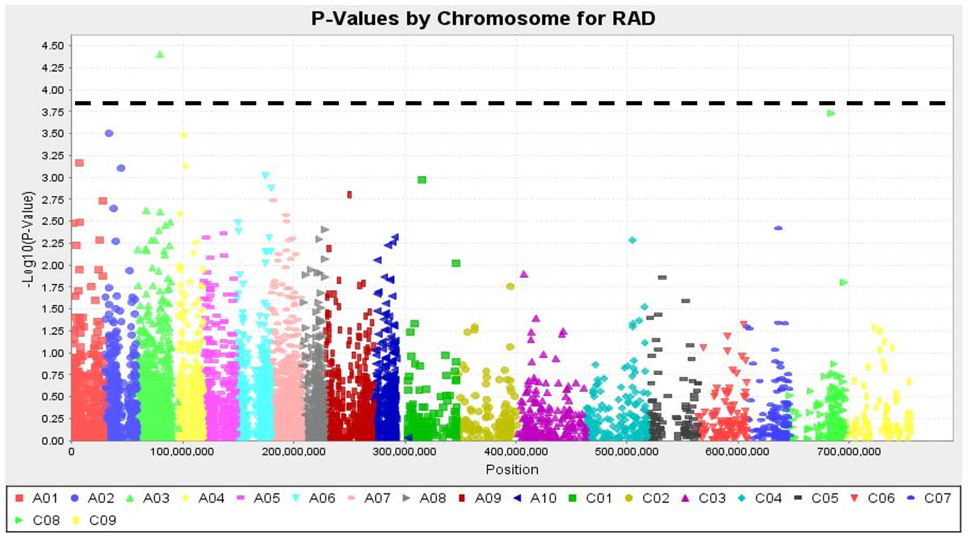

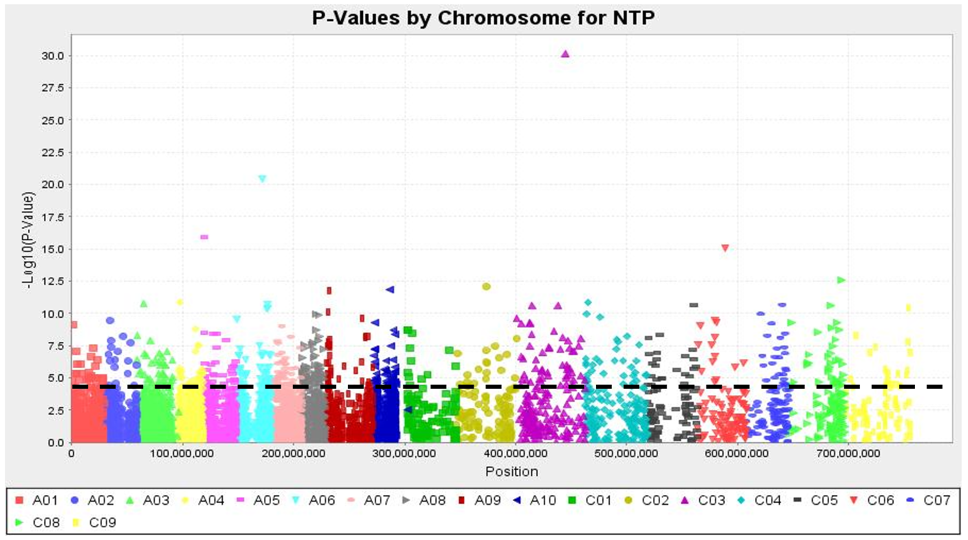
**

**(c)**  **(d)**

**Supplementary Figure 5b.** Manhattan plots of the Q + D MLM models for identifying root architecture traits loci in 313 *Brassica* accessions representing five species *B. napus*, *B. oleracea*, *B. rapa*, *B. carinata*, and *B. juncea*. The dashed horizontal lines indicate the Bonferroni-adjusted significance threshold known as “logarithm-of-odds” (LOD score). The dots above the significance threshold indicate SNPs associated with resistance to each trait.


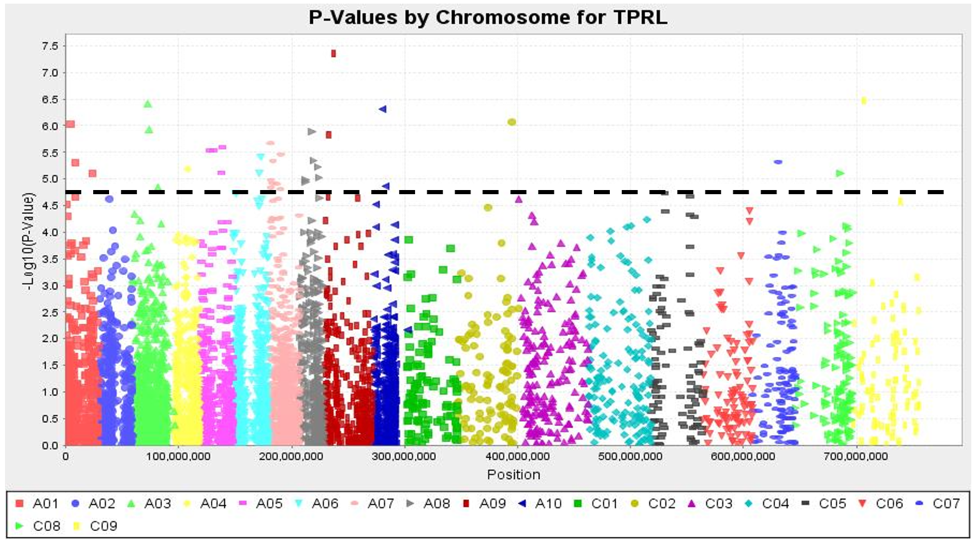

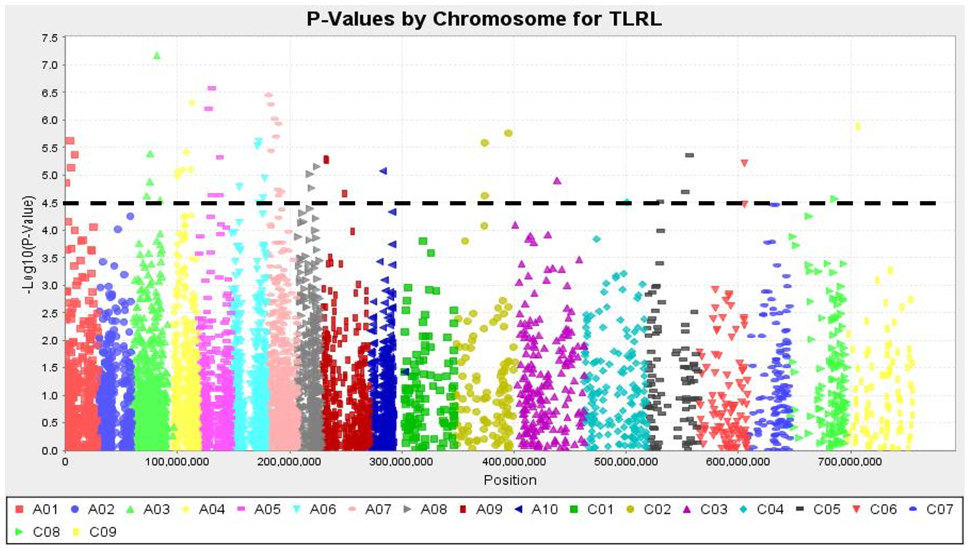


**(e)**  **(f)**

**
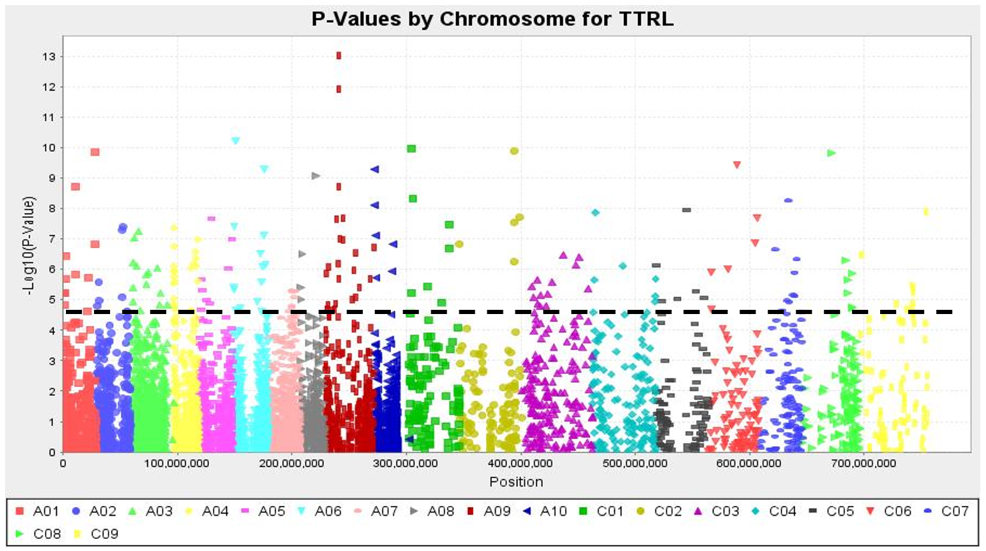

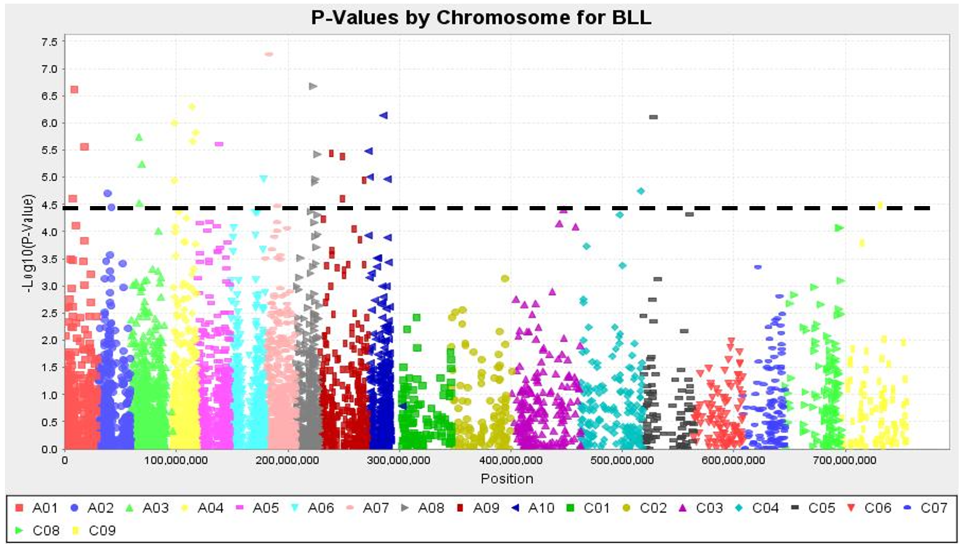
**

**(g)**  **(h)**

**Supplementary Figure 5b (continued).** Manhattan plots of the Q + D MLM models for identifying root architecture traits loci in 313 *Brassica* accessions representing five species *B. napus*, *B. oleracea*, *B. rapa*, *B. carinata*, and *B. juncea*. The dashed horizontal lines indicate the Bonferroni-adjusted significance threshold known as “logarithm-of-odds” (LOD score). The dots above the significance threshold indicate SNPs associated with resistance to each trait.


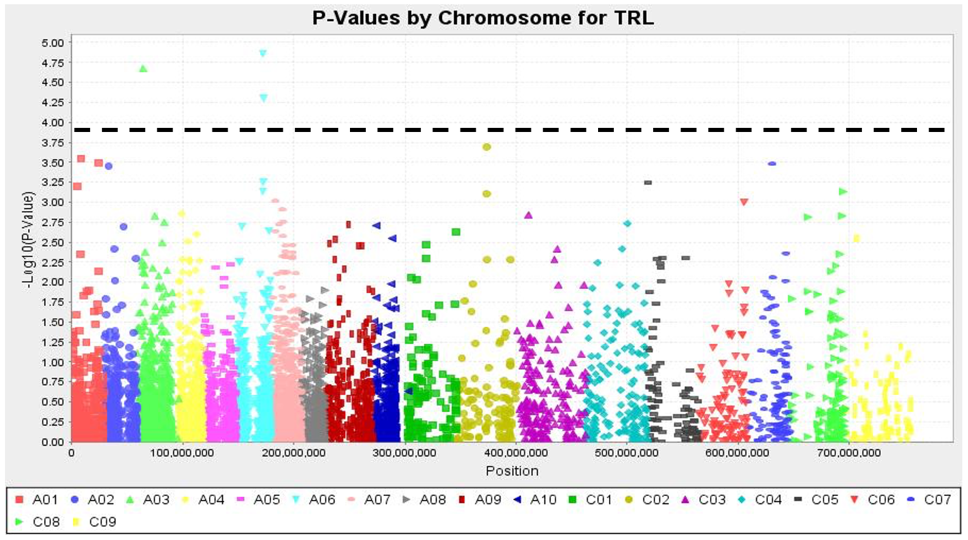

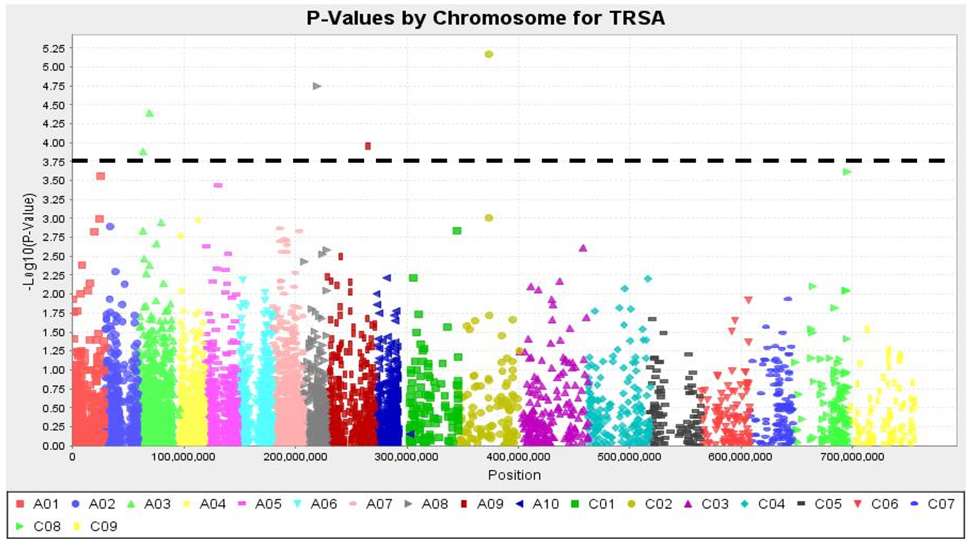


**(a)**  **(b)**

**
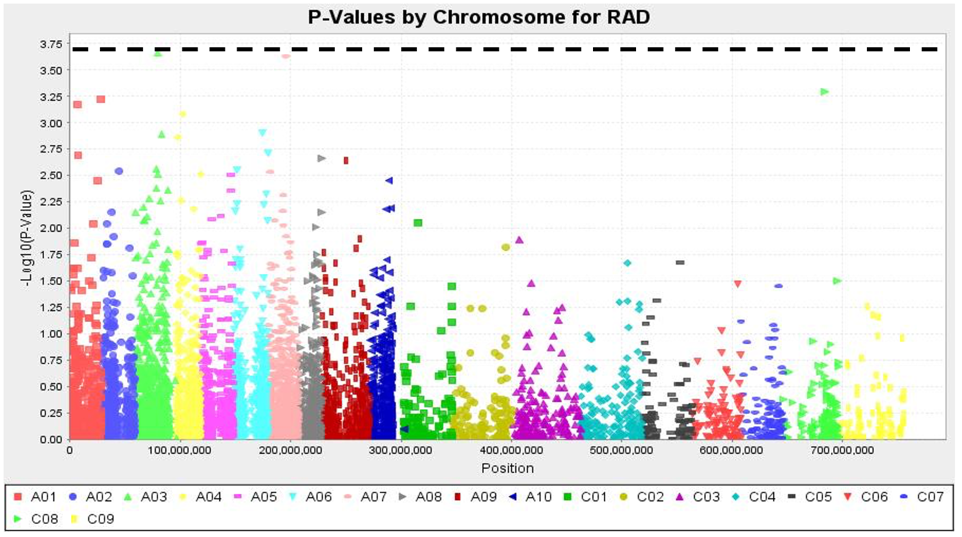

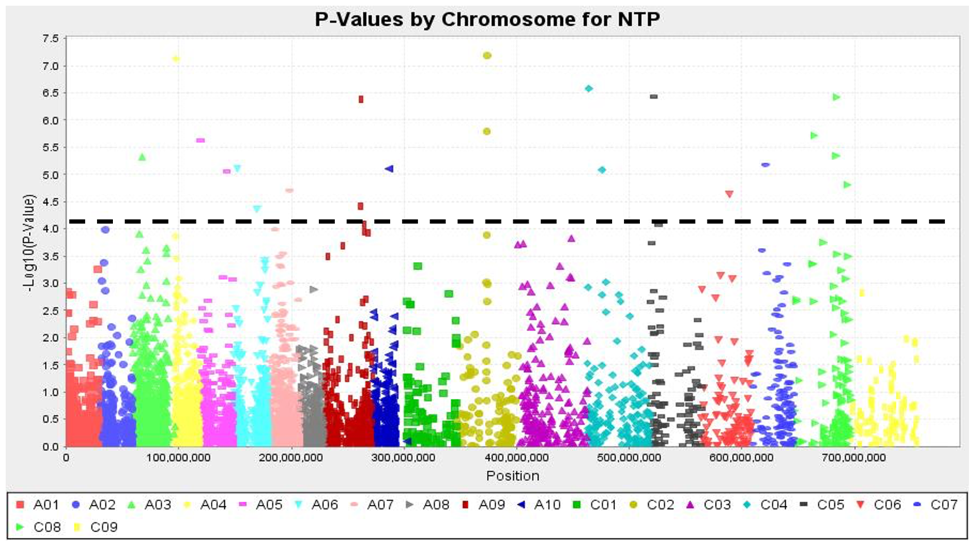
**

**(c)**  **(d)**

**Supplementary Figure 5c.** Manhattan plots of the Q + K MLM models for identifying root architecture traits loci in 313 *Brassica* accessions representing five species *B. napus*, *B. oleracea*, *B. rapa*, *B. carinata*, and *B. juncea*. The dashed horizontal lines indicate the Bonferroni-adjusted significance threshold known as “logarithm-of-odds” (LOD score). The dots above the significance threshold indicate SNPs associated with resistance to each trait.

**
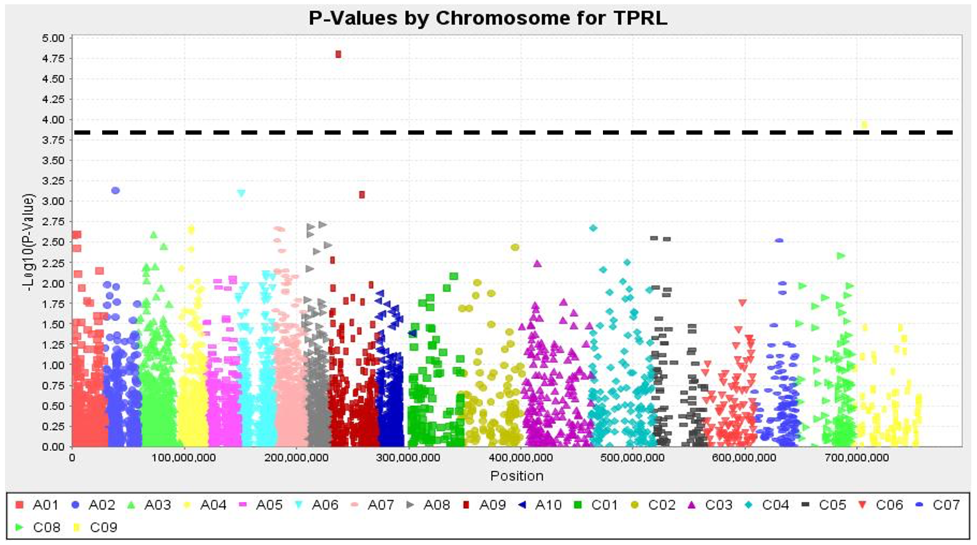
**
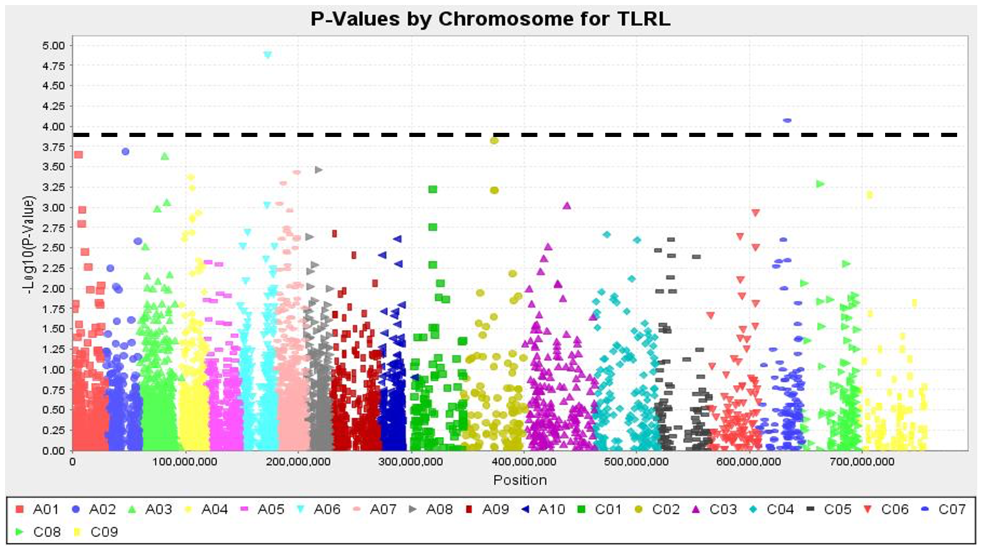


**(e)**  **(f)**

**
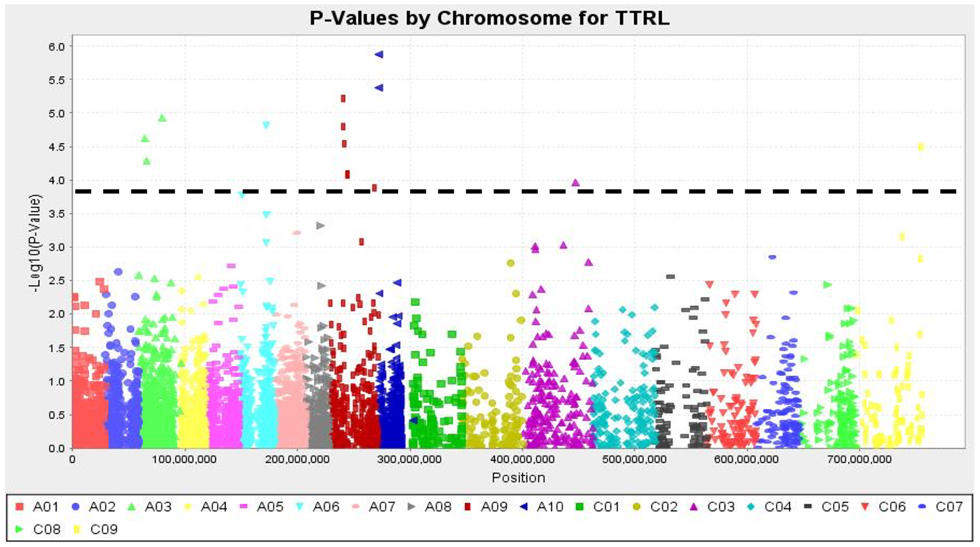

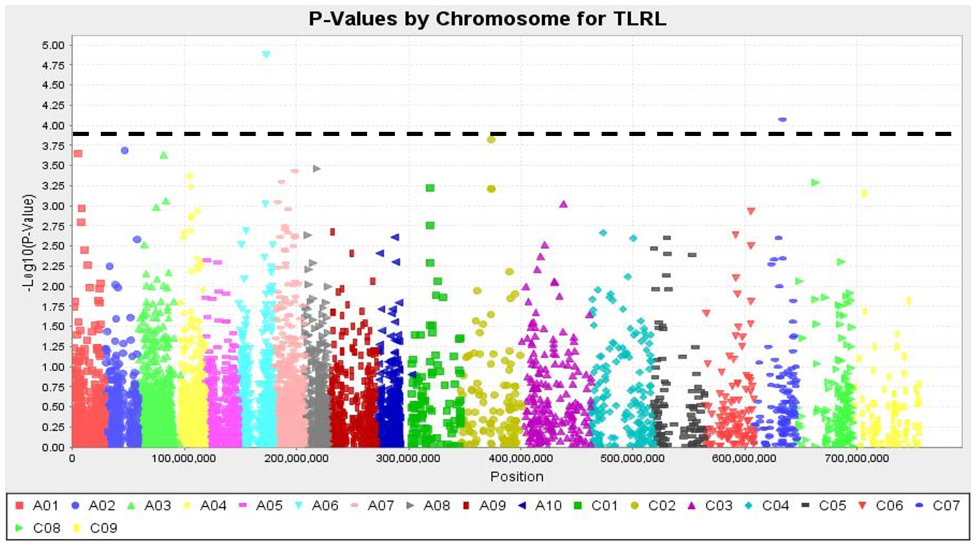
**

**(g)**  **(h)**

**Supplementary Figure 5c (continued).** Manhattan plots of the Q + K MLM models for identifying root architecture traits loci in 313 *Brassica* accessions representing five species *B. napus*, *B. oleracea*, *B. rapa*, *B. carinata*, and *B. juncea*. The dashed horizontal lines indicate the Bonferroni-adjusted significance threshold known as “logarithm-of-odds” (LOD score). The dots above the significance threshold indicate SNPs associated with resistance to each trait.


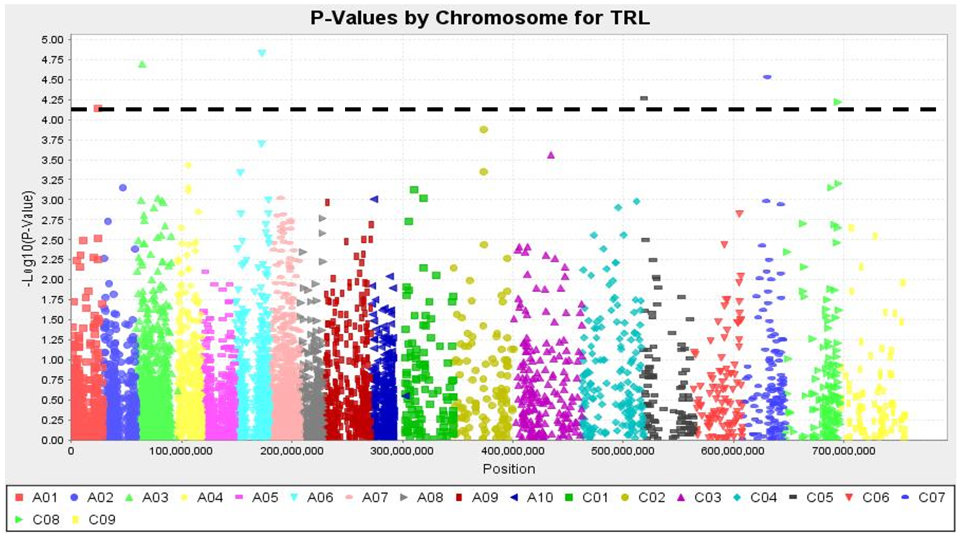

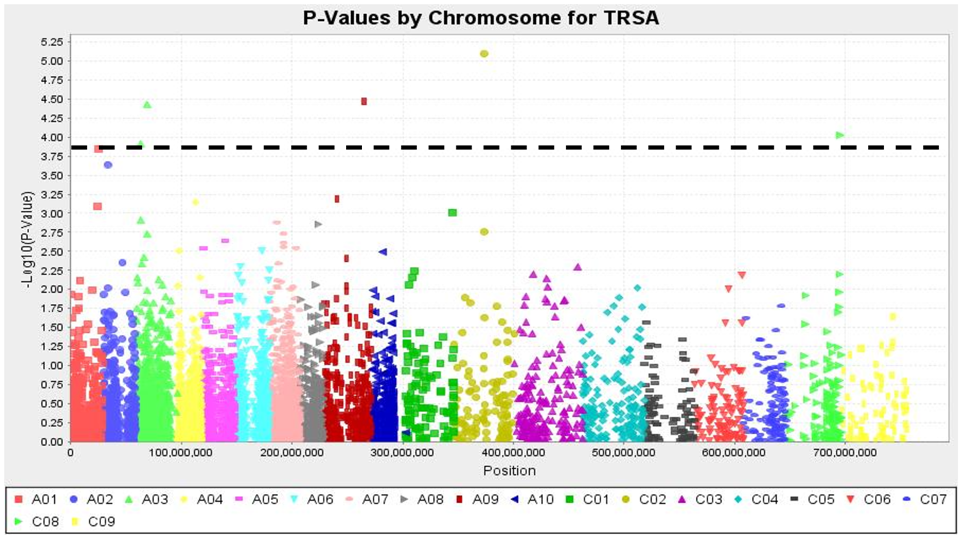


**(a)**  **(b)**

**
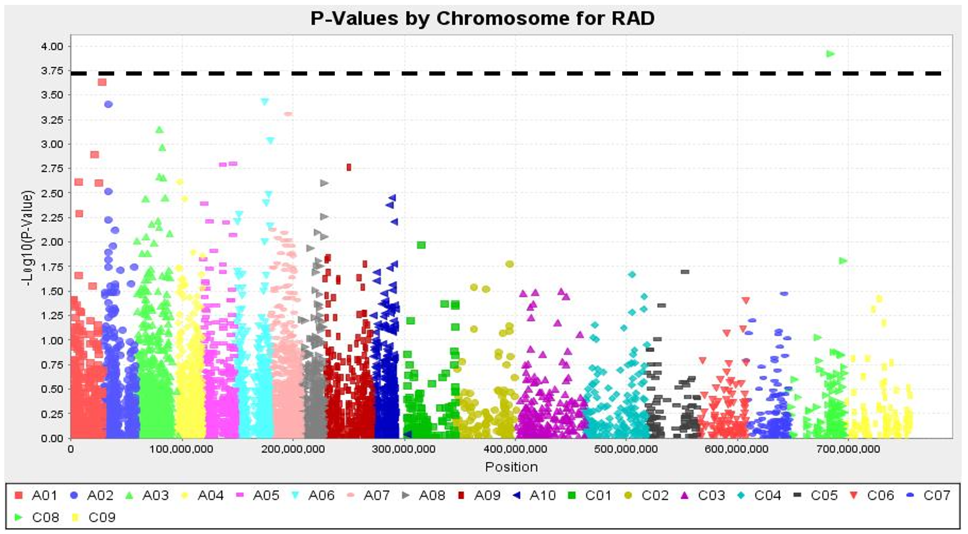

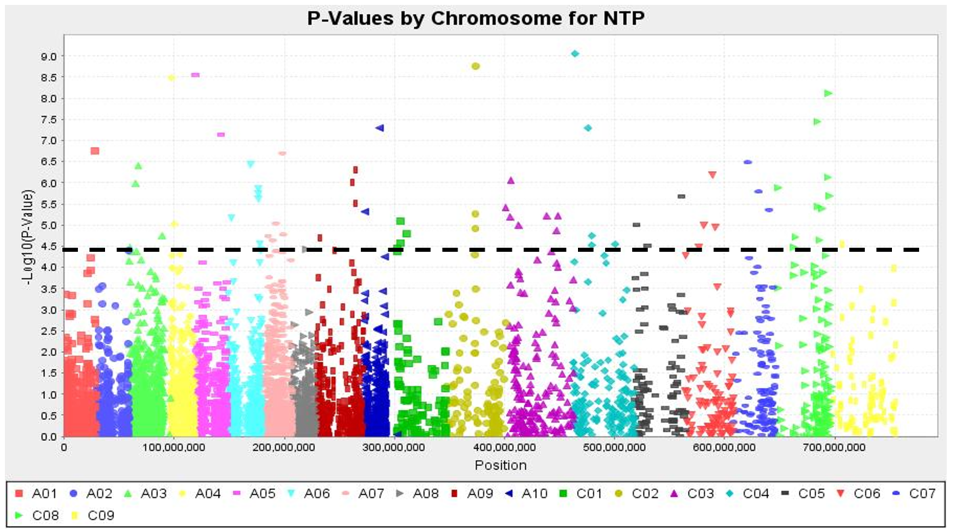
**

**(c)**  **(d)**

**Supplementary Figure 5d.** Manhattan plots of the PCA-only GLM models for identifying root architecture traits loci in 313 *Brassica* accessions representing five species *B. napus*, *B. oleracea*, *B. rapa*, *B. carinata*, and *B. juncea*. The dashed horizontal lines indicate the Bonferroni-adjusted significance threshold known as “logarithm-of-odds” (LOD score). The dots above the significance threshold indicate SNPs associated with resistance to each trait.

**
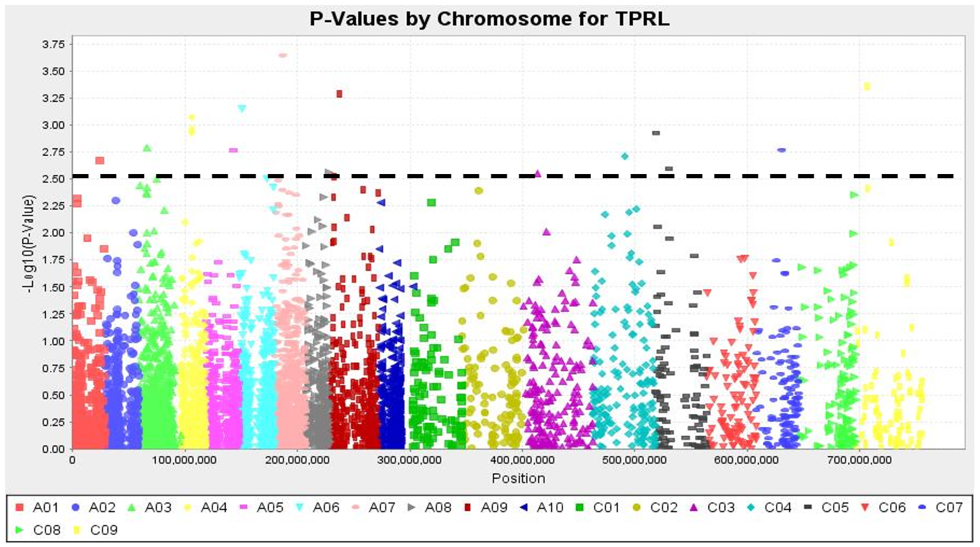
**
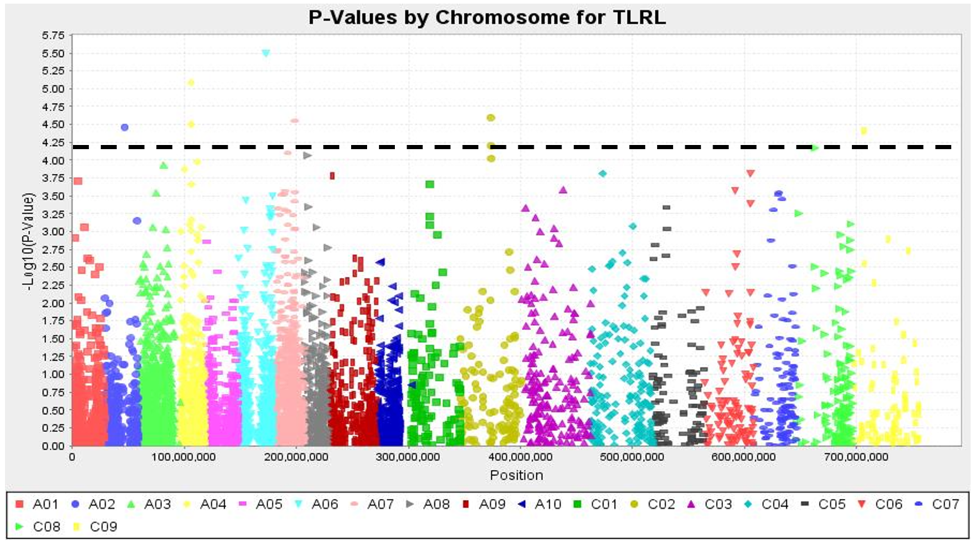


**(e)**  **(f)**


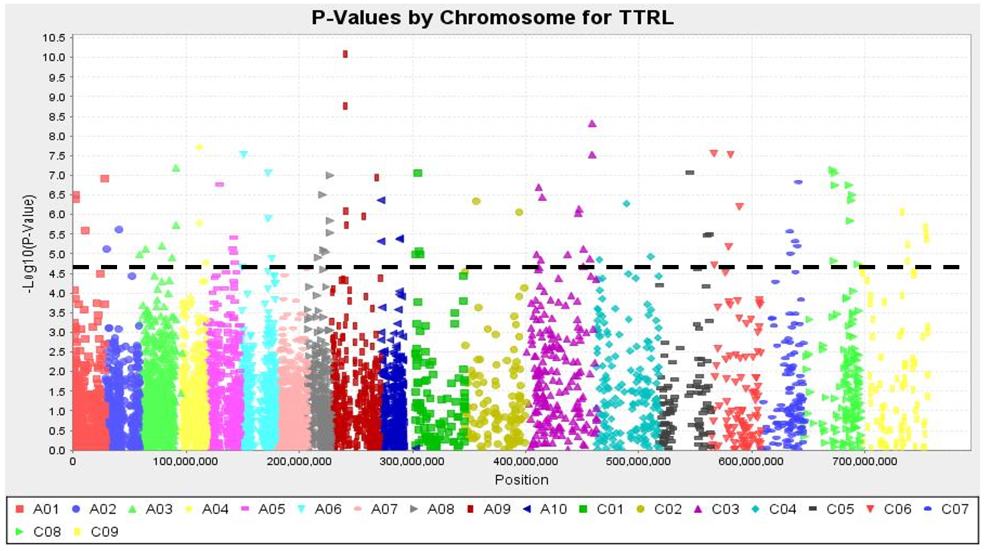
 **
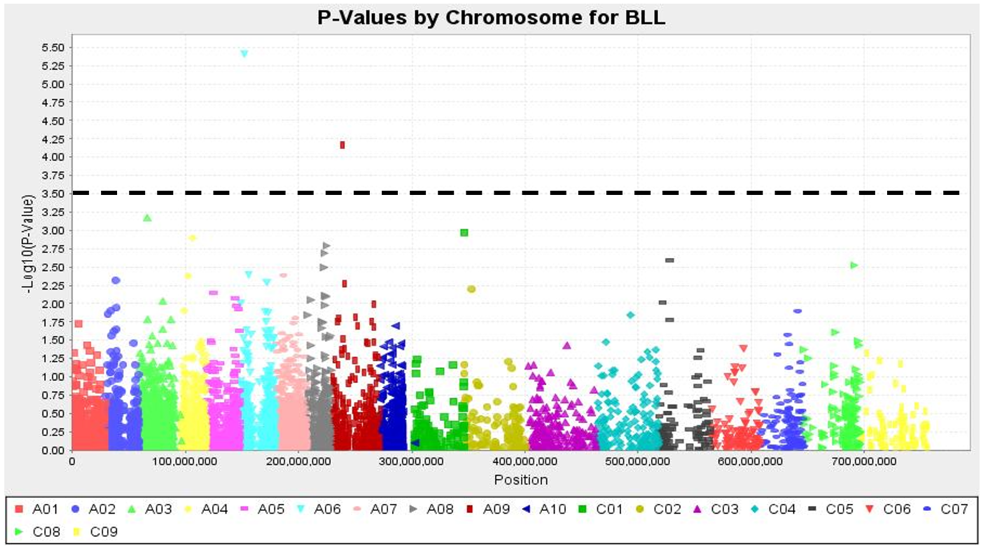
**

**(g)**  **(h)**

**Supplementary Figure 5d (continued).** Manhattan plots of the PCA-only GLM models for identifying root architecture traits loci in 313 *Brassica* accessions representing five species *B. napus*, *B. oleracea*, *B. rapa*, *B. carinata*, and *B. juncea*. The dashed horizontal lines indicate the Bonferroni-adjusted significance threshold known as “logarithm-of-odds” (LOD score). The dots above the significance threshold indicate SNPs associated with resistance to each trait.

**(a)**  **(b)**

**(c)**  **(d)**

**Supplementary Figure 5e.** Manhattan plots of the Q-only GLM models for identifying root architecture traits loci in 313 *Brassica* accessions representing five species *B. napus*, *B. oleracea*, *B. rapa*, *B. carinata*, and *B. juncea*. The dashed horizontal lines indicate the Bonferroni-adjusted significance threshold known as “logarithm-of-odds” (LOD score). The dots above the significance threshold indicate SNPs associated with resistance to each trait.

**(e)**  **(f)**

**(g)**  **(h)**

**Supplementary Figure 5e (continued).** Manhattan plots of the Q-only GLM models for identifying root architecture traits loci in 313 *Brassica* accessions representing five species *B. napus*, *B. oleracea*, *B. rapa*, *B. carinata*, and *B. juncea*. The dashed horizontal lines indicate the Bonferroni-adjusted significance threshold known as “logarithm-of-odds” (LOD score). The dots above the significance threshold indicate SNPs associated with resistance to each trait.

**(a)**  **(b) (c)**

**(d)**  **(e) (f)**

**Supplementary Figure 6.** Plots of correlation coefficient (*r^2^*) and physical distance (in Mb) for SNP markers on chromosomes A01 – A10 (**a-j**) and chromosomes C01 – C09 (**k-s**). The red curves represent the fitted plots of the data points, while the yellow line represents the background linkage disequilibrium (BLD) or threshold line. The decay of linkage disequilibrium was determined by projecting the intersection of the curves and the BLD line onto the physical distance axis.

**(g)**  **(h) (i)**

**(j)**  **(k) (l)**

**Supplementary Figure 6 (continued).** Plots of correlation coefficient (*r^2^*) and physical distance (in Mb) for SNP markers on chromosomes A01 – A10 (**a-j**) and chromosomes C01 – C09 (**k-s**). The red curves represent the fitted plots of the data points, while the yellow line represents the background linkage disequilibrium (BLD) or threshold line. The decay of linkage disequilibrium was determined by projecting the intersection of the curves and the BLD line onto the physical distance axis.

**(m)**  **(n) (o)**

**(p)**  **(q) (r)**

**Supplementary Figure 6 (continued).** Plots of correlation coefficient (*r^2^*) and physical distance (in Mb) for SNP markers on chromosomes A01 – A10 (**a-j**) and chromosomes C01 – C09 (**k-s**). The red curves represent the fitted plots of the data points, while the yellow line represents the background linkage disequilibrium (BLD) or threshold line. The decay of linkage disequilibrium was determined by projecting the intersection of the curves and the BLD line onto the physical distance axis.

**(s)**

**Supplementary Figure 6 (continued).** Plots of correlation coefficient (*r^2^*) and physical distance (in Mb) for SNP markers on chromosomes A01 – A10 (**a-j**) and chromosomes C01 – C09 (**k-s**). The red curves represent the fitted plots of the data points, while the yellow line represents the background linkage disequilibrium (BLD) or threshold line. The decay of linkage disequilibrium was determined by projecting the intersection of the curves and the BLD line onto the physical distance axis.
