# Supplementary material for: Genome-wide association studies of root system architecture traits in a broad collection of Brassica genotypes
Source: Front Plant Sci. 2024 May 28;15:1389082. doi: 10.3389/fpls.2024.1389082 (PMC11165082; doi:10.3389/fpls.2024.1389082)
Supplement: Supplementary file 2 [file DataSheet_2.docx]

Supplementary Material

1. **Supplementary Tables**

**Supplementary Table 1.** List of 379 genotypes of *Brassica* used in the root system architecture studies under semi-hydroponic system. Information about *Brassica* species, genotypes #, root traits including total root length (TRL/cm per plant), total surface area of roots (TRSA/cm^2^ per plant), root average diameter (RAD/cm per plant), number of tips (NTP per plant), total primary root length (TPRL/cm per plant), total lateral root length (TLRL/cm per plant), total tertiary root length (TTRL/cm per plant), basal link length (BLL/cm per plant), and root size of small, medium,and large were provided.

**Supplementary Table 1a.** Commercial lines

| Genotypes # | TRL | TRSA | RAD | NTP | TPRL | TLRL | TTRL | BLL | Root Size |
| --- | --- | --- | --- | --- | --- | --- | --- | --- | --- |
| 5770 | 165.53 | 14.48 | 0.27 | 636.69 | 26.62 | 100.53 | 14.78 | 1.57 | Large |
| 7454 | 280.4 | 33.61 | 0.37 | 1910.06 | 37.21 | 137.97 | 24.48 | 1.3 | Large |
| 08N823R | 81.76 | 6.24 | 0.25 | 173.25 | 18.18 | 53.63 | 6.32 | 1.16 | Small |
| 45CM39 | 288.97 | 34.49 | 0.38 | 1682.5 | 27.61 | 141.05 | 46.37 | 1.37 | Large |
| 45CS40 | 175.99 | 13.24 | 0.25 | 280.88 | 30.25 | 114.94 | 20.18 | 1.19 | Large |
| 45H26 | 212.85 | 21.63 | 0.32 | 806.75 | 31.1 | 131.74 | 20.33 | 1.27 | Large |
| 45H29 | 175.1 | 18.92 | 0.34 | 1166.44 | 20.62 | 75.51 | 25.8 | 1.32 | Large |
| 45H31 | 183.89 | 17.42 | 0.29 | 517.63 | 27.48 | 115.5 | 22.3 | 1.59 | Large |
| 45M35 | 296.5 | 39.44 | 0.42 | 1131.75 | 39.59 | 171.69 | 37.68 | 1.28 | Large |
| 6207TF | 126.84 | 8.79 | 0.22 | 197.56 | 27.27 | 92.84 | 6.61 | 1.56 | Medium |
| 6056CR | 190.34 | 14.03 | 0.23 | 384.38 | 31.24 | 124.84 | 23.53 | 1.4 | Large |
| 7444BL | 349.98 | 43.65 | 0.39 | 1529.88 | 38.27 | 228.04 | 22.55 | 1.48 | Large |
| 9558C | 206.9 | 26.49 | 0.4 | 868.06 | 32.27 | 122.62 | 21.15 | 1.75 | Large |
| Brevant3010 | 279.24 | 35.98 | 0.41 | 1295.25 | 33.37 | 154.5 | 30.77 | 1.86 | Large |
| Brutor | 301.55 | 41.72 | 0.44 | 930.63 | 38.27 | 162.06 | 57.19 | 1.79 | Large |
| BY6204 | 253.26 | 17.98 | 0.23 | 428.69 | 36.69 | 162.75 | 41.71 | 1.37 | Large |
| CS2000 | 324.8 | 31.43 | 0.3 | 868.63 | 39.6 | 224.39 | 36.18 | 1.39 | Large |
| CS2600 | 252.57 | 29.86 | 0.38 | 1491.5 | 34.25 | 141.72 | 12 | 1.38 | Large |
| D3155C | 242.63 | 26.1 | 0.32 | 1693.81 | 30.22 | 118.07 | 28.95 | 1.47 | Large |
| DKTF98CR | 266.07 | 19.58 | 0.23 | 484.19 | 42.11 | 189.29 | 22.4 | 1.96 | Large |
| L150 | 192.27 | 22.43 | 0.37 | 1280.13 | 31.51 | 89.51 | 5.63 | 1.75 | Large |
| L234PC | 148.98 | 18.41 | 0.39 | 929.94 | 23.3 | 76.91 | 7.94 | 1.39 | Large |
| L241 | 289.32 | 32.91 | 0.35 | 1202.69 | 37.18 | 167.02 | 39.67 | 1.89 | Large |
| L255PC | 272.69 | 19.5 | 0.23 | 491.44 | 35.91 | 180.24 | 36.46 | 1.47 | Large |
| L343PC | 224.92 | 15.16 | 0.21 | 525.75 | 37.14 | 152.95 | 22.05 | 1.65 | Large |
| L345PC | 338.73 | 36.79 | 0.35 | 2753.19 | 36.9 | 159.49 | 16.5 | 2.11 | Large |
| Laurentian | 170.28 | 10.94 | 0.2 | 628.25 | 30.99 | 104.21 | 22.34 | 1.2 | Large |
| Mendel | 388.51 | 44.36 | 0.36 | 1622.38 | 42.4 | 249.93 | 30.95 | 1.76 | Large |
| P501L | 414.51 | 52.98 | 0.41 | 1403.19 | 43.48 | 247.38 | 56.92 | 1.53 | Large |
| WESTAR | 161.06 | 29.86 | 0.59 | 285.44 | 25 | 90.19 | 24.09 | 2.22 | Large |

**Supplementary Table 1b.** *Brassica napus*

| Genotypes # | TRL | TRSA | RAD | NTP | TPRL | TLRL | TTRL | BLL | Root Size |
| --- | --- | --- | --- | --- | --- | --- | --- | --- | --- |
| ECD06 | 204.34 | 20.97 | 0.31 | 778.88 | 28.77 | 130.5 | 13.14 | 1.29 | Large |
| ECD08 | 242.64 | 23.96 | 0.3 | 1074.44 | 34.15 | 143.03 | 26.93 | 1.44 | Large |
| ECD09 | 186.3 | 12.88 | 0.22 | 399 | 34.64 | 135.9 | 7.73 | 1.61 | Large |
| ECD10 | 223.13 | 25.17 | 0.36 | 1578.56 | 33.08 | 107.93 | 11.48 | 1.82 | Large |
| FG001 | 131.86 | 12.67 | 0.29 | 164.38 | 22.52 | 77.33 | 32.01 | 2.71 | Large |
| FG002 | 310.25 | 28.53 | 0.28 | 419.88 | 36.85 | 169.8 | 103.6 | 1.81 | Large |
| FG004 | 266.6 | 23.98 | 0.26 | 403.81 | 38.28 | 151.1 | 77.21 | 1.85 | Large |
| FG005 | 180.68 | 17.37 | 0.28 | 233.81 | 27.39 | 104.69 | 48.6 | 2.12 | Large |
| FG007 | 284.23 | 27.62 | 0.28 | 971.13 | 46.73 | 152.63 | 84.87 | 2.08 | Large |
| FG008 | 173.7 | 17.01 | 0.28 | 522.81 | 32.9 | 97.05 | 43.76 | 2.93 | Large |
| FG009 | 47.98 | 10.58 | 0.56 | 246.31 | 12.05 | 29.27 | 6.66 | 1.47 | Small |
| FG010 | 161.1 | 14.39 | 0.28 | 215.81 | 28.62 | 94.14 | 38.34 | 2.33 | Large |
| FG011 | 141.59 | 15.11 | 0.3 | 248.06 | 20.19 | 82.13 | 39.27 | 2.45 | Large |
| FG012 | 161.13 | 15.61 | 0.29 | 470.69 | 26.94 | 90.2 | 43.98 | 2.2 | Large |
| FG013 | 159.33 | 14.22 | 0.25 | 308.69 | 27.93 | 93.17 | 38.23 | 1.81 | Large |
| FG014 | 153.67 | 14.52 | 0.28 | 250.38 | 25.4 | 89.56 | 38.71 | 2.6 | Large |
| FG015 | 223.39 | 21.92 | 0.32 | 487.25 | 25.07 | 143.4 | 54.93 | 2.21 | Large |
| FG019 | 156.35 | 14.72 | 0.27 | 378.13 | 26.82 | 90.21 | 39.32 | 2.19 | Large |
| FG022 | 103.71 | 10.35 | 0.28 | 447.06 | 16.5 | 58.03 | 29.19 | 2.06 | Small |
| FG025 | 164.63 | 17 | 0.3 | 339.88 | 26.56 | 125.97 | 12.11 | 2.82 | Large |
| FG026 | 158.62 | 32.55 | 0.57 | 418.56 | 27.66 | 116.55 | 14.41 | 1.9 | Large |
| FG027 | 53.28 | 13.44 | 0.54 | 453.38 | 19.2 | 31.41 | 2.67 | 1.48 | Small |
| FG029 | 222.22 | 44.42 | 0.58 | 321.88 | 37.74 | 167.08 | 17.39 | 3.07 | Large |
| FG031 | 134.66 | 12.66 | 0.28 | 283.69 | 20.67 | 80.41 | 33.58 | 1.89 | Large |
| FG033 | 147.46 | 22.31 | 0.39 | 468.19 | 27.66 | 80.94 | 38.86 | 1.73 | Large |
| FG034 | 100.38 | 22.4 | 0.65 | 236.25 | 18.71 | 73.52 | 8.16 | 2.43 | Small |
| FG035 | 96.01 | 9.72 | 0.3 | 191.06 | 15.57 | 64.57 | 15.87 | 1.62 | Small |
| FG037 | 96.78 | 10.1 | 0.31 | 183.69 | 18.97 | 57.33 | 20.47 | 1.99 | Small |
| FG038 | 61.31 | 6.04 | 0.31 | 94.25 | 13.02 | 36.99 | 11.3 | 1.39 | Small |
| FG040 | 135.85 | 12.33 | 0.28 | 237.19 | 23.29 | 81.95 | 30.61 | 2.17 | Large |
| FG041 | 116.1 | 13.85 | 0.34 | 447.88 | 19.64 | 79.24 | 17.23 | 2.11 | Medium |
| FG042 | 127.31 | 24.53 | 0.48 | 357.19 | 15.86 | 80.73 | 30.72 | 0.86 | Medium |
| FG665 | 178.79 | 17.9 | 0.31 | 282.56 | 20.54 | 75.36 | 82.89 | 1.5 | Large |
| FG666 | 224.02 | 22.39 | 0.28 | 598.69 | 35.68 | 102.94 | 85.4 | 1.64 | Large |
| FG667 | 191.03 | 20.04 | 0.31 | 469.5 | 23.73 | 92.2 | 75.1 | 1.45 | Large |
| FG668 | 177.86 | 23.55 | 0.41 | 306.25 | 24.11 | 88.6 | 65.15 | 1.38 | Large |

**Supplementary Table 1b (continued).** *Brassica napus*

| Genotypes # | TRL | TRSA | RAD | NTP | TPRL | TLRL | TTRL | BLL | Root Size |
| --- | --- | --- | --- | --- | --- | --- | --- | --- | --- |
| FG670 | 276.35 | 24.48 | 0.26 | 308.31 | 36.58 | 143.35 | 96.42 | 1.8 | Large |
| FG688 | 284.28 | 29.98 | 0.3 | 505.25 | 33.66 | 142.79 | 107.83 | 1.65 | Large |
| FG689 | 249.48 | 25.99 | 0.3 | 606.69 | 23.91 | 102.4 | 123.17 | 1.74 | Large |
| FG690 | 243.24 | 27.83 | 0.32 | 531.5 | 27.71 | 104.97 | 110.56 | 1.67 | Large |
| FG691 | 166.12 | 19.37 | 0.33 | 304.5 | 26.94 | 94.99 | 44.19 | 1.75 | Large |
| FG692 | 278.65 | 32.4 | 0.33 | 929.19 | 31.72 | 135.14 | 111.79 | 1.63 | Large |
| FG694 | 268.12 | 26.84 | 0.29 | 651.63 | 32.43 | 132.36 | 103.33 | 1.71 | Large |
| FG710 | 231.66 | 22.7 | 0.29 | 523.44 | 30.76 | 110.06 | 90.85 | 1.74 | Large |
| FG723 | 176.26 | 17.54 | 0.29 | 494.94 | 26.97 | 97.65 | 51.64 | 1.85 | Large |
| FG725 | 280.6 | 33.07 | 0.33 | 803.94 | 32.74 | 139.29 | 108.57 | 1.86 | Large |
| FG726 | 252.92 | 30.77 | 0.34 | 748.63 | 35.31 | 137.66 | 79.95 | 2.13 | Large |
| FG727 | 273 | 30.87 | 0.32 | 412.69 | 37.33 | 136.31 | 99.37 | 1.86 | Large |
| FG730 | 269.25 | 24.71 | 0.27 | 371.56 | 33.72 | 139.71 | 95.82 | 1.6 | Large |
| FG734 | 173.16 | 19.46 | 0.31 | 426.69 | 30.41 | 97.56 | 45.19 | 1.23 | Large |
| FG735 | 278.41 | 24.76 | 0.27 | 432.69 | 34.57 | 145.98 | 97.86 | 1.65 | Large |
| FG736 | 154.09 | 15.49 | 0.28 | 546.88 | 29.45 | 94.07 | 30.58 | 1.81 | Large |
| FG737 | 159.53 | 16.68 | 0.29 | 259.44 | 29.54 | 88.4 | 41.59 | 1.7 | Large |
| FG748 | 164.64 | 14.75 | 0.27 | 252.38 | 30.5 | 86.41 | 47.73 | 2.32 | Large |
| FG750 | 177.34 | 16.43 | 0.26 | 448.69 | 31.39 | 94.04 | 51.91 | 2.33 | Large |
| FG756 | 150.77 | 15.04 | 0.28 | 492.63 | 20.27 | 82.44 | 48.06 | 1.98 | Large |
| FG767 | 201.13 | 19.23 | 0.29 | 380.44 | 28.22 | 109.94 | 62.98 | 1.8 | Large |
| FG768 | 233.09 | 21.24 | 0.27 | 203.44 | 30.61 | 122.96 | 79.53 | 1.7 | Large |
| FG769 | 182.09 | 18.46 | 0.28 | 379.44 | 26.56 | 94.82 | 60.71 | 2.08 | Large |
| FG771 | 159.76 | 25.76 | 0.41 | 489.88 | 23.9 | 81.4 | 54.47 | 1.45 | Large |
| FG782 | 129.84 | 13.93 | 0.31 | 462.75 | 25.78 | 63.65 | 40.41 | 1.84 | Medium |
| FG783 | 187.66 | 19.74 | 0.29 | 390.5 | 29.25 | 94.98 | 63.44 | 1.76 | Large |
| FG784 | 164.57 | 15.24 | 0.28 | 294.19 | 29.75 | 85.32 | 49.51 | 1.53 | Large |
| FG818 | 182.81 | 18.4 | 0.29 | 300.31 | 33.38 | 94.58 | 54.85 | 1.71 | Large |
| FG819 | 131.47 | 13.87 | 0.31 | 390 | 25.89 | 72.02 | 33.55 | 1.41 | Large |
| FG820 | 125.24 | 13.28 | 0.31 | 369.63 | 21.78 | 66.43 | 37.03 | 1.29 | Medium |
| FG821 | 198.5 | 19.93 | 0.29 | 575.69 | 25.19 | 103.51 | 69.8 | 1.7 | Large |
| FG822 | 209.74 | 21.12 | 0.3 | 385.13 | 27.32 | 117.39 | 65.02 | 1.51 | Large |
| FG823 | 185.22 | 21.9 | 0.34 | 265.24 | 29.04 | 106.48 | 49.7 | 1.93 | Large |
| FG825 | 200.94 | 22.03 | 0.32 | 227.6 | 32.38 | 110.28 | 58.28 | 1.92 | Large |
| FG826 | 211.97 | 21.81 | 0.32 | 476.63 | 26.47 | 110.86 | 74.65 | 1.8 | Large |
| FG828 | 201.7 | 23.46 | 0.33 | 342.63 | 28.19 | 111 | 62.5 | 1.66 | Large |

**Supplementary Table 1c.** *Brassica rapa*

| Genotypes # | TRL | TRSA | RAD | NTP | TPRL | TLRL | TTRL | BLL | Root Size |
| --- | --- | --- | --- | --- | --- | --- | --- | --- | --- |
| ECD02 | 174.92 | 20.7 | 0.38 | 1097 | 27.52 | 97.55 | 6.17 | 1.7 | Large |
| ECD05 | 147.98 | 9.97 | 0.21 | 405.94 | 27.06 | 96.48 | 14.43 | 1.64 | Large |
| FG043 | 113.87 | 9.72 | 0.25 | 252.44 | 19.65 | 82.21 | 12.01 | 1.94 | Small |
| FG044 | 166.5 | 15.3 | 0.26 | 442.06 | 22.52 | 106.54 | 37.45 | 1.36 | Large |
| FG052 | 75.45 | 15.64 | 0.61 | 147 | 14.98 | 53.26 | 7.2 | 1.79 | Small |
| FG053 | 88.03 | 8.31 | 0.29 | 260.38 | 13.68 | 65.6 | 8.76 | 2.44 | Small |
| FG054 | 94.52 | 16.01 | 0.48 | 325.38 | 17.79 | 63.16 | 13.57 | 2.59 | Small |
| FG056 | 205.9 | 44.49 | 0.55 | 759.19 | 31.62 | 109.91 | 64.37 | 1.6 | Large |
| FG058 | 151.86 | 32.93 | 0.58 | 461.19 | 23.57 | 101.99 | 26.29 | 2.16 | Large |
| FG060 | 72.82 | 6.3 | 0.25 | 234.56 | 15.77 | 52.67 | 4.37 | 1.56 | Small |
| FG061 | 143.48 | 15.29 | 0.28 | 557.56 | 20.88 | 80.11 | 42.5 | 1.18 | Large |
| FG062 | 83.92 | 7.29 | 0.26 | 193.25 | 15.69 | 60.74 | 7.48 | 1.81 | Small |
| FG063 | 110.87 | 23.69 | 0.58 | 316.38 | 19.73 | 71.47 | 19.66 | 1.37 | Small |
| FG066 | 118.03 | 10.11 | 0.24 | 269.56 | 20.99 | 86.41 | 10.63 | 2.14 | Medium |
| FG072 | 139.42 | 12.18 | 0.26 | 342.94 | 25.39 | 101.72 | 12.31 | 1.96 | Large |
| FG073 | 109.64 | 11.61 | 0.29 | 419.81 | 24.38 | 76.53 | 8.73 | 1.71 | Small |
| FG080 | 115.55 | 20.74 | 0.48 | 649.44 | 20.36 | 77.11 | 18.08 | 2.01 | Medium |
| FG082 | 105.39 | 12.44 | 0.34 | 287.88 | 17.31 | 55.36 | 32.72 | 1.13 | Small |
| FG084 | 66.91 | 5.02 | 0.22 | 186 | 13.35 | 43.64 | 9.93 | 1.55 | Small |
| FG085 | 104.04 | 8.93 | 0.26 | 365.5 | 17.11 | 71.8 | 15.13 | 1.66 | Small |
| FG088 | 138.54 | 13.39 | 0.28 | 343.63 | 23.45 | 95.9 | 19.19 | 2.08 | Large |
| FG091 | 89.96 | 17.36 | 0.57 | 186.56 | 22.76 | 61.81 | 5.4 | 1.76 | Small |
| FG092 | 157.98 | 31.27 | 0.51 | 722 | 26.76 | 82.61 | 48.61 | 1.18 | Large |
| FG094 | 109.56 | 17.33 | 0.45 | 223.25 | 25.24 | 73.82 | 10.5 | 1.94 | Small |
| FG095 | 92.76 | 9.27 | 0.29 | 331.44 | 21.25 | 61.8 | 9.71 | 1.47 | Small |
| FG096 | 203.71 | 20.53 | 0.26 | 840.31 | 25.12 | 111.09 | 67.51 | 1.64 | Large |
| FG097 | 121.22 | 11.3 | 0.27 | 335.81 | 18.99 | 80.11 | 22.12 | 1.46 | Medium |
| FG101 | 129.21 | 11.33 | 0.26 | 403.44 | 16.87 | 87.91 | 24.43 | 1.62 | Medium |
| FG102 | 109.04 | 14.46 | 0.41 | 275.75 | 20.59 | 68.79 | 19.66 | 1.46 | Small |
| FG106 | 164.41 | 12.02 | 0.26 | 339.56 | 21.55 | 118.94 | 23.92 | 1.49 | Large |
| FG109 | 128.25 | 11.69 | 0.26 | 278.69 | 23.03 | 93.22 | 11.99 | 1.74 | Medium |
| FG112 | 103.9 | 13.74 | 0.35 | 147.13 | 19.29 | 64.45 | 20.16 | 1.54 | Small |
| FG113 | 67.59 | 10.51 | 0.4 | 247.56 | 17.91 | 39.86 | 9.82 | 1.54 | Small |
| FG114 | 153.37 | 15.67 | 0.29 | 405.94 | 25.85 | 90.88 | 36.65 | 1.81 | Large |
| FG119 | 86.01 | 6.32 | 0.23 | 160.5 | 16.37 | 58.19 | 11.45 | 1.32 | Small |
| FG120 | 161.78 | 16.37 | 0.29 | 289.5 | 32.1 | 119.65 | 10.03 | 1.7 | Large |

**Supplementary Table 1c (continued).** *Brassica rapa*

| Genotypes # | TRL | TRSA | RAD | NTP | TPRL | TLRL | TTRL | BLL | Root Size |
| --- | --- | --- | --- | --- | --- | --- | --- | --- | --- |
| FG121 | 149.89 | 12.28 | 0.23 | 367.38 | 23.97 | 103.84 | 22.08 | 1.55 | Large |
| FG123 | 63.78 | 14.48 | 0.58 | 309.63 | 18.35 | 40.72 | 4.72 | 2.35 | Small |
| FG124 | 118.27 | 30.03 | 0.55 | 817.69 | 26.84 | 81.1 | 10.33 | 2.01 | Medium |
| FG125 | 85.49 | 9.11 | 0.3 | 362.13 | 22.37 | 55.76 | 7.36 | 1.61 | Small |
| FG126 | 147.77 | 12.28 | 0.25 | 327.63 | 27 | 105.48 | 15.28 | 2.06 | Large |
| FG129 | 125.27 | 27.7 | 0.58 | 418.25 | 23.8 | 68.81 | 32.66 | 1.53 | Medium |
| FG133 | 51.39 | 5.03 | 0.28 | 210.13 | 12.63 | 34.64 | 4.13 | 2.11 | Small |
| FG136 | 75.44 | 7.08 | 0.28 | 187.44 | 16.75 | 53.81 | 4.88 | 1.69 | Small |
| FG137 | 87.33 | 13.89 | 0.42 | 276 | 18.42 | 54.43 | 14.49 | 1.43 | Small |
| FG138 | 158.21 | 17.2 | 0.32 | 459.31 | 27.07 | 107.23 | 23.92 | 2.18 | Large |
| FG142 | 79.64 | 13.31 | 0.41 | 452.31 | 14.59 | 49.48 | 15.57 | 1.1 | Small |
| FG153 | 56.62 | 13.05 | 0.61 | 202.94 | 16.29 | 31.57 | 8.76 | 1.89 | Small |
| FG158 | 85.38 | 8.35 | 0.27 | 396.44 | 20.64 | 57.56 | 7.18 | 1.28 | Small |
| FG166 | 98.67 | 9.63 | 0.25 | 554 | 20.81 | 62.59 | 15.28 | 1.3 | Small |
| FG167 | 108.01 | 10.95 | 0.28 | 302.06 | 16.17 | 79.98 | 11.86 | 1.89 | Small |
| FG183 | 158.16 | 33.32 | 0.57 | 480.88 | 28.15 | 98.18 | 31.83 | 1.9 | Large |
| FG184 | 108.66 | 22.76 | 0.59 | 226.75 | 20.06 | 65.4 | 23.2 | 1.22 | Small |
| FG191 | 144.75 | 14.08 | 0.27 | 493.25 | 23.07 | 93.21 | 28.48 | 1.41 | Large |
| FG194 | 130.88 | 12.85 | 0.28 | 460.25 | 22.5 | 87.28 | 21.1 | 1.88 | Large |
| FG196 | 144.51 | 18.39 | 0.33 | 386 | 18.05 | 94.05 | 32.4 | 1.81 | Large |
| FG201 | 175.72 | 22.76 | 0.33 | 782.88 | 29.34 | 105.04 | 41.33 | 1.29 | Large |
| FG203 | 66.23 | 6.46 | 0.3 | 184.88 | 15.01 | 40.99 | 10.23 | 1.31 | Small |
| FG211 | 214.14 | 46.48 | 0.63 | 366.19 | 28.23 | 126.37 | 59.54 | 2.08 | Large |
| FG212 | 138.76 | 14.51 | 0.31 | 412.75 | 17.68 | 99.78 | 21.31 | 2.13 | Large |
| FG215 | 139.6 | 30.51 | 0.63 | 250.81 | 28.46 | 78.83 | 32.31 | 1.43 | Large |
| FG219 | 147.57 | 13.13 | 0.27 | 274.94 | 25.86 | 91.62 | 30.09 | 1.38 | Large |

**Supplementary Table 1d.** *Brassica juncea*

| Genotypes # | TRL | TRSA | RAD | NTP | TPRL | TLRL | TTRL | BLL | Root Size |
| --- | --- | --- | --- | --- | --- | --- | --- | --- | --- |
| FG402 | 174.76 | 18.63 | 0.3 | 714 | 27.66 | 126.23 | 20.86 | 2.63 | Large |
| FG403 | 198.41 | 21.12 | 0.29 | 775.13 | 34.91 | 148.31 | 15.19 | 3.48 | Large |
| FG404 | 58.61 | 8.15 | 0.38 | 68.19 | 19.87 | 35.91 | 2.83 | 2.29 | Small |
| FG405 | 76.41 | 10.75 | 0.4 | 78.63 | 23.69 | 47.44 | 5.28 | 1.54 | Small |
| FG406 | 233.97 | 24.38 | 0.31 | 768.31 | 33.62 | 176.7 | 23.66 | 2.56 | Large |
| FG412 | 170.35 | 23.16 | 0.39 | 154.75 | 39.47 | 117.94 | 12.93 | 2.52 | Large |
| FG429 | 106.15 | 15.68 | 0.42 | 114.94 | 22.9 | 74 | 9.26 | 2.23 | Small |
| FG430 | 71.05 | 9.16 | 0.38 | 210.63 | 15.85 | 29.81 | 25.38 | 1.65 | Small |
| FG431 | 103.13 | 13.66 | 0.35 | 586.94 | 22.12 | 43.05 | 37.96 | 2.49 | Small |
| FG432 | 57.74 | 7.22 | 0.32 | 258.06 | 20.37 | 26.75 | 10.62 | 0.99 | Small |
| FG443 | 44.11 | 6.38 | 0.33 | 278.19 | 18.7 | 18.56 | 6.85 | 1.15 | Small |
| FG444 | 99.48 | 10.76 | 0.32 | 199.94 | 27.67 | 45.15 | 26.66 | 1.19 | Small |
| FG449 | 122.98 | 14.02 | 0.33 | 226.38 | 26.08 | 55.45 | 41.45 | 1.88 | Medium |
| FG450 | 136.92 | 15.37 | 0.31 | 357.75 | 30.11 | 61.41 | 45.4 | 2.11 | Large |
| FG454 | 178.07 | 17.59 | 0.32 | 299.69 | 28.06 | 78.87 | 71.13 | 2.04 | Large |
| FG460 | 147.49 | 17.06 | 0.34 | 232.19 | 31.54 | 72.65 | 43.3 | 1.77 | Large |
| FG995 | 127.34 | 13.62 | 0.34 | 294 | 26.48 | 92.83 | 8.03 | 2.04 | Medium |
| FG1003 | 234.13 | 23.53 | 0.3 | 674 | 39.3 | 178.21 | 16.63 | 2.61 | Large |
| FG1005 | 107.87 | 12.15 | 0.33 | 414.63 | 22.14 | 74.43 | 11.3 | 1.8 | Small |
| FG1006 | 49.69 | 5.55 | 0.32 | 219.94 | 14.17 | 31.82 | 3.7 | 2.41 | Small |
| FG1007 | 99.57 | 10.39 | 0.31 | 432.81 | 22.05 | 71.14 | 6.39 | 1.92 | Small |
| FG1023 | 92.31 | 21.02 | 0.55 | 447.25 | 18.73 | 66.12 | 7.46 | 2.52 | Small |
| FG1036 | 35.07 | 4.43 | 0.39 | 19.94 | 12.89 | 21.54 | 0.63 | 1.5 | Small |
| FG1037 | 35.35 | 4.49 | 0.37 | 38.13 | 15.67 | 18.07 | 1.62 | 1.2 | Small |
| FG1039 | 98.08 | 10.06 | 0.28 | 474.75 | 22.69 | 69.41 | 5.99 | 1.4 | Small |
| FG1040 | 86.48 | 20.74 | 0.58 | 464.81 | 19.73 | 53.28 | 13.48 | 2.28 | Small |
| FG1041 | 53.47 | 12.37 | 0.65 | 136.06 | 21.58 | 30.89 | 1 | 1.66 | Small |
| FG1042 | 40.67 | 3.7 | 0.27 | 99.44 | 14.66 | 25.1 | 0.9 | 1.89 | Small |
| FG1043 | 110.83 | 10.55 | 0.29 | 241.44 | 25.34 | 82.1 | 3.38 | 2 | Small |
| FG1049 | 52.21 | 7.04 | 0.34 | 380.63 | 15.03 | 28.61 | 8.57 | 0.98 | Small |
| FG1050 | 59.21 | 14.28 | 0.59 | 362.56 | 16.98 | 34.2 | 8.03 | 1.21 | Small |
| FG1051 | 65.16 | 7.46 | 0.32 | 280.06 | 21.34 | 37.01 | 6.81 | 1.54 | Small |
| FG1053 | 50.01 | 5.12 | 0.33 | 115.38 | 14.7 | 28.58 | 6.72 | 0.99 | Small |
| FG1054 | 50.28 | 12.8 | 0.65 | 252.25 | 17.36 | 26.96 | 5.96 | 1.14 | Small |
| FG1055 | 76.63 | 19.07 | 0.75 | 121.38 | 28.59 | 40.38 | 7.67 | 1.04 | Small |
| FG1056 | 42.9 | 11.34 | 0.57 | 325.56 | 15.25 | 21.98 | 5.66 | 1.17 | Small |

**Supplementary Table 1d (continued).** *Brassica juncea*

| Genotypes # | TRL | TRSA | RAD | NTP | TPRL | TLRL | TTRL | BLL | Root Size |
| --- | --- | --- | --- | --- | --- | --- | --- | --- | --- |
| FG1057 | 139.24 | 15.87 | 0.33 | 470.69 | 26.61 | 81.05 | 31.58 | 1.22 | Large |
| FG1058 | 83.9 | 19.67 | 0.65 | 178.88 | 20.34 | 48.66 | 14.9 | 1.2 | Small |
| FG1060 | 52.09 | 12.79 | 0.59 | 272.06 | 16.92 | 29.01 | 6.15 | 1.46 | Small |
| FG1061 | 41.95 | 4.58 | 0.33 | 106.25 | 12.06 | 24.35 | 5.54 | 1.28 | Small |
| FG1063 | 18.46 | 4.98 | 0.57 | 190.63 | 8.77 | 9.34 | 0.35 | 1.22 | Small |
| FG1065 | 81.13 | 7.61 | 0.28 | 218.13 | 22.58 | 54.97 | 3.58 | 1.9 | Small |
| FG1066 | 139.11 | 18.13 | 0.37 | 289.88 | 33.46 | 94.18 | 11.47 | 1.96 | Large |
| FG1070 | 70.23 | 10.08 | 0.36 | 528.5 | 21.84 | 38.44 | 9.94 | 1.09 | Small |
| FG1071 | 82.79 | 9.2 | 0.34 | 183.63 | 20 | 58.67 | 4.12 | 1.92 | Small |
| FG1072 | 189.26 | 38.61 | 0.59 | 310.69 | 39.99 | 140.62 | 8.65 | 2.81 | Large |
| FG1077 | 103.74 | 11.06 | 0.3 | 445.25 | 25.75 | 73.48 | 4.51 | 1.9 | Small |
| FG1081 | 168.53 | 22.89 | 0.4 | 139.56 | 37.43 | 116.29 | 14.81 | 2.66 | Large |
| FG1082 | 94.7 | 10.58 | 0.3 | 524.94 | 26.76 | 63.75 | 4.18 | 1.9 | Small |
| FG1083 | 244 | 29.1 | 0.33 | 475.56 | 39.06 | 168.58 | 36.37 | 3.41 | Large |
| FG1084 | 103.07 | 11.25 | 0.31 | 438.38 | 28.92 | 67.71 | 6.44 | 2.58 | Small |
| FG1085 | 161.21 | 17.29 | 0.31 | 420.94 | 31.86 | 107.52 | 21.82 | 1.91 | Large |
| FG1088 | 120.88 | 27.11 | 0.58 | 435.13 | 29.16 | 84.32 | 7.4 | 2.36 | Medium |
| FG1090 | 49.43 | 7.14 | 0.33 | 653.38 | 16.02 | 27.21 | 6.21 | 1.21 | Small |
| FG1100 | 120.13 | 13.33 | 0.33 | 359.44 | 27.44 | 82.02 | 10.68 | 2.67 | Medium |
| FG1101 | 77.83 | 18.02 | 0.63 | 191.44 | 21.92 | 53.08 | 2.83 | 2.11 | Small |
| FG1102 | 82.97 | 19.55 | 0.6 | 409.31 | 18.91 | 58.54 | 5.52 | 1.98 | Small |
| FG1103 | 91.02 | 12.25 | 0.32 | 233.38 | 19.14 | 58.51 | 13.36 | 1.64 | Small |
| FG1104 | 38.91 | 5.56 | 0.39 | 136.13 | 14.07 | 21.49 | 3.34 | 1.43 | Small |
| FG1105 | 51.41 | 5.36 | 0.31 | 193 | 18.61 | 29.77 | 3.03 | 1.13 | Small |
| FG1108 | 83.04 | 18.66 | 0.55 | 422.56 | 20.26 | 54.83 | 7.95 | 2.17 | Small |
| FG1111 | 90.81 | 22.08 | 0.61 | 402.38 | 24.33 | 59.96 | 6.52 | 1.75 | Small |
| FG1112 | 114.61 | 11.97 | 0.3 | 390 | 24.97 | 83.65 | 5.99 | 1.92 | Small |
| FG1113 | 131.72 | 28.37 | 0.58 | 528.63 | 26.24 | 87.85 | 17.63 | 1.63 | Large |

**Supplementary Table 1e.** *Brassica oleracea*

| Genotypes # | TRL | TRSA | RAD | NTP | TPRL | TLRL | TTRL | BLL | Root Size |
| --- | --- | --- | --- | --- | --- | --- | --- | --- | --- |
| ECD11 | 261.81 | 31.23 | 0.38 | 1924.56 | 30.47 | 126.53 | 16.08 | 1.43 | Large |
| ECD13 | 155.96 | 11.37 | 0.23 | 243.13 | 32.8 | 113.07 | 4.98 | 1.38 | Large |
| FG467 | 97.17 | 10.04 | 0.32 | 150.19 | 19.88 | 45.15 | 32.14 | 1.31 | Small |
| FG485 | 139.19 | 13.19 | 0.29 | 287.69 | 29.59 | 69.69 | 39.91 | 1.55 | Large |
| FG503 | 161.55 | 31.05 | 0.45 | 1321.13 | 29.31 | 96.33 | 35.91 | 1.19 | Large |
| FG505 | 87.44 | 9.17 | 0.29 | 314.69 | 14.98 | 57.08 | 15.37 | 1.2 | Small |
| FG510 | 128.72 | 13.98 | 0.29 | 639.25 | 20.19 | 82.06 | 26.48 | 1.17 | Medium |
| FG514 | 161.81 | 16.47 | 0.28 | 287.19 | 29.79 | 74.51 | 57.52 | 1.79 | Large |
| FG518 | 88.03 | 10.29 | 0.32 | 333.63 | 19.43 | 53.03 | 15.57 | 1.98 | Small |
| FG533 | 174.92 | 17.4 | 0.28 | 286.38 | 27.8 | 86.72 | 60.4 | 1.82 | Large |
| FG534 | 162.36 | 16.62 | 0.31 | 400.69 | 23.53 | 58.59 | 80.25 | 1.54 | Large |
| FG535 | 150.33 | 13.01 | 0.26 | 298.5 | 25.78 | 72.63 | 51.93 | 1.45 | Large |
| FG536 | 187.59 | 17.12 | 0.28 | 258.63 | 22.18 | 77.24 | 88.16 | 1.92 | Large |
| FG538 | 197.75 | 17.25 | 0.27 | 323.56 | 30.76 | 94.58 | 72.4 | 2.38 | Large |
| FG539 | 165.34 | 35.2 | 0.45 | 1748.5 | 25.14 | 100.46 | 39.75 | 1.5 | Large |
| FG557 | 93.06 | 15.6 | 0.41 | 578.13 | 17.65 | 64.26 | 11.14 | 1.84 | Small |
| FG562 | 201.29 | 36.63 | 0.47 | 1032.25 | 29.5 | 137.66 | 34.13 | 2.12 | Large |
| FG565 | 161.56 | 26.77 | 0.41 | 948.88 | 29.32 | 108.56 | 23.68 | 1.72 | Large |
| FG570 | 201.34 | 18.29 | 0.28 | 257.69 | 32.78 | 146.91 | 21.64 | 1.66 | Large |
| FG577 | 170.14 | 15.11 | 0.26 | 460.63 | 28.05 | 115.04 | 27.06 | 1.81 | Large |
| FG582 | 360.36 | 60.11 | 0.47 | 1038.31 | 37.64 | 242.91 | 79.81 | 1.23 | Large |
| FG590 | 276.4 | 24.43 | 0.26 | 560.5 | 33.57 | 188.02 | 54.81 | 1.81 | Large |
| FG595 | 124.38 | 13.11 | 0.3 | 274.56 | 25.6 | 49.69 | 49.09 | 2.03 | Medium |
| FG597 | 149.62 | 16.99 | 0.32 | 258.06 | 25.08 | 65.14 | 59.4 | 1.1 | Large |
| FG598 | 176.9 | 33.77 | 0.43 | 1448.63 | 32.48 | 120.31 | 24.1 | 1.34 | Large |
| FG599 | 118.7 | 23.51 | 0.48 | 785.44 | 23.34 | 83.31 | 12.06 | 1.22 | Medium |
| FG614 | 317.2 | 24.35 | 0.29 | 474.81 | 25.44 | 228.72 | 63.04 | 1.56 | Large |
| FG616 | 235.26 | 43.35 | 0.43 | 1592.88 | 36.03 | 152.06 | 47.17 | 1.14 | Large |
| FG621 | 251.29 | 22.04 | 0.27 | 292.31 | 35.66 | 188.02 | 27.61 | 1.58 | Large |
| FG622 | 91.96 | 10.01 | 0.34 | 152.81 | 17.52 | 49.69 | 24.75 | 1.59 | Small |
| FG626 | 270.38 | 47.93 | 0.51 | 898.94 | 35.13 | 175.1 | 60.15 | 1.07 | Large |
| FG628 | 290.16 | 29.02 | 0.3 | 527.31 | 30.48 | 185.8 | 73.88 | 1.33 | Large |
| FG633 | 199.33 | 25.66 | 0.42 | 388.44 | 33.58 | 84.36 | 81.39 | 1.44 | Large |
| FG634 | 121.89 | 23.97 | 0.46 | 937.13 | 22.17 | 75.75 | 23.96 | 1.14 | Medium |
| FG635 | 172.58 | 18.12 | 0.32 | 388.25 | 27.13 | 79.7 | 65.74 | 1.79 | Large |
| FG636 | 105.04 | 18.52 | 0.41 | 1016.63 | 20.73 | 63.33 | 20.98 | 1.88 | Small |
| FG637 | 63.04 | 6.76 | 0.34 | 149.88 | 10.51 | 40 | 12.53 | 1.2 | Small |

**Supplementary Table 1e (continued).** *Brassica oleracea*

| Genotypes # | TRL | TRSA | RAD | NTP | TPRL | TLRL | TTRL | BLL | Root Size |
| --- | --- | --- | --- | --- | --- | --- | --- | --- | --- |
| FG640 | 69.4 | 14.97 | 0.41 | 1005.56 | 16.16 | 38.83 | 14.42 | 1.03 | Small |
| FG643 | 378.32 | 35.79 | 0.28 | 684.19 | 29.06 | 225.57 | 123.69 | 1.95 | Large |
| FG646 | 245.05 | 44.8 | 0.47 | 1201.75 | 34.88 | 169.45 | 40.71 | 1.68 | Large |
| FG647 | 282.41 | 48.92 | 0.45 | 1310.69 | 35.73 | 178.7 | 67.98 | 1.44 | Large |
| FG649 | 154.49 | 19.72 | 0.34 | 302.63 | 21.39 | 72.71 | 60.39 | 1.33 | Large |
| FG650 | 250.08 | 41.29 | 0.39 | 1475.44 | 33.95 | 156.44 | 59.69 | 1.3 | Large |
| FG651 | 139.76 | 13.69 | 0.28 | 229.31 | 27.85 | 68.43 | 43.48 | 1.63 | Large |
| FG653 | 72.1 | 14.16 | 0.49 | 575.44 | 16.3 | 46.33 | 9.46 | 1.14 | Small |
| FG654 | 112.78 | 9.94 | 0.27 | 229.38 | 25.1 | 75.03 | 12.65 | 1.78 | Small |
| FG655 | 173.22 | 16.86 | 0.3 | 265.19 | 20.04 | 118.54 | 34.63 | 1.83 | Large |
| FG656 | 59.85 | 8.74 | 0.34 | 453.5 | 16.66 | 37.88 | 5.32 | 1.32 | Small |
| FG657 | 100.78 | 16.86 | 0.43 | 648.69 | 16.74 | 78.48 | 5.56 | 0.66 | Small |
| FG658 | 81.29 | 17.6 | 0.44 | 866.69 | 19.16 | 53.61 | 8.53 | 1.36 | Small |
| FG659 | 176.74 | 32.04 | 0.41 | 1411.63 | 30.34 | 125 | 21.39 | 1.27 | Large |
| FG660 | 117.92 | 10.53 | 0.28 | 235.38 | 18.49 | 82.41 | 17.02 | 1.55 | Medium |
| FG661 | 97.11 | 19.55 | 0.5 | 562.19 | 20.7 | 62.98 | 13.42 | 1.38 | Small |
| FG662 | 178.99 | 16.31 | 0.28 | 402 | 27.44 | 122.97 | 28.59 | 1.47 | Large |
| FG663 | 96.14 | 18.8 | 0.52 | 540.63 | 15.92 | 65.62 | 14.6 | 0.94 | Small |
| FG679 | 225.89 | 22.9 | 0.29 | 321.56 | 34.94 | 165.43 | 25.52 | 1.45 | Large |
| FG851 | 87.94 | 12.85 | 0.32 | 771.06 | 20.5 | 53.86 | 13.59 | 1.27 | Small |

**Supplementary Table 1f.** *Brassica carinata*

| Genotypes # | TRL | TRSA | RAD | NTP | TPRL | TLRL | TTRL | BLL | Root Size |
| --- | --- | --- | --- | --- | --- | --- | --- | --- | --- |
| FG349 | 102.55 | 14.39 | 0.4 | 91.31 | 28.33 | 66.3 | 8.19 | 4.35 | Small |
| FG350 | 56.48 | 7.33 | 0.36 | 283.63 | 17.94 | 32.47 | 6.08 | 1.21 | Small |
| FG351 | 93.96 | 11.77 | 0.39 | 240.06 | 24.84 | 54.46 | 14.67 | 1.81 | Small |
| FG352 | 91 | 10.55 | 0.34 | 303.38 | 24.52 | 56.07 | 10.41 | 1.72 | Small |
| FG354 | 139.06 | 14.15 | 0.3 | 234.56 | 31.01 | 96.52 | 11.53 | 2.36 | Large |
| FG355 | 118.83 | 13.09 | 0.34 | 246.69 | 30.91 | 80.01 | 7.92 | 2.17 | Medium |
| FG357 | 56.95 | 14.98 | 0.58 | 383.13 | 21.48 | 28.92 | 6.55 | 1.57 | Small |
| FG358 | 86.1 | 16.17 | 0.47 | 455.19 | 24.97 | 50.95 | 10.19 | 1.47 | Small |
| FG360 | 64.87 | 9.05 | 0.4 | 268.63 | 18.01 | 37.67 | 9.19 | 0.82 | Small |
| FG361 | 48.37 | 13.26 | 0.76 | 147.69 | 12.48 | 27.37 | 8.51 | 0.96 | Small |
| FG364 | 54.01 | 15.21 | 0.67 | 315.5 | 18.04 | 29.94 | 6.03 | 2.16 | Small |
| FG366 | 163.6 | 15.62 | 0.3 | 308 | 33.5 | 117.07 | 13.03 | 1.23 | Large |
| FG368 | 51.92 | 6.84 | 0.36 | 144.13 | 17.78 | 30.76 | 3.38 | 1.56 | Small |
| FG371 | 123.13 | 13.33 | 0.32 | 424.94 | 28.44 | 85.22 | 9.47 | 2.4 | Medium |
| FG372 | 95.11 | 11.62 | 0.35 | 215.75 | 25.03 | 62.87 | 7.22 | 1.76 | Small |
| FG373 | 108.88 | 12.04 | 0.34 | 196.19 | 27.52 | 75.68 | 5.68 | 2.33 | Small |
| FG374 | 113.88 | 11.71 | 0.31 | 236.69 | 22.41 | 85.6 | 5.87 | 1.56 | Small |
| FG379 | 71.46 | 16.57 | 0.49 | 805.63 | 18.62 | 44.72 | 8.12 | 1.32 | Small |
| FG382 | 69.15 | 8.19 | 0.35 | 272.13 | 21.63 | 40.38 | 7.14 | 1.44 | Small |
| FG383 | 58.41 | 8.12 | 0.4 | 251.69 | 18.39 | 32.07 | 7.95 | 1.12 | Small |
| FG386 | 115.79 | 22.53 | 0.4 | 1189.81 | 30.35 | 74.15 | 11.28 | 1.99 | Medium |
| FG388 | 74.13 | 19.16 | 0.45 | 1128.56 | 23.86 | 40.8 | 9.46 | 1.16 | Small |
| FG390 | 68.18 | 16.96 | 0.55 | 486.63 | 20 | 39.05 | 9.13 | 1.21 | Small |
| FG391 | 162.63 | 14.43 | 0.27 | 392.63 | 34.34 | 111.39 | 16.9 | 1.85 | Large |
| FG393 | 64.13 | 15.22 | 0.53 | 523.94 | 22.66 | 36.08 | 5.4 | 1.09 | Small |
| FG394 | 49.67 | 7.39 | 0.4 | 334.06 | 17.04 | 27.68 | 4.95 | 1.1 | Small |
| FG395 | 115.85 | 13.65 | 0.35 | 356.19 | 25.6 | 76.76 | 13.5 | 2.54 | Medium |
| FG398 | 243.89 | 24.36 | 0.3 | 362.75 | 40.72 | 180.75 | 22.42 | 2.72 | Large |

**Supplementary Table 1g.** *Brassica nigra*

| Genotypes # | TRL | TRSA | RAD | NTP | TPRL | TLRL | TTRL | BLL | Root Size |
| --- | --- | --- | --- | --- | --- | --- | --- | --- | --- |
| FG224 | 121.66 | 19.62 | 0.43 | 275.06 | 19.91 | 80.04 | 21.72 | 1.79 | Medium |
| FG225 | 59.84 | 5.93 | 0.27 | 355.81 | 16.13 | 35.17 | 8.54 | 1.6 | Small |
| FG227 | 104.73 | 10.73 | 0.29 | 249.13 | 20.71 | 59.82 | 24.19 | 1.22 | Small |
| FG228 | 66.12 | 8.89 | 0.31 | 190.38 | 16.95 | 38.38 | 10.79 | 1.72 | Small |
| FG229 | 122.95 | 13.53 | 0.32 | 354.31 | 18.48 | 87.57 | 16.9 | 1.53 | Medium |
| FG230 | 153.48 | 23.39 | 0.36 | 831.56 | 28.48 | 111.73 | 13.28 | 1.75 | Large |
| FG231 | 115.13 | 12.3 | 0.33 | 366.56 | 21.65 | 75.87 | 17.6 | 1.22 | Small |
| FG234 | 112.63 | 17.64 | 0.35 | 560.88 | 23.09 | 72.87 | 16.68 | 1.48 | Small |
| FG240 | 92.23 | 9.04 | 0.28 | 255.56 | 21.82 | 55.53 | 14.88 | 1.3 | Small |
| FG241 | 71.41 | 7.7 | 0.28 | 340.69 | 19.91 | 43.87 | 7.64 | 1.48 | Small |
| FG242 | 43.3 | 5.93 | 0.31 | 210 | 13.72 | 24.84 | 4.74 | 1.91 | Small |
| FG243 | 54.13 | 5.62 | 0.28 | 419.31 | 19.42 | 30.63 | 4.08 | 1.64 | Small |
| FG245 | 54.13 | 5.62 | 0.28 | 419.31 | 19.42 | 30.63 | 4.08 | 1.64 | Small |
| FG246 | 73.82 | 6.64 | 0.28 | 258.94 | 25.63 | 43.95 | 4.24 | 1.33 | Small |
| FG247 | 60.18 | 7.37 | 0.35 | 143.81 | 16.66 | 38.72 | 4.8 | 1.57 | Small |
| FG248 | 54.48 | 11.23 | 0.49 | 306.75 | 14.94 | 32.56 | 6.98 | 0.88 | Small |
| FG249 | 61.83 | 6.16 | 0.3 | 276.19 | 21.74 | 34.4 | 5.68 | 1 | Small |
| FG250 | 55.4 | 4.97 | 0.26 | 257.44 | 16.22 | 34.84 | 4.34 | 0.87 | Small |
| FG251 | 62.46 | 6.93 | 0.32 | 119.13 | 25.58 | 33.72 | 3.16 | 1.99 | Small |
| FG252 | 63.84 | 6.13 | 0.27 | 299.19 | 23.81 | 35.64 | 4.39 | 1.8 | Small |
| FG253 | 60.47 | 5.53 | 0.25 | 480.94 | 19.53 | 34.92 | 6.02 | 1.68 | Small |
| FG262 | 53.45 | 5.37 | 0.27 | 250.38 | 20.49 | 29.73 | 3.23 | 2.12 | Small |
| FG263 | 72.82 | 11.39 | 0.48 | 180.06 | 18.51 | 47.11 | 7.2 | 1.39 | Small |
| FG265 | 108.08 | 13.25 | 0.32 | 602.25 | 31.03 | 69.65 | 7.4 | 1.43 | Small |
| FG269 | 121.05 | 10.63 | 0.27 | 243.44 | 34.85 | 82.18 | 4.02 | 1.48 | Medium |
| FG271 | 139.38 | 24.2 | 0.45 | 793.63 | 31.63 | 97.02 | 10.72 | 1.04 | Large |
| FG274 | 77 | 16.47 | 0.39 | 1143.13 | 30.81 | 41.87 | 4.33 | 1.03 | Small |
| FG276 | 85.62 | 9.39 | 0.31 | 272.88 | 21.07 | 57.44 | 7.11 | 1.21 | Small |
| FG281 | 86.7 | 6.68 | 0.24 | 212.25 | 25.4 | 58.47 | 2.83 | 1.25 | Small |
| FG285 | 186.46 | 18.15 | 0.3 | 369.63 | 33.2 | 138.62 | 14.64 | 2.49 | Large |
| FG286 | 31.86 | 3.23 | 0.26 | 285.19 | 14.78 | 15.59 | 1.5 | 1.38 | Small |
| FG287 | 46.58 | 10.82 | 0.59 | 217.5 | 16.41 | 27.77 | 2.4 | 1.58 | Small |
| FG289 | 42.35 | 4.65 | 0.31 | 110.38 | 15.02 | 22.13 | 5.19 | 1.97 | Small |
| FG291 | 97.47 | 19.18 | 0.38 | 1240.44 | 30.5 | 58.75 | 8.21 | 1.05 | Small |
| FG292 | 75.94 | 7.24 | 0.28 | 174.31 | 17.53 | 51.4 | 7.01 | 1.78 | Small |
| FG293 | 92.01 | 9.06 | 0.29 | 181.06 | 21.68 | 63.82 | 6.51 | 2.5 | Small |
| FG294 | 73.15 | 7.67 | 0.3 | 308.56 | 17.36 | 46.05 | 9.73 | 1.94 | Small |

**Supplementary Table 1g (continued).** *Brassica nigra*

| Genotypes # | TRL | TRSA | RAD | NTP | TPRL | TLRL | TTRL | BLL | Root Size |
| --- | --- | --- | --- | --- | --- | --- | --- | --- | --- |
| FG295 | 59.89 | 6.56 | 0.31 | 135.31 | 15.16 | 40.18 | 4.55 | 1.74 | Small |
| FG297 | 52.9 | 5.74 | 0.29 | 133.06 | 16.33 | 33.22 | 3.36 | 2.3 | Small |
| FG298 | 29.77 | 2.98 | 0.29 | 119.44 | 12.07 | 14.02 | 3.67 | 1.75 | Small |
| FG299 | 68.04 | 6.22 | 0.26 | 198 | 16.25 | 44.27 | 7.51 | 1.55 | Small |
| FG302 | 39.89 | 4.27 | 0.33 | 109.44 | 10.93 | 26.29 | 2.66 | 1.54 | Small |
| FG305 | 64.33 | 6.05 | 0.26 | 283.94 | 15.69 | 42 | 6.63 | 2.08 | Small |
| FG309 | 75.51 | 14.98 | 0.38 | 1051.25 | 29.32 | 42.11 | 4.08 | 0.86 | Small |
| FG312 | 141.05 | 13.22 | 0.28 | 186.88 | 28.91 | 98.44 | 13.7 | 3.43 | Large |
| FG316 | 127.58 | 11.88 | 0.28 | 273.75 | 32.77 | 89.82 | 4.99 | 2.71 | Medium |
| FG317 | 85.2 | 8.72 | 0.31 | 223.94 | 23.31 | 55.64 | 6.24 | 1.75 | Small |
| FG319 | 87.8 | 10.55 | 0.33 | 306.5 | 26.68 | 55.92 | 5.2 | 2.97 | Small |
| FG321 | 167.18 | 18.7 | 0.34 | 367.94 | 30.37 | 125.96 | 10.86 | 1.66 | Large |
| FG325 | 45.58 | 4.08 | 0.27 | 127.94 | 19.32 | 22.53 | 3.74 | 1.07 | Small |
| FG331 | 82.58 | 8.56 | 0.28 | 214.38 | 25.73 | 52.11 | 4.75 | 2.78 | Small |
| FG332 | 64.8 | 6.37 | 0.29 | 240 | 18.84 | 42.08 | 3.88 | 1.74 | Small |
| FG333 | 59.32 | 5.27 | 0.25 | 149.44 | 19.04 | 37.65 | 2.62 | 1.29 | Small |
| FG334 | 72.29 | 6.52 | 0.26 | 298.25 | 20.83 | 46.52 | 4.94 | 1.59 | Small |
| FG336 | 27.79 | 2.32 | 0.24 | 100.13 | 10.55 | 16.54 | 0.7 | 0.78 | Small |
| FG338 | 106.19 | 8.93 | 0.27 | 214.31 | 28.22 | 71.47 | 6.49 | 2.68 | Small |
| FG339 | 154.75 | 20.62 | 0.37 | 134.88 | 35.74 | 108.17 | 10.85 | 2.21 | Large |
| FG340 | 74.05 | 6.9 | 0.29 | 211.19 | 26.78 | 43.28 | 3.99 | 1.52 | Small |
| FG341 | 149.18 | 25.5 | 0.4 | 1114.75 | 35.46 | 105.79 | 7.92 | 1.71 | Large |
| FG342 | 53.36 | 4.79 | 0.27 | 109.69 | 16.58 | 32.4 | 4.38 | 2.03 | Small |
| FG343 | 74.1 | 7.9 | 0.31 | 135.38 | 19.19 | 46.66 | 8.25 | 1.89 | Small |
| FG344 | 70.36 | 7.01 | 0.27 | 288.25 | 27.49 | 38.81 | 4.05 | 1.43 | Small |
| FG345 | 85.55 | 7.35 | 0.26 | 183.69 | 27.31 | 54.35 | 3.89 | 2.44 | Small |
| FG346 | 79.92 | 11.98 | 0.37 | 117.13 | 28.44 | 48.07 | 3.41 | 2.34 | Small |
| FG347 | 140.09 | 24.61 | 0.41 | 1184 | 32.44 | 100.01 | 7.64 | 1.48 | Large |
| FG348 | 83.7 | 12.19 | 0.32 | 679.13 | 27.97 | 49.89 | 5.83 | 0.98 | Small |
